# Targeting *Pf*CLK3 with Covalent Inhibitors: A Novel Strategy for Malaria Treatment

**DOI:** 10.1021/acs.jmedchem.4c01300

**Published:** 2024-10-23

**Authors:** Skye B. Brettell, Omar Janha, Abbey Begen, Gillian Cann, Saumya Sharma, Niniola Olaniyan, Tamas Yelland, Alison J. Hole, Benazir Alam, Emily Mayville, Ross Gillespie, Michael Capper, David A. Fidock, Graeme Milligan, David J. Clarke, Andrew B. Tobin, Andrew G. Jamieson

**Affiliations:** † School of Chemistry, The Advanced Research Centre, University of Glasgow, 11 Chapel Lane, Glasgow G11 6EW, U.K.; ‡ Centre for Translational Pharmacology, The Advanced Research Centre, University of Glasgow, 11 Chapel Lane, Glasgow G11 6EW, U.K.; § KelticPharma Therapeutics, The Advanced Research Centre, University of Glasgow, 11 Chapel Lane, Glasgow G11 6EW, U.K.; ∥ Evotec (U.K.) Ltd, 95 Park Drive, Milton Park, Abingdon, Oxfordshire OX14 4RY, U.K.; ⊥ Department of Microbiology& Immunology and Center for Malaria Therapeutics and Antimicrobial Resistance, Division of Infectious Diseases, Department of Medicine, Columbia University Medical Centre, New York, New York 10032, United States; # EaSTCHEM School of Chemistry, University of Edinburgh, Joseph Black Building, David, Brewster Road, Edinburgh EH9 3FJ, U.K

## Abstract

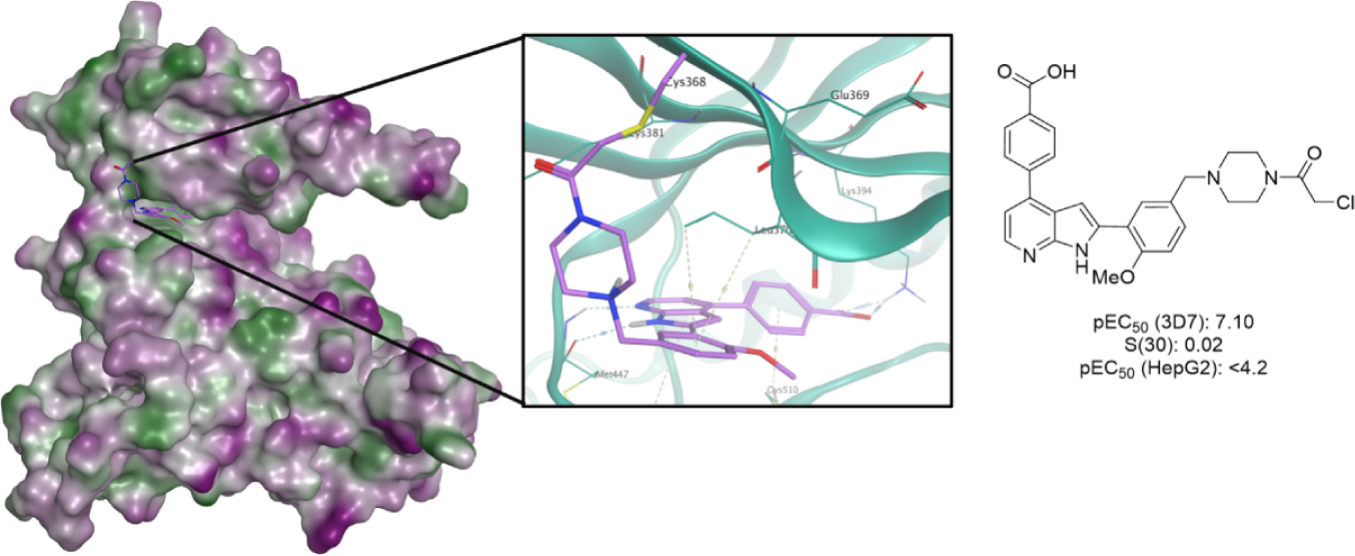

Malaria still causes over 600,000 deaths annually, with rising resistance to frontline drugs by *Plasmodium falciparum* increasing this number each year. New medicines with novel mechanisms of action are, therefore, urgently needed. In this work, we solved the cocrystal structure of the essential malarial kinase *Pf*CLK3 with the reversible inhibitor TCMDC-135051 (**1**), enabling the design of covalent inhibitors targeting a unique cysteine residue (Cys368) poorly conserved in the human kinome. Chloroacetamide **4** shows nanomolar potency and covalent inhibition in both recombinant protein and *P. falciparum* assays. Efficacy in parasites persisted after a 6 h washout, indicating an extended duration of action. Additionally, **4** showed improved kinase selectivity and a high selectivity index against HepG2 cells, with a low propensity for resistance (log MIR > 8.1). To our knowledge, compound **4** is the first covalent inhibitor of a malarial kinase, offering promising potential as a lead for a single-dose malaria cure.

## Introduction

Despite effective artemisinin-based combination therapies (ACTs), >240 million cases of malaria infection are reported annually resulting in >600,000 deaths.^1^ These numbers, although high, are a significant improvement on 2015 levels and represent some degree of success in the World Health Organisation “Global Technical Strategy (GTS)” aimed at reducing the global burden of malaria in 2030 by 90% from 2015 levels. Whereas the reduction in malaria cases can be attributed to the early success of the GTS, the last 5 years have seen little change in infection rates, and in some areas of the world, the trend has even been reversed and infections have increased. This worrying trend is exacerbated by the acquired resistance of the *Anopheles* mosquito vector to the insecticides used to impregnate bed nets and the emergence of parasite resistance to current frontline *Plasmodium falciparum* antimalarials, including ACTs.^2,3^ It is now widely understood that if the world is to avoid significant increases in cases of malaria, particularly of the most virulent human malaria species, (*P. falciparum*), new chemotherapeutic agents that act through a novel mechanism of action are urgently required.

**Figure 1:**
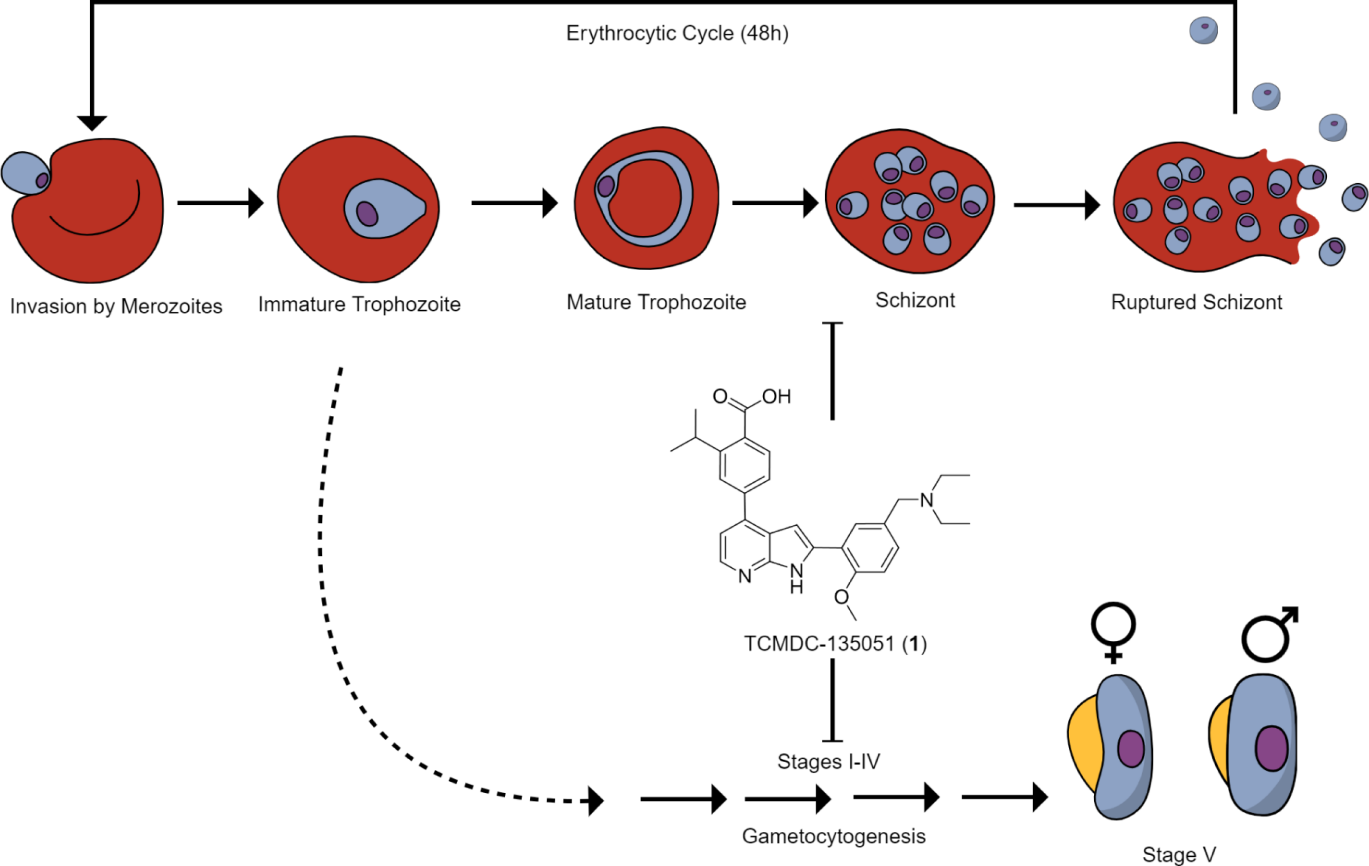
Inhibition of the *P. falciparum*life cycle at multiple stages by TCMDC-135051. Previous work^5^ has established TCMDC-135051 (**1**) as a curative, transmission blocking, and prophylactic agent, active in both asexual and sexual blood stages of the *P. falciparum* life cycle.

To address this, we have focused on targeting malarial parasite protein kinases that we identified as essential for blood stage parasite survival.^4^ Emerging from these studies has been a focus on the *P. falciparum* cyclin-dependent like protein kinase-3 (*Pf*CLK3), one of a family of four protein kinases with a role in the phosphorylation and assembly of components of the RNA spliceosome.^5^ A screen of the GlaxoSmithKline antimalarial-focused chemical library, called the Tres Cantos anti-malarial set (TCAMS), identified the compound TCMDC-135051 (**1**) (Figure 1) as a selective *Pf*CLK3 inhibitor. This tool inhibitor was used in combination with genetically engineered parasite lines and field isolates to validate *Pf*CLK3 as a target with the potential to deliver a cure for blood stage infection as well as preventing the development of gametocytes responsible for transmission and parasiticidal activity of liver stage in a manner that can be prophylactic. TCMDC-135051 (**1**) has since entered a drug development program aimed at generating a clinical candidate that is curative, transmission blocking and offering prophylaxis across*Plasmodium* sp.^6^

One of the major challenges faced in developing next generation antimalarials is the requirement to produce a single dose medicine that is highly tolerated and safe to be administered to young children and pregnant women. The erythrocytic stage of the parasite has a 48 h cycle, where the parasite can sequester in tissues, such as bone marrow; the gametocyte stages can take many days to develop; and stage V gametocytes remain in the circulation for several weeks, implying that any effective antimalarials need to act at multiple stages of the parasite life cycle for long periods.^7−9^ Inhibitors of *Pf*CLK3 are effective at multiple parasite stages, correlating with the importance of RNA-splicing in the biology of the parasite, the question of long exposure at the target is an important issue.^5^ A potential strategy to deliver extended exposure is to build in favorable pharmaco-dynamic properties through the application of covalent inhibitors, which bind irreversibly to the target.^10^ This approach has been employed in targeting protein kinases in oncology, where covalent inhibitors have shown increased potency, selectivity, and decreased propensity to resistance. Despite the unquestionable success of targeting protein kinases in cancer, the exploitation of protein kinase inhibitors in malaria is in its infancy.^11^ What has certainly never been explored is the potential of covalent kinase inhibitors as effective antimalarials.^12,13^

Here, we employ a high-resolution atomic structure of TCMDC-135051 (**1**) in complex with *Pf*CLK3 to inform the structure-guided design of a covalent inhibitor that targets a cysteine residue, proximal to the catalytic site of *Pf*CLK3, which is poorly conserved across the human kinome. Protein mass spectrometry and live parasite washout experiments confirmed successful covalent modification of the target cysteine. The covalent *Pf*CLK3 inhibitor showed multistage parasite potency, as well as significantly improved selectivity over the human kinome and a more favorable cytotoxicity profile in HepG2 cells when compared to the parent molecule TCMDC-135051 **1**. A high MIR (minimum inoculum for resistance) was shown. We conclude that a covalent binding mechanism for protein kinase inhibitors targeting essential malarial protein kinases could provide the pharmacodynamic and parasiticidal properties desired in a strategy for the development of a single-dose cure for malaria.^12,14^

## Results

### X-Ray Crystal Structure Reveals the Mechanism of TCMDC-135051 (**1**)/*Pf*CLK3 Binding and Facilitates Structure-Based Covalent Inhibitor Design

**Figure 2:**
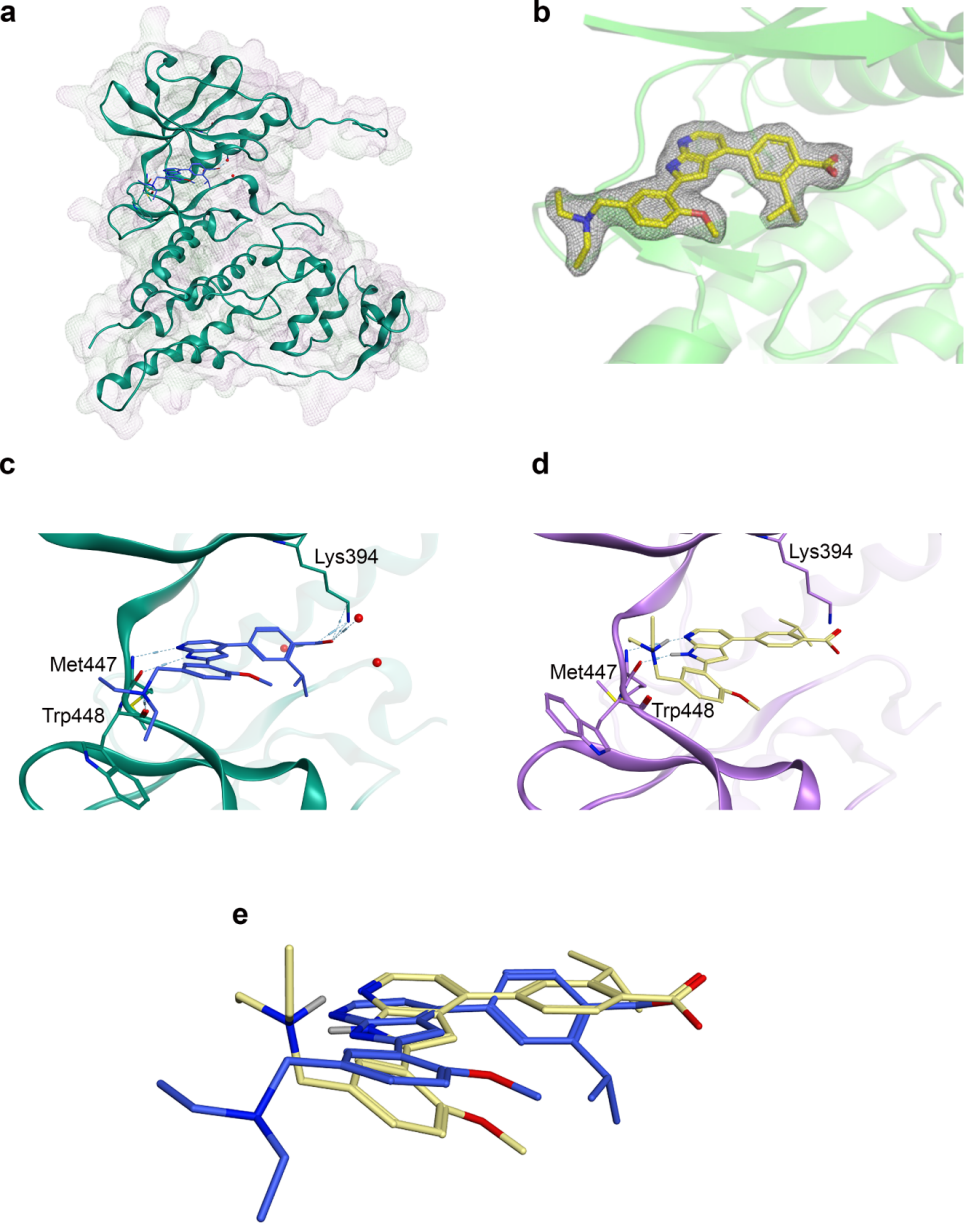
Mechanism of *Pf*CLK3 inhibition by TCMDC-135051. **(a**) Co-crystal structure of *Pf*CLK3 (teal) in complex with TCMDC-135051 (blue) (PDB: 8RPC). Protein surface mesh visualized in MOE, hydrophobic patches in green, and hydrophilic in lilac. (b) Electron density map for TCMDC-135051 (yellow) in the ATP binding site (green). (c) ATP binding site interactions of TCMDC-135051 (blue) evident in cocrystal structure with *Pf*CLK3 (teal). (d) Molecular docking of TCMDC-135051 (**1**, yellow) in a previously published homology model of *Pf*CLK3 (lilac).^6^ (e) Overlay of TCMDC-135051 (**1**) binding pose from8RPC(blue) and molecular docking (yellow).

To establish the binding mode of the tool *Pf*CLK3 inhibitor TCMDC-135051 (**1**) we report here a cocrystal structure of *Pf*CLK3 kinase domain in complex with TCMDC-135051 (**1**) to 2.1 Å resolution (Figure 2a,b, PDB: 8RPC). The structure resembles our previously published molecular modeling of TCMDC-135051 (**1**) in a homology model (generated using SWISS-MODEL and the kinase domain structure of the closest mammalian orthologuePRPF4B as a template) with an RMSD of 1.08 Å, yet with several important differences (Figure 2c,d).^5^ The azaindole scaffold of TCMDC-135051 (**1**) binds as predicted to the ATP-binding site, in the flipped orientation, forming hydrogen bonds with the amide backbone of Met447 (Figure 2c,d).^15^ However, the core sits closer to the hinge loop and farther out of the binding site than predicted, which allows the diethylamine to form a charge-dipole interaction in the crystal structure with the backbone carbonyl of Trp448 (Figure 2c–e). The carboxylic acid–Lys394 interaction predicted in the docking studies is mediated by three water molecules as seen in the crystal structure (Figure 2c), which sit in the pocket forming a network of interactions with three other residues—Cys510, Asp511, and Ser377 (Figure S6). Interestingly, the isopropyl group does not displace these waters to sit in the hydrophobic pocket next to the Phe444 gatekeeper, as predicted (Figure 2d). Surprisingly, this lipophilic functionality appears to protrude out of the pocket toward the solvent exposed space (Figure 2c).

**Figure 3:**
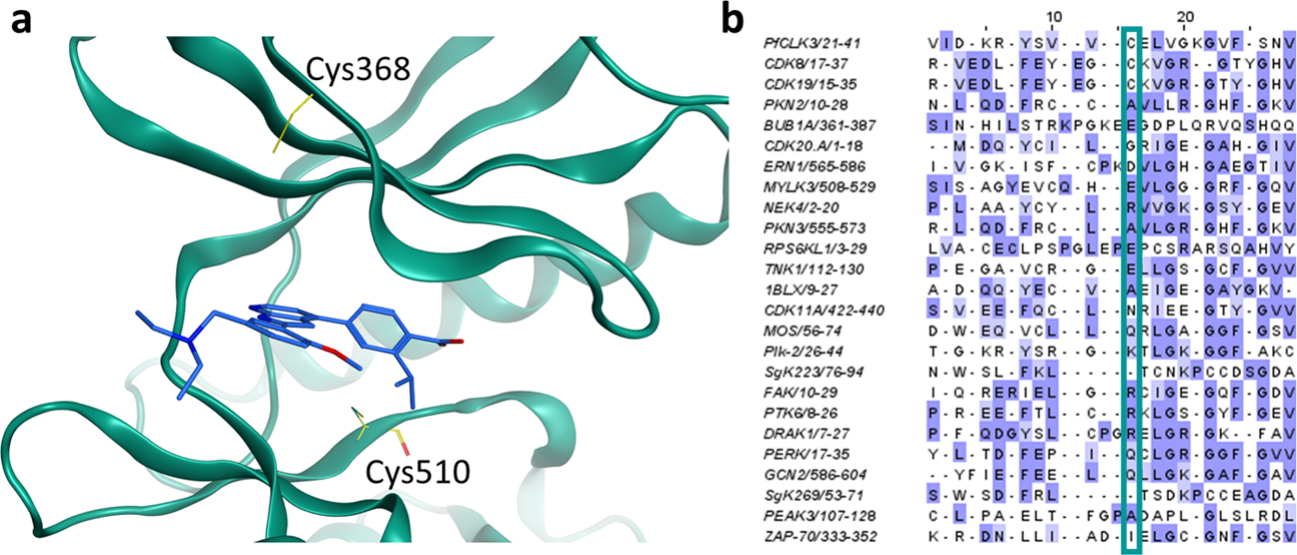
Potential *Pf*CLK3 cysteine residue targets. (a) Cysteine residues (yellow) located near the binding site of TCMDC-135051 (**1**, blue) in *Pf*CLK3 (teal). Cys510 is the DFG-1 residue located on the activation loop and Cys368 is located adjacent to the P-loop. (b) Sequence alignment of all P-loop cysteines in the human kinome against *Pf*CLK3 (row 1, teal box). Only 2 kinases, CDK8 and CDK19 (rows 2 and 3, teal box), possess cysteines in locations equivalent to Cys368 of *Pf*CLK3. Cys368 resides in an allosteric site, outside the ATP-pocket, toward the N terminus of the P-loop.

Using this structure, two cysteines were identified within or near the ATP binding site (Figure 3a): Cys368 of the P-loop and Cys510, the DFG-1 residue of the activation loop.^16^ Sequence and structural alignments of *Pf*CLK3 and a set of 497 human kinases were completed using the resource KINCORE and the Molecular Operating Environment (MOE) (Figure 3b).^17,18^ All cysteines within the P-loop of the human kinome set were selected in KINCORE and the structures of these kinases loaded and aligned with *Pf*CLK3 in MOE. This analysis established that Cys510 (DFG-1) of *Pf*CLK3 has 45 equivalent cysteines within the human kinome set, whereas Cys368 has only 2 (hCDK8 and hCDK19, Figure 3b). Cys368 was therefore chosen as the more attractive nucleophilic residue for a covalent inhibitor to confer selectivity.

**Figure 4:**
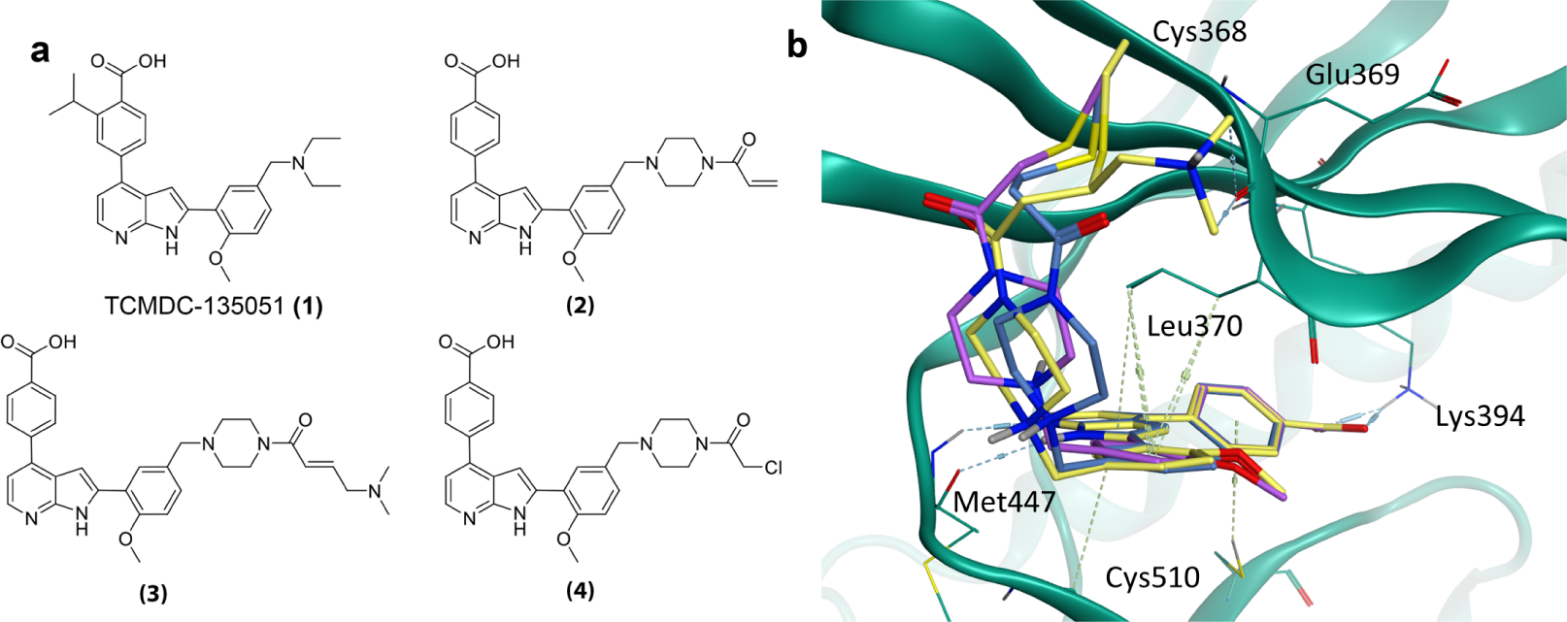
Compound design and molecular docking. (a) Clockwise from top left- TCMDC-135051 (**1**), unsubstituted acrylamide **2**, basic dimethylamino acrylamide **3**, and chloroacetamide **4**. (b) molecular docking of compounds **2** (blue), **3** (yellow), and **4** (purple) in cocrystal structure of TCMDC-135051 and *Pf*CLK3.

A series of analogues was designed featuring warheads of increasing reactivity (Figure 4a). Compound **2** features an acrylamide, the most common of the electrophiles due to its low reactivity.^19,20^ Compound **3** features a basic amino-acrylamide warhead, while compound **4** incorporates an α-chloroacetamide.^21,22^ These warheads show increasing reactivity when matched molecular pairs are reacted with glutathione, with the α-chloroacetamide being the most reactive.^19^ All analogues were then docked into the cocrystal structure (8RPC). **2**-**4** were predicted to maintain the binding mode of TCMDC-135051 (**1**), forming the same key interactions discussed above, while projecting sufficiently out of the pocket to form covalent bonds with Cys368 (Figure 4b).

### Chemical Synthesis of Covalent Inhibitors

**Scheme 1:**
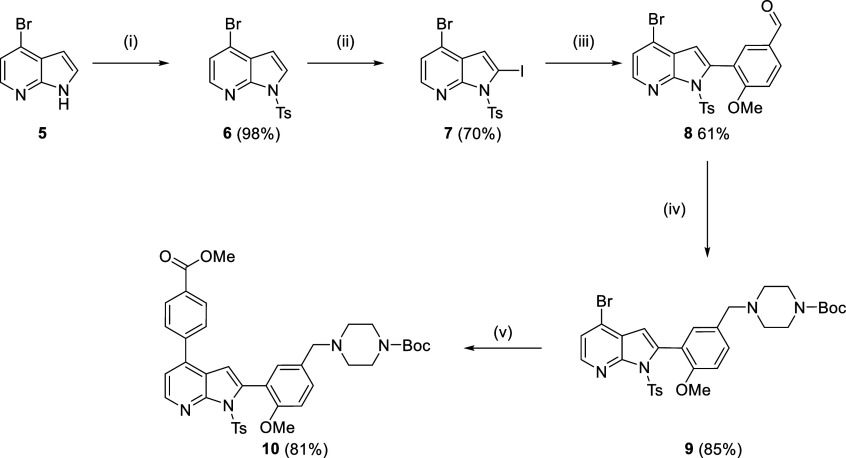
Synthesis of Key Intermediate **10** (i) TsCl, NaH, dichloroethane, 0 °C, rt, 1h; (ii) LDA, I_2_, THF, −78 °C, 2.5 h; (iii) (5-formyl-2-methoxyphenyl)boronic acid, Pd(PPh_3_)_4_, Na_2_CO_3_, 1,4-dioxane, 110 °C, 18 h; (iv) *N*-Boc-piperazine, NaBH(AcO)_3_, dichloroethane, rt, 18 h; (v) 4-(4,4,5,5-tetramethyl-1,3,2-dioxaborolan-2-yl)benzoic acid, Pd(dppf)Cl_2_·CH_2_Cl_2_, Na_2_CO_3_, 1,4-dioxane, 110 °C, 0.5 h, μW.

Compounds **2**-**4** were synthesized from a common intermediate, the Boc-amine protected methyl ester compound **10**. Compound **10** was obtained from a 5 step synthesis (Scheme 1) based on that of the hit compound TCMDC-135051 (**1**).^6^ Tosylation of commercially available 4-bromoazaindole **5** produced azaindole **6** in 98% yield. Selective iodination of the indole C-2 position was achieved through directed metalation to provide iodide **7** which was obtained in good yield. Suzuki coupling of iodoazaindole **7** with 5-formyl-2-methoxyphenyl boronic acid gave aldehyde **8**. Reductive amination installed the Boc-protected piperazine linker to give compound **9** in 85% yield, followed by a second Suzuki coupling giving **10** in 81% yield.

**Scheme 2:**
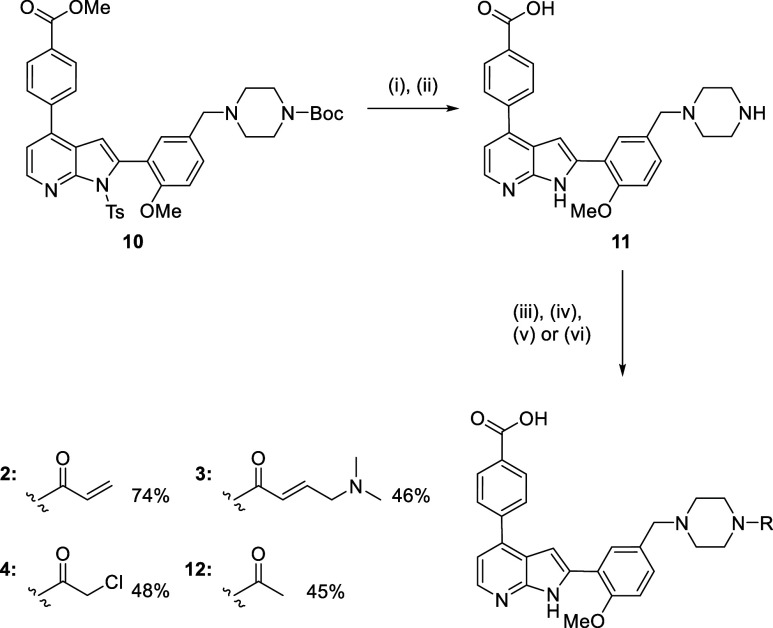
Synthesis of Compounds **2**–**4** and **12** (i) TFA, dichloroethane, rt, 1.5–18h; (ii) KOH, MeOH, H_2_O reflux; (iii) acryloyl chloride, NEt_3_, dimethylformamide, rt, 1h; (iv) (*E*)-4-(dimethylamino)-2-butenoic acid hydrochloride, SOCl_2_, NMP, rt, 1.3h; (v) chloroacetyl chloride, NEt_3_, dimethylformamide, rt, 2h; (vi) acetyl chloride, NEt_3_, dimethylformamide, rt, 2h.

After a two-stage global deprotection of **10** (potassium hydroxide in methanol, followed by treatment with trifluoroacetic acid), treatment of **11** with acryloyl chloride and triethylamine gave **2** in 74% yield (Scheme 2). Compounds **4** and **12** were obtained using the same procedure with chloroacetyl chloride and acetyl chloride, giving the desired compounds in 48% and 45% yield, respectively. Coupling of **11** and (*E*)-4(dimethylamino)-2-butenoic acid hydrochloride using thionyl chloride afforded compound **3** in 46% yield.

### Mass Spectrometry Reveals Specific Covalent Modification of the Target Cysteine

**Figure 5:**
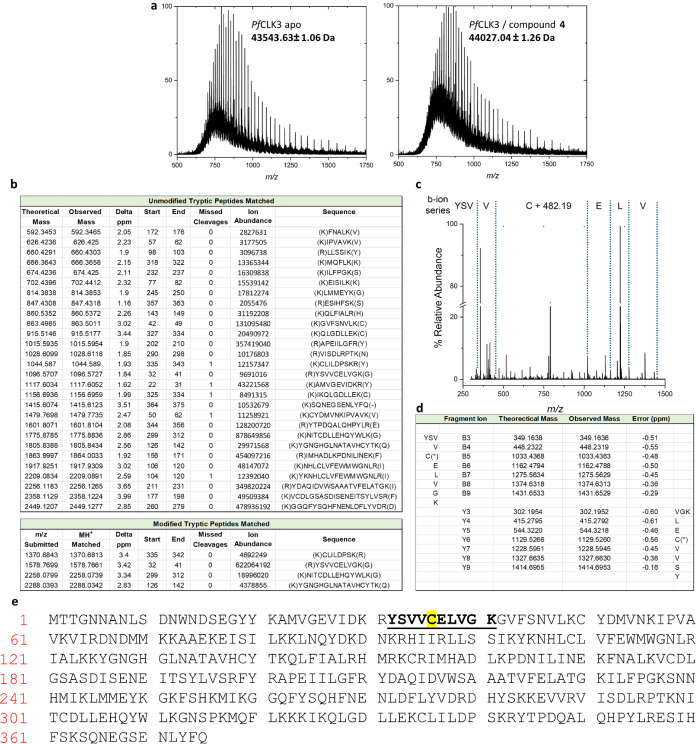
Protein mass spectrometry of *Pf*CLK3 and compound 4. (a) Intact protein mass spectrometry of apo *Pf*CLK3 and *Pf*CLK3 incubated with compound **4** for 4 h. A mass difference of 483.41 can be observed, corresponding to the mass of compound **4** minus the chloride leaving group. (b) Table of unmodified and modified peptides obtained from the tryptic digest. (c) CID fragmentation spectrum of the most abundant modified peptide, YSVVCELVGK which contains Cys368. (d) Table of fragmentation ions masses quoted as the monoisotopic neutral mass. (e) The sequence of the *Pf*CLK3 kinase domain with the modified peptide in bold and Cys368 highlighted in yellow.

Covalent adduct formation was investigated by intact protein mass spectrometry. Apo protein kinase domain was compared with samples that had been incubated with compounds **2**, **3**, and **4** at varying pHs. These experiments were performed on the *Pf*CLK3 kinase domain (334–699) given that the full-length recombinant protein did not ionize using ESI TOF analysis. At physiological pH 7.4, no covalent adduct formation was observed for compound **2** after 4 h. When the pH was raised to 9, adduct formation was observed. This implies a lack of warhead reactivity, with basic conditions required to deprotonate Cys368, increasing nucleophilicity and driving product formation. Cysteine reactivity is governed by the side chain p*K*_a_, which can vary from 3.5 to 12 depending on specific protein microenvironments.^23,24^ Cys368 can therefore be considered to be relatively weakly nucleophilic. The more reactive substituted acrylamide **3** demonstrated no adduct formation at pH 7.4, while the most reactive chloroacetamide **4** demonstrated 100% singular adduct formation (Figure 5a).

The nucleophilic target residue was then determined by tandem mass spectrometry (Figure 5b–e). After incubation with compound **4**, *Pf*CLK3 was digested with trypsin. The resulting peptides were then analyzed by electrospray ionization mass spectrometry and compared with the list of expected peptides using Protein Prospector.^25^ This yielded four peptides modified by the expected monoisotopic mass of compound **4** minus Cl (Figure 5b), with one principal peptide being >10-fold greater in abundance, YSVVCELVGK. This peptide corresponds to residues 364–373 (Figure 5e). This ion was then further fragmented by CID to reveal a single modification of Cys368 (Figure 5c,d). Given that chloroacetamide **4** appears to form only one covalent adduct in the intact mass spectrometry (Figure 5a), and that modified peptide YSVVCELVGK is detected in much greater ion abundance than other modified species (Figure 5b), this implies selectivity for Cys368. This is presumably aided by the pseudohigh concentration in the ATP binding site due to the reversible interactions of the ligand.

### *In Vitro* Potency Against Recombinant *Pf*CLK3 Demonstrates Improved Activity for Covalent Binding Mode

**Figure 6:**
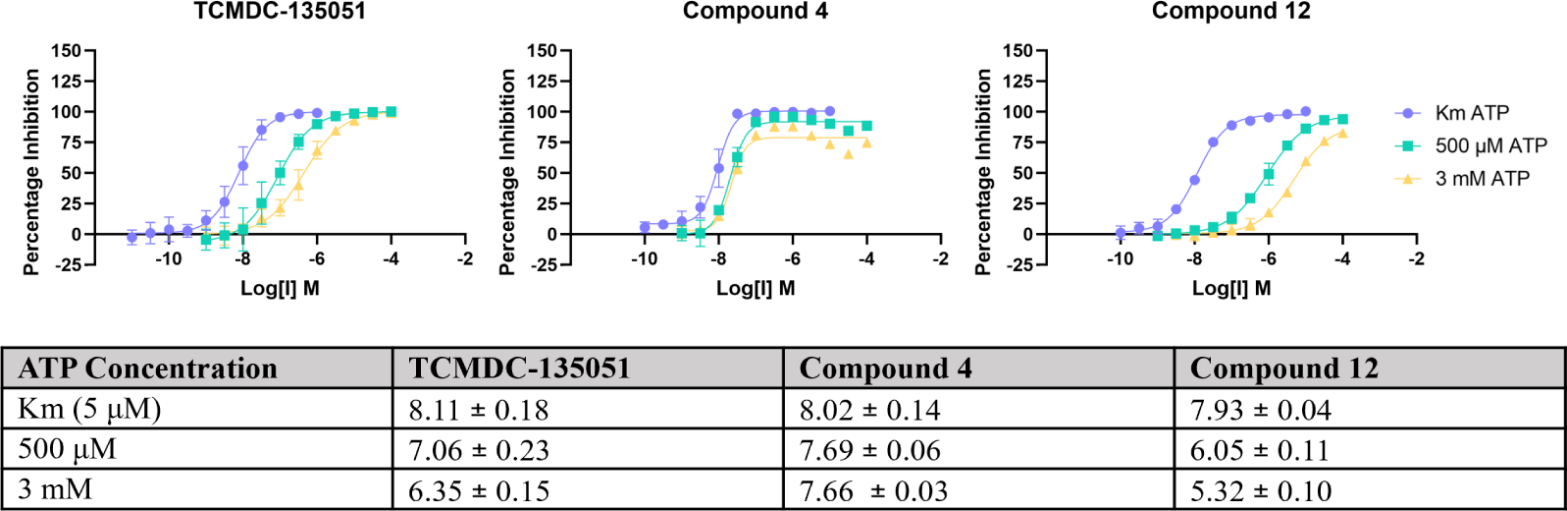
*In vitro* activity of compounds **4**, **12**, and TCMDC-135051 (**1**) against recombinant *Pf*CLK3 shows maintained potency for covalent compound 4 when ATP concentrations reflect cellular levels. Each compound was tested for inhibitory activity with ATP concentrations = *K*_m_ (5 μM, purple), 500 μM (teal), and 3 mM (yellow). While TCMDC-135051 (**1**, left) and compound **12** (right) lost potency with increased ATP concentrations, compound **4** retained its potency. Apparent *p*IC_50_ values and their standard deviations are given in the table. Apparent *p*IC_50_ values are obtained after a 15 min preincubation and 2 h incubation time.

Compounds **1**, **4**, and **12** were then evaluated for inhibitory activity against recombinant full-length *Pf*CLK3 in an *in vitro* TR-FRET protein kinase assay (Figure 6). To test the effect of covalency, three different concentrations of the natural substrate ATP were used: 5 μM (*K*_m_), 500 μM, and 3 mM (to mimic cellular levels).^26^ The hypothesis being that once a covalent inhibitor has bound to the target protein, it cannot be outcompeted by ATP. Biochemical potencies demonstrated exactly this: while compound **4** and its noncovalent control exhibited comparable potencies to TCMDC-135051 when [ATP] = *K*_m_, (*p*IC_50_ = 8.02 and 7.93, *p* = 0.88 and 0.37, respectively), TCMDC-135051 and noncovalent compound **12** both decreased in potency when ATP concentrations rose. TCMDC-135051 demonstrated decreased potencies of 7.06 (*p* < 0.0001 wrt *K*_m_) and 6.35 (*p* < 0.0001 wrt *K*_m_) for 500 μM and 3 mM ATP respectively, with compound **12** showing a similar trend (*p*IC_50_ = 6.05 and 5.32, *p* < 0.0001 wrt *K*_m_). Chloroacetamide **4** however maintained its high potency in all assays, where *p*IC_50_ = 7.69 (*p* = 0.1158 wrt *K*_m_) and 7.66 (*p* = 0.0658 wrt *K*_m_) for [ATP] = 500 μM and 3 mM, respectively. While ATP noncompetitive data may often suggest an allosteric binding mode, the comparison with ATP-competitive compound **12**, combined with mass spectrometry data, is indicative of a covalent binding mode for compound **4**. It is supposed this binding mode may be advantageous relative to TCMDC-135051 in cellular assays, when ATP concentrations rise to 1–3 mM.^26^ Chloroacetamide **4** was also evaluated by using a thermal shift assay. In the presence of an excess of **4**, full-length *Pf*CLK3 was highly thermodynamically stable, with a 20 °C shift in *T*_m_ compared to the DMSO control (Table S2). This large shift demonstrates an impressive stabilization effect of chloroacetamide **4**’s binding to *Pf*CLK3.

### Evaluation of Parasiticidal Activity of Compounds **1** and **4** Confirms Covalent-Based Mechanism of Action

**Figure 7:**
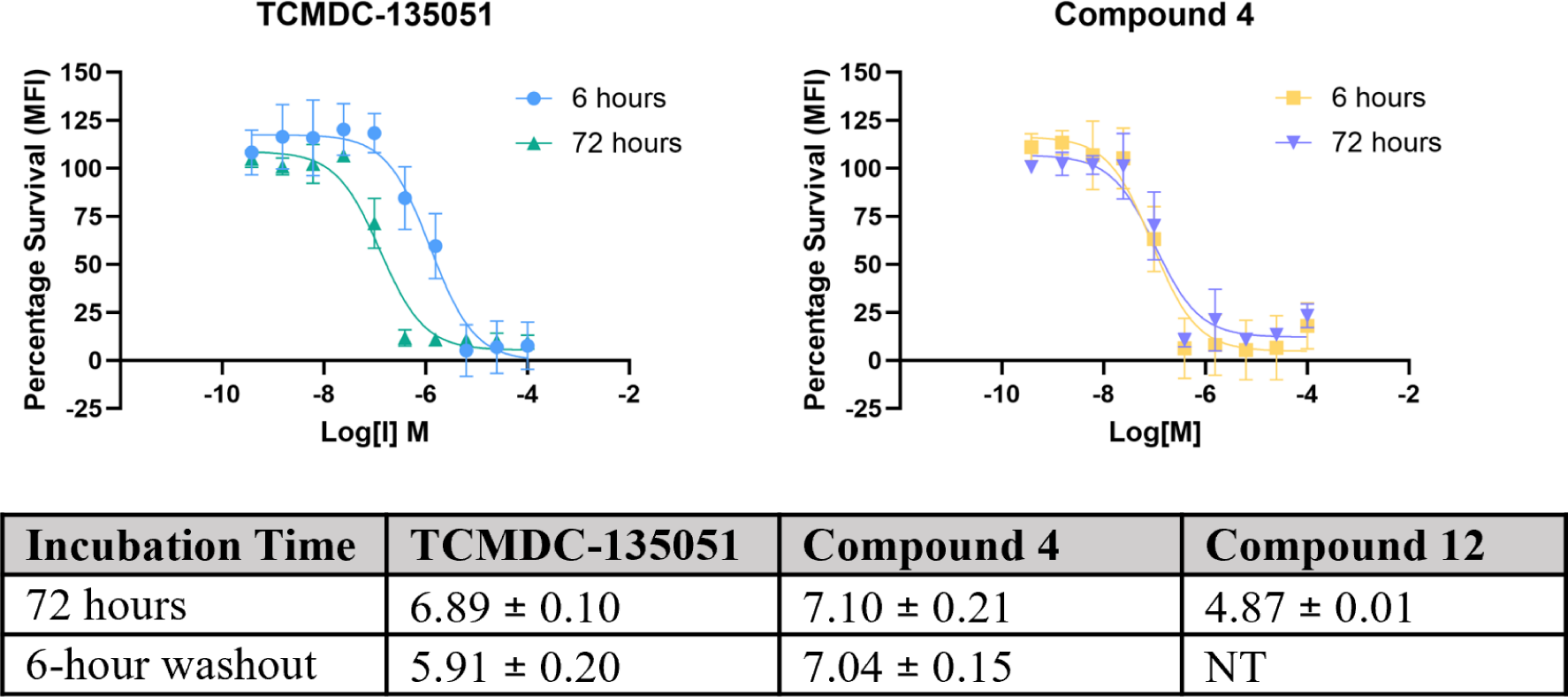
Parasiticidal activity of TCMDC-135051 (**1**) and compound **4** reflects covalent binding mechanism and extended duration of action in cells. Compounds **1** and **4** were incubated with ring-stage parasites for 72 h (teal and lilac, respectively). In washout studies (blue and yellow, respectively), compound medium was exchanged for compound-free medium after 6 h, and parasites were incubated for a further 66 h. While TCMDC-135051 (left) showed reduced potency after washout compared to the 72 h incubation, compound **4** (right) maintained its potency. *p*EC_50_ values and standard deviations are given in the table. NT = Not tested

Covalent chloroacetamide inhibitor **4** and its noncovalent control analogue **12** were next evaluated for parasiticidal activity in *P. falciparum* 3D7 cell line (Figures 7 and S2). After incubation with ring-stage parasites for 72 h, chloroacetamide **4** exhibited a half-maximal response concentration (*p*EC_50_) of 7.10. Compound **4** therefore has comparable potency to TCMDC-135051 (**1**) (*p*EC_50_ = 6.89, *p* = 0.5669) and a significant increase in potency compared to the noncovalent control compound **12** (*p*EC_50_ = 4.87, *p* < 0.0001, Figure S4).

It was predicted that a covalent inhibitor may need only a short exposure to have a prolonged parasiticidal effect, while a noncovalent inhibitor would be less active after a short exposure. To test this notion ring stage parasites were exposed for 6 h only with compound **4** (covalent), or TCMDC-135051 (**1**) (noncovalent). Following compound washout, the parasite culture was continued for 66 h, and parasite viability tested. Under these conditions the potency of TCMDC-135051 (**1**) significantly reduced (*p*IC_50_ = 5.91, *p* = 0.0003) while the potency of compound **4** was not reduced following wash out (*p*IC_50_ = 7.04, *p* = 0.9439). These data are consistent with a covalent mechanism of action in parasite cells for chloroacetamide inhibitor **4**. Furthermore, the demonstration of a short exposure time resulting in a prolonged parasiticidal effect is a useful finding in the quest for a single-dose cure for malaria.

### In Contrast with Artemisinin, Chloroacetamide **4** Demonstrates Constant Activity Against Different Stages of Asexual *P. falciparum* Parasites

To determine the stage of parasite inhibition of compound **4** against asexual *P. falciparum* 3D7 parasites, its potency and time of kill at early-ring-stages (0–3 h post invasion, hpi); late-ring-stage old parasites (16 hpi ± 2 h); midtrophozoite stages (24 hpi ± 2 h); and schizont-stage parasites (40 hpi ± 2 h) was evaluated.

**Figure 8:**
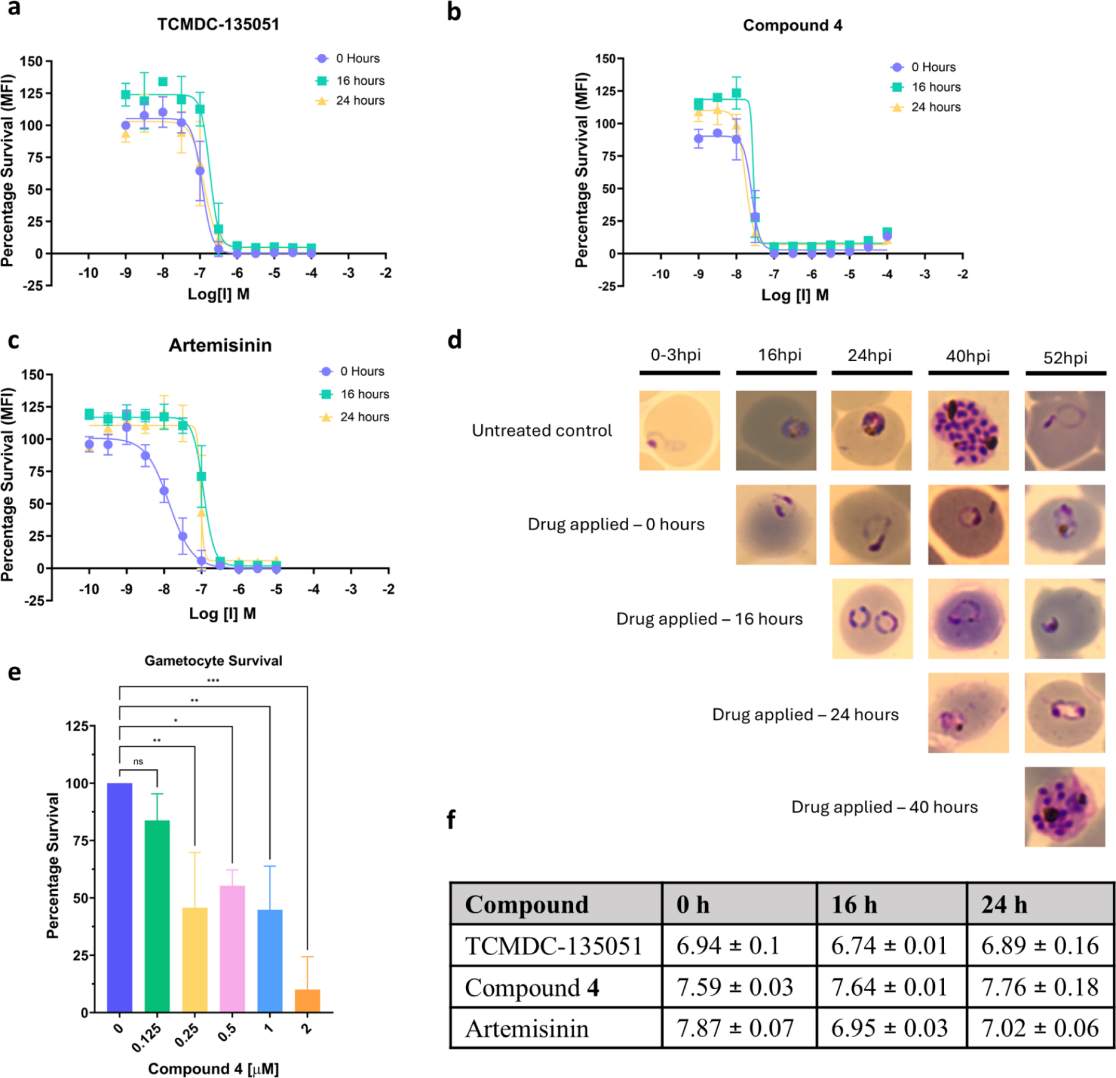
Life stage activity of compounds, sexual and asexual. (a–c,) dose response curves of compounds **1**, **4**, and artemisinin when dosed at different stages of asexual blood stage 3D7 parasites (early-ring, immature, and mature trophozoite). (d) phenotypic response to compound **4** dosed at different stages of asexual blood stage 3D7 parasites (early-ring, immature and mature trophozoite, and schizont) at a concentration of 1 μM (8 × EC_50_). (e) Gametocyte (sexual stage parasites) survival for differing concentrations of compound **4**. *p < 0.05, ** *p* < 0.01, ***p < 0.001. (f) pEC50 values attributed to a–c and standard deviations.

Parasites treated with compound **4** at different time points have similar dose response curves with similar *p*EC_50_ (Figure 8b,f). Comparable results are seen for TCMDC-135051 (Figure 8a,f). These results demonstrated that both TCMDC-135051 and chloroacetamide inhibit asexual parasites, irrespective of the stage of dosage. This is consistent with *Pf*CLK3′s multistage role in the parasite life cycle. However, when the same parasite preparations were incubated with artemisinin, potency was reduced by approximately one log comparing treatment of young rings (0–3 hpi) to older parasite stages (16–40 hpi; Figure 8c,f). Artemisinin is not multistage active in asexual parasites, highlighting the potential of *Pf*CLK3 inhibitors in antimalarial drug discovery.

The phenotypic response to compound **4** is shown in Figure 8d. When treated with **4** at the early ring stage (0–3 hpi), parasites did not progress to immature trophozoite, as compared to the untreated control, which went through the full blood stage life cycle. These arrested early ring parasites appeared shrunken and condensed, indicating cell death; immature trophozoite dosed at 16 h did not then mature. The same was true for mature trophozoites treated at 24 h, which did not become schizonts. When schizonts were treated at 40 h, they did not egress to form new rings. Compound **4** therefore arrests development and kills parasites at every stage in the erythrocytic life cycle. This is consistent with our previously reported data for TCMDC-135051 (**1**), showing multistage activity for *Pf*CLK3 inhibitors in asexual 3D7 parasites.

### Compound **4** Kills Sexual Stage Parasites, Showing Transmission-Blocking Potential

To evaluate the transmission blocking capability of the covalent inhibitor chloroacetamide, inhibition of gametocyte development in the presence of the inhibitor was evaluated as proxy for transmission.

As seen in Figure 8e, using the asexual parasite EC_50_ concentration, no significant reduction in gametocyte development/maturation is observed compared to untreated control. However, using 2× EC_50_, 4× EC_50_, and 8× EC_50_, a significant reduction (∼50% decrease) in the number of mature gametocytes was observed, demonstrating gametocytocidal activity of the covalent inhibitor. In the highest concentration tested, 2 μM, visible mature stage V gametocytes were reduced to approximately 10%. In a real-world scenario, such a reduction would result in substantial reduction in potential gametocyte carriers and therefore probability of infecting mosquitoes would also reduce. The use of a covalent inhibitor has potential to prolong this activity as seen in the asexual stages (Figure 7), which may persist into the mosquito midgut preventing exflagellation, further impacting transmission.

### Covalent Targeting of Cys368 Leads to an Increase in Kinome Selectivity

**Figure 9:**
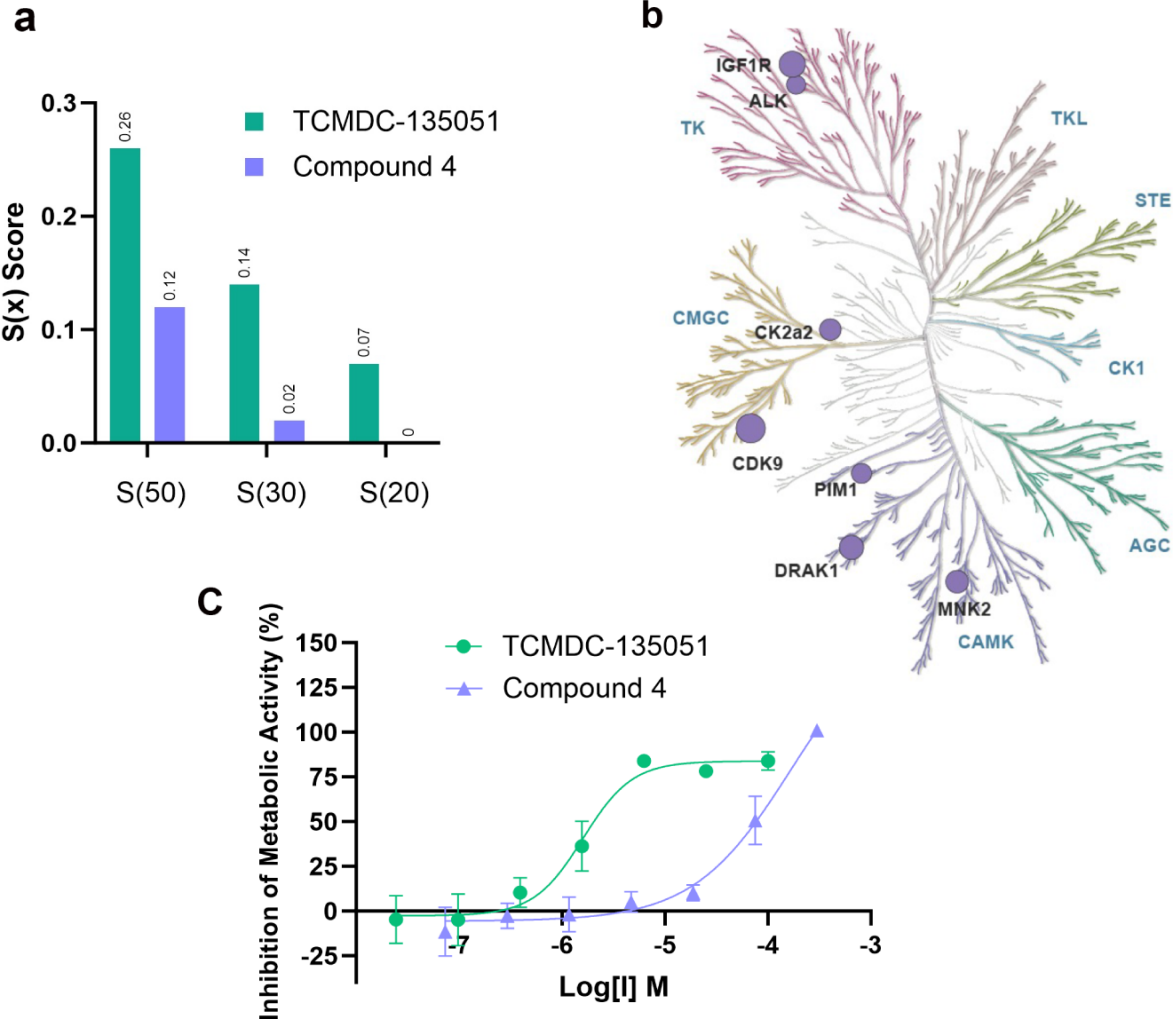
Compound **4** shows excellent selectivity profile compared to TCMDC-135051 (**1**). (a,) Selectivity scores of compound **4** and TCMDC-135051 (**1**) when screened against the 58 human kinases of the Eurofins KinaseProfiler Diversity panel. *S*(*x*) = number of kinases inhibited below *x*% activity when incubated with 1 μM compound/number of kinases in the panel. Compound **4** is more selective than TCMDC-135051 (**1**), with a 5-fold improved *S*(30) score and zero kinases inhibited below 20% of their original activity. (b) Human kinases inhibited ≥50% activity when exposed to 1 μM compound **4** highlighted in the human kinase phylogenetic tree. The size of the purple circle is proportional to the % inhibition. (c) Inhibition of metabolic activity of HepG2 cells. When incubated for 48 h with HepG2 cells, compound **4** (*p*EC_50_ < 4.20) proved substantially less cytotoxic than TCMDC-13501 (**1**) (*p*EC_50_ = 5.80 ± 0.14).

Compounds **2**–**4** were designed to improve the selectivity of TCMDC-135051 (**1**) by targeting a poorly conserved cysteine, Cys368. Chloroacetamide **4** was therefore screened against a representative panel of 58 human kinases from across the kinome, using Eurofins Discovery’s *KinaseProfiler* technology. Figure 9a compares the selectivity scores (*S*(50), *S*(30), and *S*(20)) of **4** and TCMDC-135051 (**1**). *S*(*x*) represents the fraction of kinases with less than *x*% remaining activity when treated with 1 μM compound. These data show compound **4** to have a significant improvement in selectivity relative to TCMDC-135051 (**1,**Table S7), with no kinases being inhibited below 20% remaining activity at 1 μM, and only 1 kinase below 30% activity (Figure 9a, Table S6). Kinases inhibited below 50% activity by compound **4** are shown on the human kinome phylogenetic tree (Figure 9b).^27^ This implies that the targeting of Cys368 can improve the selectivity for *Pf*CLK3 over the human kinome. Furthermore, using the KINOME*scan* technology (Figure S5), chloroacetamide **4** showed no substantial binding against CDK8 and CDK19, the two human kinases with equivalent cysteines (*K*_d_ > 30 μM). These data suggest that both reversible and irreversible interactions are driving the selectivity of **4**, given that kinases with equivalent nucleophiles but different ligand pockets are unable to bind this molecule.

### Chloroacetamide Covalent Inhibitor **4** Demonstrates Exquisite Selectivity Index for Parasites Over Human Cells

Cell viability experiments for compounds **4** and TCMDC-135051 (**1**) were conducted using human hepatocyte-like HepG2 cells, and both demonstrated low toxicity. HepG2 cells originate from the liver, which is particularly relevant for malaria given this is where the exoerythrocytic cycle takes place.^28^ Targeting the stage in the parasite life cycle that invades the liver is important to deliver a prophylactic treatment for malaria. Chloroacetamide **4** only fully inhibited the metabolic activity of HepG2 cells at the highest concentration assessed (300 μM) (log [I] = −3.5, Figure 9c), with solubility issues restricting exposure to higher concentrations. While an accurate *p*EC_50_ could not be calculated, these data demonstrate that chloroacetamide **4** is much less cytotoxic than TCMDC-135051 (**1**) with *p*EC_50_ = 5.8. This is hypothesized to be explained in part by the increase in kinase selectivity afforded by targeting Cys368. Compound **4** therefore demonstrates an excellent selectivity index for 3D7 parasites over human cells, potentially representing a very large therapeutic window.

### Compound **4** Demonstrates Instability in Microsomes and Hepatocytes, but is Stable in Human Serum

compound **4** was then evaluated for its stability in human serum, which demonstrated it to have a half-life of just under 13 h when incubated at 37 °C (Figure S3). This is important for blood-stage parasite experiments. Stability was also evaluated for compounds **4** and **12** in the presence of glutathione, a cellular thiol present in all body organs, most concentrated in the liver. As expected from a highly thiol reactive moiety, compound **4** had a half-life of 8 min when incubated at 37 °C at a 10:1 ratio of GSH:**4** (Figure S4). Noncovalent control compound **12** on the other hand was stable >72h. The compound was similarly labile upon incubation with human and mouse liver microsomes, as well as mouse liver hepatocytes, with half-lives of 39.5, 19.6, and 6.3 min respectively (Tables S3–5). In contrast, **compound 12** had a hepatic half-life of 165 min in the same experiment. This implies that the chloroacetamide warhead is the source of compound 4's instability. Furthermore, the difference in stability of **compound 4** in microsomes versus hepatocytes could be explained by participation in phase II metabolism in hepatocytes, which does not occur in microsomes. Phase II metabolism involves glutathione conjugation, which may contribute to **compound 4**’s poor stability. While optimization is therefore needed to improve the metabolic stability of compound **4** before *in vivo* experiments can be carried out, this molecule remains a valuable tool in validating a new mechanism in antimalarial drug discovery. More stable analogues are actively being pursued in our laboratories.

### Chloroacetamide **4** Exhibits No Propensity for Resistance After 35 Days

Resistance is a major threat to antimalarial drug design, making the propensity for resistance an essential property to evaluate. The IC_50_ of compound **4** against the Dd2-B2 parasite line was experimentally determined to be 240 nM, and the IC_90_ was determined to be 515 nM. A single-step selection was set up, using 2.3 × 10^7^ Dd2-B2 asexual blood-stage parasites in each well of a 6-well plate, at a starting concentration of 3 × IC_90_ (1,544 nM). No recrudescent parasites were observed over a 35-day selection period, indicating MIR > 1.41 × 10^8^ and log_10_MIR > 8.1.

## Discussion and Conclusions

The global effort to combat malaria has seen significant progress, yet challenges persist, particularly with emerging resistance to current frontline antimalarials and insecticides.^1^ As a result, novel chemotherapeutic agents are urgently needed to address the stagnation in infection reduction and even the resurgence observed in some regions.^2,3^ We targeted the malaria parasite protein kinase *Pf*CLK3, essential for blood stage parasite survival, as a potential avenue for therapeutic intervention.^5^ Our approach involved the identification and validation of *Pf*CLK3 as a promising target for malaria treatment, culminating in the discovery of TCMDC-135051 (**1**) as a selective inhibitor.^5,6^ In this study, we leveraged this tool compound and embarked on a drug development program aimed at generating a preclinical candidate with curative, transmission-blocking, and prophylactic properties across *Plasmodium* species.

One of the key challenges in developing next-generation antimalarials lies in ensuring efficacy across multiple stages of the parasite’s life cycle while maintaining safety, particularly for vulnerable populations, such as young children and pregnant women.^29,30^ Given the complex dynamics of parasite biology, including its 48 h erythrocytic cycle and sequestration in tissues, the development of a single-dose medicine with prolonged activity presents a formidable task. Our study addressed this challenge by exploring the potential of covalent kinase inhibitors, a strategy previously unexplored in the context of malaria. Given the success of covalent kinase inhibitors in oncology, we believe that this strategy could be harnessed in the malaria field. Though one-third of approved targeted covalent inhibitors target infectious diseases, this does not include the greatest parasitic killer.^12^ The increased duration of action attributed to an irreversible mechanism and increased selectivity can allow for smaller and less frequent dosing, which may improve patient compliance - a significant issue in the treatment of malaria.^31,32^ Acute dosing may also lead to fewer off-target effects. The ability of covalent inhibitors to evade mutation events which lead to resistance also make them ideal candidates for malaria eradication.^12,31^

Through high-resolution structural elucidation and molecular modeling, we identified a nonconserved cysteine residue (Cys368) proximal to the ATP pocket of *Pf*CLK3 as a suitable target for covalent inhibition. Subsequent synthesis and evaluation of covalent analogues revealed improved parasiticidal potency, selectivity over the human kinome, and enhanced cytotoxicity profiles compared to the parent molecule, TCMDC-135051 (**1**). Activity against multiple stages of the asexual and sexual life cycles was also demonstrated. Importantly, our findings suggest that covalent binding mechanisms offer pharmacodynamic and parasiticidal properties conducive to the development of a single-dose cure for malaria.

The specificity of our covalent inhibitor of *Pf*CLK3 over human kinases, demonstrated through kinase profiling and binding assays, underscores the potential for selective targeting of the parasite while potentially minimizing off-target effects. Moreover, the exquisite selectivity index of the lead compound, chloroacetamide **4**, for parasites over human cells highlights its promise as a therapeutic candidate with a large therapeutic window. Future research will focus on evidencing the selectivity of covalent *Pf*CLK3 inhibitors in parasites.

The covalent inhibitor maintained its potency when dosed at multiple stages of the asexual life cycle, where current frontline therapeutic artemisinin did not. This is a significant advantage of the *Pf*CLK3 inhibitors over a state of the art antimalarial, and may have implications for rate of parasite clearance. Additionally, the fact that the covalent inhibitor is active against sexual stage parasites overcomes a key obstacle in antimalarial drug discovery, and it is hoped that compound **4** has the potential to reduce transmission.

Notably, our study also provides insights into the mechanism of action underlying the prolonged parasiticidal effect of this covalent inhibitor. By comparing the activity of chloroacetamide **4** and noncovalent TCMDC-135051 (**1**) following short exposure and washout, we observed sustained potency with the covalent inhibitor, suggesting a mechanism whereby a brief exposure leads to prolonged parasite suppression. This prolonged suppression may be crucial to the resistance profile of compound **4**. No recrudescence was detected after 35 days of incubation, which is in accordance with the finding of Stokes and colleagues, whereby covalent inhibitors improved propensity for resistance in *Pf* proteasome inhibitors.^32^ These data suggest that a covalent mechanism of action may afford some protection from drug resistance.

Finally, we postulate that our findings shed light on the potential of covalent kinase inhibitors as a novel strategy for malaria treatment. To the best of our knowledge, chloroacetamide **4** represents the first covalent kinase inhibitor of malaria, as well as a rare example of a covalent inhibitor of a nonhuman kinase.^13^ By targeting essential malaria protein kinases, such as *Pf*CLK3, covalent inhibitors offer a promising avenue for the development of safe and effective antimalarials with curative and transmission-blocking properties. Further preclinical studies are warranted to validate the efficacy and safety of these compounds, with the ultimate goal of advancing toward global eradication of malaria.

## Experimental Section

### Protein Purification

A previously described full-length *Pf*CLK3 construct was expressed in *E. coli* strain C43 (DE3).^5^ Protein was purified using IMAC, TEV cleavage, and a second IMAC step before dialyzing the protein into a final buffer containing 20 mM HEPES pH 7.4, 150 mM NaCl, 1 mM TCEP and 1 mM MgCl_2_.

*Pf*CLK3 kinase domain (residues 334–699 with a C-terminal TEV cleavage sequence and His6-tag) was cloned into pFastBac vector and expressed and purified from Sf21 insect cells. Cells were infected using P2 BIICs at an MOI of 0.2 and left to express for 72 h. Harvested cells were lysed and centrifuged before purifying using IMAC and SEC in a final buffer containing 20 mM HEPES pH 7.4, 150 mM NaCl, 1 mM TCEP, and 1 mM MgCl_2_.

### Crystallization and Structure Determination

Freshly purified *Pf*CLK3 kinase domain was concentrated to 6.8 mg/mL and incubated with 0.5 mM TCMDC-135051 for 1 h before centrifuging and setting up crystal trays. Crystals grew at 4 °C in a condition containing 2 M ammonium sulfate, 0.2 M potassium sodium tartrate tetrahydrate, and 0.1 M sodium citrate pH 5.6. Crystals were cryo-protected in the reservoir solution supplemented with 10% v/v ethylene glycol and 10% v/v glycerol before flash freezing in liquid nitrogen.

Data were collected at the IMCA-CAT beamline at the APS and processed using autoPROC^33^ and STARANISO.^34^ Molecular replacement was performed using PHASER^35^ of the CCP4 program suite^36^ using the AlphaFold^37^ model of *Pf*CLK3 kinase domain as a search model. Ligand restraints were generated using grade2.^38^ Iterative rounds of model building and refinement was performed using Coot^39^ and BUSTER.^40^

### Computational Molecular Docking

All molecular docking was performed using MOE 2020.0901, using their in-house Amber10:EHT force field. Crystal structure 8RPC prepared using the “Quickprep” function in MOE. The ATP binding site was defined by “Compute” > “Sitefinder” > “Apply”. “Dummy atoms” were then created to characterize the binding site.

All ligands were drawn in ChemDraw and their 3D structure was minimized using MOE. Protomers were generated by “Compute” > “Prepare” > “Protomers”. Prepared ligands were saved to the working directory.

For covalent docking, one “Dummy atom”, created using the Site Finder tool, was moved to sit adjacent to Cys368 and dummies were used to define the binding site. The reactive site was set to “selected atoms” and the thiol of Cys368 was selected in the visualizer. The beta-mercapto carbonyl 1,4-addition reaction was selected, and “Rigid Receptor” refinement was used.The “Complex” field from the results database was then copied into MOE for each ligand. The ligands “Tag” was changed to that of the receptor in the System Manager, and the complex was minimized using the “Quick Prep” function, with “Structure Preparation” and “Protonate3D” options deselected. This minimized covalent complexes which could then be analyzed using the S score, E_conf, binding pose, and observed clash.

### Small-Molecule Synthesis and Characterization

Small molecules mentioned in this study were synthesized, with their purity and identity validated using ^1^H and ^13^C NMR, HPLC and HRMS. All tested compounds are >95% pure by HPLC Analysis. Methods and characterization of newly synthesized small molecules are supplied in the Chemical Synthesis and Characterization Data section of the Supporting Information.

#### 4-Bromo-1-tosyl-1*H*-pyrrolo[2,3-*b*]pyridine (**6**)

To a stirring solution of 4-bromo-1*H*-pyrrolo[2,3-*b*]pyridine, **5** (2.5 g, 12.7 mmol, 1.0 equiv) in anhydrous dichloromethane (40 mL) cooled in an ice-water bath to 0 °C, sodium hydride (60% in mineral oil, 1.5 g, 38.1 mmol, 3.0 equiv) was added and the mixture was stirred under nitrogen for 15 min. Toluene sulfonyl chloride (7.3 g, 38.1 mmol, 3.0 equiv) was added and the mixture was left to warm to rt while stirring under nitrogen for 18 h. The reaction mixture was slowly quenched with water and diluted with 1:1 water:DCM and two layers were separated. The aqueous layer was extracted with DCM and the combined organic layers were dried over magnesium sulfate and concentrated *in vacuo* to give a brown solid. This was purified using automated flash column chromatography eluting with 0–60% ethyl acetate:petroleum ether. The desired fractions were combined and concentrated *in vacuo* to give compound **10** as a white solid (4.4 g, 98%). R_f_: 0.55 (20% EtOAc in petroleum ether); ^1^H NMR (400 MHz, CDCl_3_) δ 8.22 (d, *J* = 5.2 Hz, 1H), 8.06 (d, *J* = 8.4 Hz, 2H), 7.78 (d, *J* = 4.0 Hz, 1H), 7.35 (d, *J* = 5.3 Hz, 1H), 7.28 (d, *J* = 8.0 Hz, 2H), 6.64 (d, *J* = 4.0 Hz, 1H), 2.37 (s, 3H); ^13^C NMR (101 MHz, CDCl_3_) δ: 146.8, 145.5, 145.0, 135.1, 129.7, 128.2, 127.0, 125.7, 124.4, 122.1, 104.9, 21.7; HRMS *m*/*z* calcd for C_14_H_12_BrN_2_O_2_S [M + H]^+^ 350.9797 found 350.9796. All other characterizations were in accordance with that of the literature.

#### 4-Bromo-2-iodo-1-tosyl-1*H*-pyrrolo[2,3-*b*]pyridine (**7**)

To a two necked flask containing **6** (2.4 g, 6.8 mmol, 1.0 equiv) in THF (80 mL) stirring at −78 °C under an argon atmosphere, lithium diisopropylamide (2 M solution in THF, 4.6 mL, 8.8 mmol, 1.3 equiv) was added. The resulting solution was then stirred at −78 °C for 90 min. Iodine (2.6 g, 9.9 mmol, 1.5 equiv) was added in one portion, and the reaction mixture was stirred at −78 °C for 60 min. The reaction was quenched with saturated ammonium chloride solution and the organic layer was washed with aqueous sodium thiosulfate and brine before drying over magnesium sulfate. The residue was then purified by column chromatography (20% ethyl acetate–hexane) to give **7** as a colorless solid (2.26 g, 70%); R_f_: 0.5 (20% EtOAc in petroleum ether); ^1^H NMR (400 MHz, CDCl_3_) δ 8.11 (d, *J* = 5.2 Hz, 1H), 8.01 (d, *J* = 8.5 Hz, 2H), 7.23 (d, *J* = 5.2 Hz, 1H), 7.22–7.19 (m, 2H), 6.96 (s, 1H), 2.30 (s, 3H); ^13^C NMR (101 MHz, CDCl_3_) δ: 149.1, 145.7, 144.7, 135.4, 129.8, 128.3, 125.3, 123.6, 122.4, 119.4, 21.7; HRMS *m*/*z* calcd for C_14_H_11_BrIN_2_O_2_S [M + H]^+^ 477.8671 found 478.8742. All other characterizations were in accordance with that of the literature.

#### 3-[4-Bromo-1-tosyl-1*H*-pyrrolo[2,3-*b*]pyridin-2-yl]-4-methoxy-benzaldehyde (**8**)

To a solution of **7** (2.2 g, 4.7 mmol, 1.0 equiv) and tetrakis(triphenylphosphine)palladium(0) (0.27 g, 0.23 mmol, 0.05 equiv) in 1,4-dioxane, 5-formyl-2-methoxyphenyl boronic acid (0.841 g, 4.7 mmol, 1.0 equiv) was added under a nitrogen atmosphere. Aqueous sodium carbonate (2 M, 16.3 mL, 33.9 mmol, 7.0 equiv) was then added and the reaction mixture left to stir at 110 °C for 18 h. Solvent was removed under vacuum and the crude product was dissolved in ethyl acetate, poured into water and extracted with ethyl acetate. The organic layer was washed with brine before drying over magnesium sulfate and purified by flash column chromatography (30% ethyl acetate–hexane) to afford **8** as a yellow foam (1.37 g, 61%); R_f_: 0.58 (50% EtOAc in petroleum ether); ^1^H NMR (400 MHz, CDCl_3_) 9.90 (s, 1H), 8.15 (d, *J* = 5.3 Hz, 1H), 7.95 (dd, *J* = 8.5, 2.1 Hz, 1H), 7.84 (d, *J* = 2.1 Hz, 1H), 7.73 (d, *J* = 8.5 Hz, 2H), 7.28 (d, *J* = 5.3 Hz, 1H), 7.13 (d, *J* = 8.5 Hz, 2H), 7.04 (d, *J* = 8.5 Hz, 1H), 6.52 (s, 1H), 3.85 (s, 3H), 2.27 (s, 3H); ^13^C NMR (101 MHz, CDCl_3_) δ: 190.4, 163.5, 148.7, 145.1, 144.8, 137.6, 135.8, 134.4, 131.4, 129.4, 129.4, 128.1, 125.1, 123.3, 123.1, 122.3, 110.6, 107.9, 56.1, 21.6; HRMS *m*/*z* calcd for C_22_H_18_BrN_2_O_4_S [M + H]^+^ 485.0165 found 485.0164. All other characterizations were in accordance with that of the literature.

#### 1-({3-[4-Bromo-1-tosyl-1*H*-pyrrolo[2,3-*b*]pyridin-2-yl]-4-methoxyphenyl}methyl)-4-*tert*-butyl-piperazine Carboxylate (**9**)

To a reaction vessel containing **8** (720 mg, 1.4 mmol, 1 equiv) in 1,2-dichloroethane (20 mL), 1-Boc-piperazine (830 mg, 4.2 mmol, 3.0 equiv) and titanium isopropoxide (0.83 mL, 2.8 mmol, 2 equiv) were added and left to stir for 5 min. Sodium triacetoxyborohydride (790 mg, 3.7 mmol, 2.5 equiv) was then added as one portion, and the reaction was left to stir for 3 h. Another portion of sodium triacetoxyborohydride (310 mg, 1.5 mmol, 1.0 equiv) was added, and the reaction was left to stir for 18 h. The reaction was then quenched by the addition of an ammonium hydroxide solution and was extracted with dichloromethane. The organic layer was washed with water and dried over magnesium sulfate. The crude residue was then purified using column chromatography (50–100% ethyl acetate–hexane) and afforded **9** as a brown oil (830 mg, 85%); R_f_: 0.24 (50% EtOAc in petroleum ether); ^1^H NMR (400 MHz, CDCl_3_) δ 8.21 (d, *J =* 5.3 Hz, 1H), 7.86 (d, *J* = 8.3 Hz, 2H), 7.40 (dd, *J* = 8.4, 2.2 Hz, 1H), 7.37–7.30 (m, 2H), 7.19 (d, *J* = 8.3 Hz, 2H), 6.93 (d, *J* = 8.4 Hz, 1H), 6.53 (s, 1H), 3.79 (s, 3H), 3.53 (d, *J* = 6.1 Hz, 2H), 3.45 (t, *J* = 4.0 Hz, 4H), 2.44 (br s, 4H), 2.35 (s, 3H), 1.46 (s, 9H). ^13^C NMR (101 MHz, CDCl_3_) δ 157.6, 154.9, 148.8, 144.9, 144.5, 139.3, 136.2, 132.0, 131.8, 129.5, 129.3, 128.2, 124.9, 123.6, 122.2, 121.6, 110.3, 107.5, 79.7, 62.4, 55.7, 53.0, 28.6, 21.7; IR (cm^–1^) 2361, 2342, 1686, 1547, 1362, 1246, 1172, 729; HRMS *m*/*z* calcd for C_31_H_36_BrN_4_O_5_S [M + H]^+^ 655.1584 found 655.1579. *Piperazine carbon peak is missing due to amide rotamers. Please see the example high temperature NMR of compound **10** (Supporting Information page S23).

#### *Tert*-butyl-4-[(4-methoxy-3-{4-[4-(methoxycarbonyl)phenyl]-1-(4-methylbenzenesulfonyl)-1*H*-pyrrolo[2,3-*b*]pyridin-2-yl}phenyl)methyl]piperazine-1-carboxylate (**10**)

To a 35 mL microwave vial containing **9** (100 mg, 0.15 mmol, 1 equiv) in 1,4-dioxane, methyl-4-(4,4,5,5-tetramethyl-1,3,2-dioxaborolan-2-yl)benzoate (44 mg, 0.17 mmol, 1.1 equiv), Pd(dppf)Cl_2_.DCM complex (7 mg, 0.009 mmol, 0.05 equiv), and sodium carbonate (1 M aq., 0.76 mL, 0.76 mmol, 5.0 equiv) were added under a nitrogen atmosphere. The solution was purged with nitrogen for 5 min and then microwaved at 110 °C for 0.5 h. The reaction was allowed to cool to room temperature, and the mixture was filtered through Celite eluting with methanol. The filtrate was evaporated and the resulting residue was purified flash chromatography (0–5% methanol in dichloromethane) to afford **10** as a brown oil (81 mg, 81%); R_f_: 0.24 (50% EtOAc in petroleum ether); ^1^H NMR (400 MHz, CDCl_3_) δ 8.49 (d, *J* = 5.0 Hz, 1H), 8.13 (d, *J* = 8.0 Hz, 2H), 7.91 (d, *J* = 8.0 Hz, 2H), 7.68 (d, *J* = 8.0 Hz, 2H), 7.39 (dd, J = 8.4, 2.2 Hz, 1H), 7.31 (d, *J* = 2.2 Hz, 1H), 7.26 (d, *J =* 8.0 Hz, 1H), 7.21 (d, *J =* 8.0 Hz, 2H), 6.93 (d, J = 8.4 Hz, 1H), 6.65 (s, 1H), 3.94 (s, 3H), 3.79 (s, 3H), 3.52 (d, J = 8.7 Hz, 2H), 3.44 (t, J = 5.1 Hz, 4H), 2.42 (s, 4H), 2.36 (s, 3H), 1.45 (s, 9H); ^13^C NMR (101 MHz, CDCl_3_) δ 166.76 (**C**O_2_CH_3_), 157.6, 155.0, 149.9, 144.7, 142.4, 140.9, 139.3, 136.5, 132.0, 131.6, 130.3, 130.3, 129.4, 129.3, 128.7, 128.3, 122.0, 120.0, 118.2, 110.3, 106.9, 79.7, 62.5, 55.7, 53.0, 52.4, 28.6, 21.8 (Ar-**C**H_3_); IR (cm^–1^) 2361, 2338, 1724, 1686, 1361, 1276, 1176, 729; HRMS *m*/*z* calcd for C_39_H_43_N_4_O_7_S [M + H]^+^ 711.2847 found 711.2856. *Piperazine carbon peak is missing due to amide rotamers. Please see the example high temperature NMR of compound **10** (Supporting Information page S23).

#### General Method for the Global Deprotection of Compound **10**

A solution of **10** (200 mg, 0.3 mmol) in 3:1 MeOH and water was treated with KOH (8 mg, 1.4 mmol, 5 equiv) and heated to reflux for 48 h. The solvent was evaporated, and the crude mixture was dissolved TFA (1 mL) and stirred at room for 2 h. The reaction mixture was then concentrated *in vacuo* to give intermediate **11**, which was taken forward without further purification.

#### 4-[2-(2-methoxy-5-{[4-(prop-2-enoyl)piperazin-1-yl]methyl}phenyl)-1*H*-pyrrolo[2,3-*b*]pyridin-4-yl]benzoic Acid (**2**)

**11** (0.02 mmol, 1.0 equiv) was dissolved in anhydrous DMF and treated with acryloyl chloride (2.4 μL, 0.03 mmol, 1.5 equiv) and triethylamine (16 μL, 0.12 mmol, 6.0 equiv) and stirred at room temperature for 2h. The reaction was quenched with water and purified by automated flash column chromatography to give **2** as a yellow solid (7.5 mg, 74% yield, 99% purity); ^1^H NMR (500 MHz, DMSO-d6) δ 12.03 (d, *J* = 2.1 Hz, 1H), 8.35 (d, *J* = 5.0 Hz, 1H), 8.14 (d, 10.0 Hz, 2H), 7.95–7.89 (m, 3H), 7.50 (dd, *J* = 8.5, 2.2 Hz, 1H), 7.31–7.25 (m, 2H), 7.08 (d, *J* = 2.1 Hz, 1H), 6.80 (dd, *J* = 16.6, 10.5 Hz, 1H), 6.17 (dd, *J* = 16.7, 2.1 Hz, 1H), 5.76 (dd, *J* = 10.4, 2.2 Hz, 1H), 4.34 (s, 2H), 3.95 (s, 3H), 3.43 (s, 4H), 3.04 (s, 4H); ^13^C NMR (126 MHz, DMSO-d6) δ 167.5, 164.8, 157.7, 150.0, 144.6, 143.7, 143.1, 139.4, 136.3, 133.1, 132.3, 131.0, 130.5, 129.0, 128.9, 127.9, 120.7, 118.5, 115.3, 112.9, 99.8, 59.0, 56.4, 51.3;* HRMS *m*/*z* calcd for C_29_H_29_N_4_O_4_ [M + H]^+^ 497.2183 found 497.2182; IR (cm^–1^) 1669, 1597, 1431, 1260, 1118, 721; HPLC *T*_R_ (min) 12.34 (5–95% ACN 0.1% TFA in H_2_O 0.1% TFA over 20 min), 21.32 (5–95% ACN 0.1% TFA in H_2_O 0.1% TFA over 50 min); M.P. (°C) 181–183 *Piperazine carbon peak is missing due to amide rotamers. Please see the example high temperature NMR of compound **10** (Supporting Information page S23).

#### 4-{2-[5-({4-[(2*E*)-4-(dimethylamino)but-2-enoyl]piperazin-1-yl}methyl)-2-methoxyphenyl]-1*H*-pyrrolo[2,3-*b*]pyridin-4-yl}benzoic Acid (**3**)

To a solution of (*E*)-4-(dimethylamino)-2-butenoic acid hydrochloride (21 mg, 0.13 mmol, 2.0 equiv) in NMP (0.35 mL, 0.36 M) at 0 °C was added thionyl chloride (SOCl_2_) (9 μL, 0.13 mmol, 2.0 equiv) and premixed for 20 min. A solution of **11** (0.06 mmol, 1.0 equiv) in NMP (0.35 mL, 0.18 M) was then added to the premixed solution and allowed to stir at room temperature for 1 h. The reaction was quenched with water and purified by automated flash column chromatography (Biotage Isolera one, 25*g* C_18_ reverse phase column, 20–40% ACN + 0.1% TFA in H_2_O + 0.1% TFA). The product was then further purified by reverse-phase HPLC (20–40% ACN + 0.1% TFA in H_2_O + 0.1% TFA) to afford compound **3** as a yellow solid (16.6 mg, 46% yield, 98% purity); ^1^H NMR (400 MHz, DMSO-d6) δ 12.06 (s, 1H), 10.26 (s, 1H,), 8.36 (d, *J* = 5.0 Hz, 1H), 8.14 (d, *J* = 8.1 Hz, 2H), 7.96 (d, *J* = 2.2 Hz, 1H), 7.93 (d, *J* = 8.0 Hz, 2H), 7.51 (dd, *J* = 8.6, 2.2 Hz, 1H), 7.29 (d, *J* = 5.0 Hz, 1H), 7.28 (d, *J* = 8.6, 1H) 7.10 (d, *J* = 2.0 Hz, 1H), 6.89 (d, *J* = 15.1 Hz, 1H), 6.68–6.58 (m, 1H), 4.35 (s, 2H), 3.96 (s, 3H), 3.87 (d, *J* = 7.0 Hz, 2H), 3.59–3.27 (m, 4H), 3.11 (m, 4H), 2.77 (s, 6H).^13^C NMR (101 MHz, DMSO-*d*_6_) δ 167.0, 163.3, 157.3, 149.2, 142.9, 142.6, 139.2, 135.9, 132.9, 132.7, 131.9, 130.6, 130.0, 128.5, 128.3, 121.4, 120.2, 118.1, 114.9, 112.4, 99.3, 58.5, 57.0, 56.0, 50.6, 50.2 42.0; HRMS *m*/*z* calcd for C_32_H_37_N_5_O_4_ [M + H]^+^ 554.2762 found 554.2758; IR (cm^–1^) 1669, 1611, 1429, 1267, 1180, 1122, 721; HPLC *T*_R_ (min) 11.69 (5–95% ACN 0.1% TFA in H_2_O 0.1% TFA over 20 min), 20.46 (5–95% ACN 0.1% TFA in H_2_O 0.1% TFA over 50 min), 98% purity M.P. (°C) 110.

#### 4-[2-(5-{[4-(2-Chloroacetyl)piperazin-1-yl]methyl}-2-methoxyphenyl)-1*H*-pyrrolo[2,3-*b*]pyridin-4-yl]benzoic Acid (**4**)

**11** (0.04 mmol, 1.0 equiv) was dissolved in anhydrous DMF and treated with chloroacetyl chloride (4 μL, 0.06 mmol, 1.5 equiv) and triethylamine (30 μL, 0.24 mmol, 6 equiv) and stirred at room temperature for 2h. The reaction was quenched with water and purified by automated flash column chromatography (Biotage Isolera one, 25*g* C_18_ column, 5–95% ACN + 0.1% TFA in H_2_O + 0.1% TFA) to give 9.3 mg of compound **4** in 45% yield, 99% purity; ^1^H NMR (400 MHz, DMSO-d6): δ 12.02 (d, *J* = 2.1 Hz, 1H), 8.35 (d, *J* = 5.0 Hz, 1H), 8.14 (d, *J =* 8.4 Hz, 2H), 7.98 (d, *J* = 2.1 Hz, 1H), 7.93 (d, *J =* 8.4, 2H), 7.52 (dd, *J* = 8.5, 2.1 Hz, 1H), 7.31–7.25 (m, 2H), 7.11 (d, *J* = 2.1 Hz, 1H), 4.45 (s, 2H), 4.34 (s, 2H), 3.96 (s, 3H), 3.43 (m, 4H), 3.09 (m, 4H); ^13^C NMR (101 MHz, DMSO-*d*_6_): δ 167.0, 164.9, 157.2, 149.4, 143.1, 142.6, 139.0, 135.8, 132.7, 131.9, 130.5, 130.0, 128.4, 121.4, 120.2, 118.0, 114.8, 112.6, 99.3, 58.5, 56.0, 50.4, 45.7, 41.8; HRMS *m*/*z* calcd for C_28_H_28_ClN_4_O_4_ [M + H]^+^ 519.1794 found 519.1792; IR (cm^–1^) 1670, 1436, 1263, 1183, 1127, 721; HPLC *T*_R_ (min) 12.61 (5–95% ACN 0.1% TFA in H_2_O 0.1% TFA over 20 min), 22.38 (5–95% ACN 0.1% TFA in H_2_O 0.1% TFA over 50 min); M.P. (°C) degraded at 178.

#### 4-(2-{5-[(4-Acetylpiperazin-1-yl)methyl]-2-methoxyphenyl}-1*H*-pyrrolo[2,3-*b*]pyridin-4-yl)benzoic Acid (**12**)

**11** (0.04 mmol, 1.0 equiv) was dissolved in anhydrous DMF and treated with acetyl chloride (4 μL, 0.06 mmol, 1.5 equiv) and triethylamine (30 μL, 0.24 mmol, 6 equiv) and stirred at room temperature for 2h. The reaction was quenched with water and purified by automated flash column chromatography (Biotage Isolera one, 25*g* C18 column (5–95% ACN + 0.1% TFA in H_2_O + 0.1% TFA) to give 9.8 mg of compound **12** as a yellow film in 45% yield, 99% purity. ^1^H NMR (400 MHz, DMSO-*d*_6_) δ 12.07 (s, 1H), 8.37 (d, *J* = 5.0 Hz, 1H), 8.15 (d, *J* = 8.3 Hz, 2H), 7.97–7.90 (m, 3H), 7.51 (dd, *J* = 8.5, 2.2 Hz, 1H), 7.32–7.26 (m, 2H), 7.10 (d, *J* = 1.9 Hz, 1H), 4.34 (s, 2H), 3.96 (s, 3H,), 2.04 (s, 3H);* ^13^C NMR (101 MHz, DMSO-*d*_6_) δ 168.6, 167.1, 157.3, 149.2, 142.9, 142.6, 139.3, 136.0, 132.8, 131.9, 130.6, 130.1, 128.5, 121.4, 120.2, 118.2, 114.9, 112.4, 99.4, 58.5, 56.0, 50.7, 50.3, 21.0; HRMS *m*/*z* calcd for C_28_H_28_N_4_O_4_ [M + H]^+^ 485.2183 found 485.2186; IR (cm^–1^) 2361, 167, 1636, 1428, 1265, 1178, 1118, 720; HPLC *T*_R_ (min) 12.18 (5–95% ACN 0.1% TFA in H_2_O 0.1% TFA over 20 min), 21.46 (5–95% ACN 0.1% TFA in H_2_O 0.1% TFA over 50 min), 99% purity; *Piperazine protons are missing due to amide rotamers, HSQC cross peaks at 3.39 ppm/50.66 and 3.04 ppm/50.34 ppm (page 25). Please also see example high temperature NMR of compound **10** (Supporting Information page S23).

### Trypsin Digest and MS Analysis of Modified Peptides

After incubation with a 5-fold excess of compound **4** for 1 h as described above, 1.2 μL DTT (final concentration 1 mM) was added to quench excess inhibitor. 2 μL of Pierce trypsin protease (1 mg/mL) was added to give a final protein:trypsin ratio of 10:1. Overnight incubation at 37 °C afforded a series of peptides, which were prepared using PierceTM C18 spin columns according to the manufacturer’s procedure.

The resulting peptide mixture was analyzed by high resolution nESI FT-ICR MS using a 12 Telsa Solarix 2XR mass spectrometer (Bruker Daltonics) equipped with a nanomate infusion robot (Advion Biosciences). The resulting mass spectra were then processed by using the SNAP algorithm in Data Analysis (Bruker Daltonics) to produce monoisotopic mass lists. The mass lists were then searched against the primary amino acid sequence of *Pf*CLK3 kinase domain_343–699_ using MS-Fit in Protein Prospector (University of California, San Francisco) and ProSight Lite v1.4 (Northwestern University). For all analyses, error tolerances of 10 ppm were used. This analysis resulted in the identification of 523 peptides, representing 59% sequence coverage.

The analysis indicated that three peptides (YSVVCELVGK, NITCDLLEHQYWLK, and YGNGHGLNATAVHCYTK) had been modified by a single neutral monoisotopic mass of 482.195405, corresponding to the covalent adduct product of compound **4** (C_28_H_26_N_4_O_4_). Of these three peptides, the relative abundance of one (YSVVCELVGK) was multiple orders of magnitude higher than the other two (6.2 × 10^8^ vs 1.0 × 10^7^ and 1.9 × 10^7^). In order to confirm the modification of this peptide, the peptide was isolated and fragmented using collision induced dissociation (CID). Fragmentation confirmed the peptide sequence and located the modification of residue Cys368.

### Time Resolved Förster Resonance Energy Transfer (TR-FRET) Assay

To a black 384-well plate was added 2.5 μL of each concentration of inhibitor serially diluted 1 in 3, 11 times from a 40 μM (4×) top concentration normalized to 4% DMSO, and 5 μL of 50 nM (2×) recombinant *Pf*CLK3. Both were dissolved in kinase buffer (50 mM HEPES 7.4, 1 mM EGTA, 1 mM MgCl_2_, 0.01% Tween, and 1 mM TCEP). After a 15 min preincubation, 2.5 μL of substrate mix (20 μM/2 mM/12 mM ATP, 200 nM MBP in kinase buffer) was added [MBP sequence: CFFKNIVTPRTPPPSQGK]. Plates were sealed and centrifuged at 1000 rpm for 1 min and incubated at 37 °C for 2 h. 5 μL of detection mix (30 mM EDTA, 3 nM AntiMBP in 1× PerkinElmer Lance detection buffer) was added to quench the kinase reaction, and plates were incubated at room temperature in the dark for 1 h. Emission of the acceptor was then read using a PHERAstar fluorescence plate reader, and the results () were normalized to the no inhibitor (positive) and no protein (negative) controls via ( to give %inhibition. Each concentration was performed in triplicate, and each experiment was repeated 3 times. All 9 enzymatic reactions were then grouped, and a nonlinear regression curve with four parameters was then plotted using GraphPad Prism, generating activity data.

### Thermal Shift

To a 384-well thermal shift plate, 5 μL protein thermal shift buffer (Thermo Fisher Scientific), 5 μg full length *Pf*CLK3 (4.17 μL, 1.2 mg/mL), 8.13 μL thermal shift buffer (Thermo Fisher Scientific), 0.2 μL 10 mM compound or vehicle (DMSO), and 2.5 μL protein thermal shift dye (Thermo Fisher Scientific) were added in triplicate. The plate was then sealed and heated from 5 to 95 °C over 15 min using QuantStudio^5^ 5 Qpcr (Thermo Fisher Scientific). Fluorescence was recorded as proteins unfolded. The Boltzmann distribution was calculated and *T*_m_ obtained using Protein Thermal Shift Software v1.4 (Thermo Fisher Scientific).

### *P. falciparum* (3D7) Culture and Synchronization

*P. falciparum* cultures were maintained in RPMI-1640 media (Invitrogen) supplemented with 0.2% sodium bicarbonate, 0.5% Albumax II, 2.0 mM l-glutamine (Sigma), and 10 mg/L gentamycin. For continuous culture, the parasites were maintained at 4% hematocrit in human erythrocytes from O+ blood donors and between 0.5 and 3% parasitemia in an incubator at 37 °C, 5% carbon dioxide (CO_2_), 5% oxygen (O_2_), and 90% nitrogen (N_2_). To obtain highly synchronous ring stage parasites for assays, cultures were double synchronized using Percoll and Sorbitol synchronization. First, highly segmented schizonts were enriched by centrifugation on a 70% Percoll (GE Healthcare) cushion gradient. The schizont pellet was collected and washed twice before fresh erythrocytes were added to a final hematocrit of 4%, and incubated for about 1–2 h shaking continuously to allow merozoites egress and reinvasion of new erythrocytes. Residual schizonts were then removed by a second Percoll purification followed by treating the ring pellet with sorbitol to generate highly synchronous 1–2 h old ring-stage parasites.

### *Ex VivoP. falciparum* (3D7*)* Inhibition Assay

To determine the IC_50_ of the molecules in parasites (*P. falciparum* 3D7) *ex vivo*, the molecules were diluted 1 in 3 from a starting concentration of 100 μM for 12 dilution points in triplicate. 50 μL of freshly diluted drugs, at twice the required final concentrations, were aliquoted into black 96-well plates. To the compound plates, 50 μL of parasites prepared at 8% hematocrit at a parasitemia of 0.3–0.5% were added and mixed by pipetting up and down several times giving a final culture volume of 100 μL at the required compound concentration (top concentration of 100 μM) and 4% hematocrit. To the “no compound” control, growth media was added and uninfected erythrocytes were included on the plate as blank. The outer wells were filled with media to reduce evaporation from the experimental wells and the plates incubated for 72 h (±2 h) to allow the parasites sufficient time to reinvade before they are collected and frozen. For cellular washout studies, compound media was exchanged for compound-free media after 6 h, and the parasites incubated for a further 66 h. To quantify growth inhibition, the plates were thawed at room temperature for at least 1 h and 100 μL of lysis buffer (20 mM Tris-HCl; 5 mM EDTA; 0.004% saponin and Triton X-100) in PBS containing Sybr green I (1 μL in 5 mL) was added to each well and mixed by pipetting up and down several times and incubated for 1 h in the dark shaking. Using a Fluoroskan/ClarioStar plate reader at excitation of 485 nm and emission of 538 nm, plate absorbances were acquired. The data was normalized against the controls and graphs were generated using Graph Pad Prism 8 to determine the IC_50_ values using the nonlinear regression log (inhibitor) versus response (three parameter) curve. All experiments were performed 3 times.

### Time of Inhibition Assays in 3D7 Parasites

Synchronised parasites between 0.4 and 0.6% parasitemia at 4% hematocrit were plated in 384 well plates. To early ring stage plate (0−) rings, serially diluted drugs were added to the parasites in triplicate and incubated as plate 1. At 16 hpi, late ring stage parasites were exposed to the same drug concentrations previously diluted as in plate 1. This was repeated for 24 hpi plates. All plates were then incubated at 37 °C until 72 hpi before the plates were removed from the incubator and frozen overnight at −20 °C. As described previously, the plates were thawed at RT for about an hour before they were SyBr green stained in lysis buffer. These data were obtained twice and each data point in triplicate.

### Gametocyte Inhibition

Synchronous asexual NF54 gametocyte producing line was grown to high parasitemia and induced to gametocytogenesis using the crash method previously described.^41,42^ For setup, starting parasitemia at 0.5–0.7% was plated in a 6-well plate at 6% hematocrit in complete media. The media in each well was changed daily until the parasites become visibly stressed before the hematocrit was lowered by increasing the volume of medium to stimulate gametocyte production (1). Once the parasitemia is very high, reinvasion of asexual parasites was blocked by treating the cultures with heparin daily for 4 days. Smears were taken daily to monitor the parasite conversion to gametocytes. At day 7 post setup, when stage I/II gametocytes appear, drug treatment was initiated at the prescribed concentrations–drug was refreshed daily until day 15 when gametocytes in untreated control reach stage V. Thin blood smears were Giemsa stained and gametocytaemia counted. This experiment was carried out 3 times

### Selectivity Assay

Compounds were evaluated using Eurofins Discovery’s KinaseProfiler Diversity Panel of 58 representative kinases. KinaseProfiler is a radiometric assay using [γ-^33^P]-ATP to measure the phosphorylation of individual kinase substrates. A representative protocol for Abl is given below. Further details can be found by visiting the Eurofins Discovery Web site.

Abl (h) is incubated with 8 mM MOPS pH 7.0, 0.2 mM EDTA, 50 μM EAIYAAPFAKKK, 10 mM Magnesium acetate, and [γ-^33^P]-ATP (specific activity and concentration as required). The reaction is initiated by the addition of the Mg/ATP mix. After incubation for 40 min at room temperature, the reaction is stopped by the addition of phosphoric acid to a concentration of 0.5%. 10 μL of the reaction is then spotted onto a P30 filtermat and washed four times for 4 min in 0.425% phosphoric acid and once in methanol prior to drying and scintillation counting.

CDK11 (CDK19) and CDK8 which have residues equivalent to C368 were then evaluated using KINOME*scan* technology. Kinase-tagged T7 phage strains were prepared in an *E. coli* host derived from the BL21 strain. *E. coli* were grown to log-phase and infected with T7 phage and incubated with shaking at 32 °C until lysis. The lysates were centrifuged and filtered to remove the cell debris. The remaining kinases were produced in HEK-293 cells and were subsequently tagged with DNA for qPCR detection. Streptavidin-coated magnetic beads were treated with biotinylated small molecule ligands for 30 min at room temperature to generate affinity resins for kinase assays. The liganded beads were blocked with excess biotin and washed with blocking buffer (SeaBlock (Pierce), 1% BSA, 0.05% Tween 20, 1 mM DTT) to remove unbound ligand and to reduce nonspecific binding. Binding reactions were assembled by combining kinases, liganded affinity beads, and test compounds in 1× binding buffer (20% SeaBlock, 0.17x PBS, 0.05% Tween 20, and 6 mM DTT). Test compounds were prepared as 111X stocks in 100% DMSO. *K*_d_ was determined using an 11-point 3-fold compound dilution series with three DMSO control points. All compounds for *K*_d_ measurements are distributed by acoustic transfer (noncontact dispensing) in 100% DMSO. The compounds were then diluted directly into the assays such that the final concentration of DMSO was 0.9%. All reactions were performed in polypropylene 384-well plate. Each was a final volume of 0.02 mL. The assay plates were incubated at room temperature with shaking for 1 h and the affinity beads were washed with wash buffer (1× PBS, 0.05% Tween 20). The beads were then resuspended in elution buffer (1× PBS, 0.05% Tween 20, 0.5 μM nonbiotinylated affinity ligand) and incubated at room temperature with shaking for 30 min. The kinase concentration in the eluates was measured by qPCR.

### Human Cell Viability Assay

Mycoplasma tested HepG2 cells were cultured in Dulbecco’s modified Eagle medium (DMEM) with 10% fetal bovine serum, 1% nonessential AA, 1% sodium pyruvate, 1% pen–strep and 100 μg/mL normocin. Cultures were incubated at 37 °C and 5% CO_2_. Cells were detached using 0.05% trypsin–EDTA. 500 nL of drug compound dilutions, in triplicate, were added to 384-well, black, clear bottom assay plates using a mosquito liquid handling machine. Assay plates, containing compound dilutions, were seeded at 5,000 cells/well and incubated for 48 h. 40 μM final concentration resazurin, diluted in DPBS, was added to the assay plates which were then incubated for 4 h and analyzed using a ClariostarTM plate reader to measure fluorescence Intensity (545–20 nm/600–40 nm). Three control compounds were included on every assay plate—Tamoxifen, puromycin, and TCMDC-135051. Maximum signal control was obtained from wells with DMSO only and minimum signal control with Tamoxifen 100 μM. These were used to normalize data and give percentage inhibition of metabolic activity. Experiments were performed *N* = 3 and normalized data was grouped and a nonlinear regression curve with four parameters was plotted using GraphPad Prism, generating activity data.

### Parasite Culture for Resistance Studies (Dd2-B2)

*Plasmodium falciparum* asexual blood stage (ABS) parasites were cultured at 2% hematocrit (HCT) in human O+ RBCs in RPMI-1640 media, supplemented with 25 mM HEPES, 50 mg/L hypoxanthine, 2 mM l-glutamine, 0.21% sodium bicarbonate, 0.5% (wt/vol) AlbuMAXII (Invitrogen) and 10 μg/mL gentamycin, in modular incubator chambers (Billups-Rothenberg) at 5% O_2_, 5% CO_2_ and 90% N_2_ at 37 °C. Dd2 parasites were obtained from T. Wellems (NIAID, NIH). Dd2-B2 is a genetically homogeneous line that was cloned from Dd2 by limiting dilution in the Fidock lab.

### Drug Susceptibility Assays (Dd2-B2)

To define the IC_50_ of ABS parasites, Dd2-B2 ring-stage cultures at 0.3% parasitemia and 1% hematocrit for 72 h were exposed to a range of ten drug concentrations that were 2-fold serially diluted in duplicates along with drug-free controls. All *in vitro* studies were done such that the final DMSO concentration was <0.5%. Parasite survival was assessed by flow cytometry on an iQue flow cytometer (Sartorius) using SYBR green and MitoTracker deep red FM (Life Technologies) as nuclear stain and vital dyes, respectively.

### Resistance Studies

The IC_50_ for compound **4** was experimentally determined to be 239.5 nM (N,*n* = 4,2), and the IC_90_ was determined to be 514.7 nM (N,*n* = 4,2) in the *P. falciparum* Dd2-B2 clone. One MIR selection was set up using 2.3E7 Dd2-B2 parasites in 6 wells for a total of 1.4E8 parasites at a starting concentration of 3 × IC_90_ (1,544 nM). Each well had 6 mL of culture with an initial parasitemia of 1% at 3% HCT. Parasite clearance was observed by day 7. The selection had a consistent drug pressure of 3 × IC_90_ (1,544 nM) for 35 days, and cultures were screened three times weekly by flow cytometry and smearing. Wells are considered positive for recrudescence when the overall parasitemia reaches 0.3% and parasites are seen on a blood smear. No recrudescence was observed over the course of this selection (MIR > 1.4 x10^8^, log_10_ MIR > 8.1).

## Supporting Information Available

Molecular formula strings (CSV)
*Pf*CLK3 Crystal structure in complex with TCMDC-135051 PDB ID: 8RPC (PDB)
*Pf*CLK3 homology model with TCMDC-135051 docked (PDB)
*Pf*CLK3 homology model with **4** docked (PDB)
*Pf*CLK3 homology model with **12** docked (PDB)
Crystallization and structure determination; experimental procedures and characterization data for all compounds; thermal shift data; parasiticidal activity of compound 12; metabolic stability; selectivity data from KinaseProfiler and KINOME*scan* assays; ligand interaction map for 8RPC; copies of ^1^H, ^13^C, and NMR spectra for all compounds; and analytical HPLC traces for final compounds (PDF)

## Supplementary Material

jm4c01300_si_001.csv

jm4c01300_si_002.pdb

jm4c01300_si_003.pdb

jm4c01300_si_004.pdb

jm4c01300_si_005.pdb

## Abbreviations

ATP: adenosine triphosphate
CDK: cyclin-dependent kinase
CID: collision-induced dissociation
DMSO: dimethysulfoxide
ESI-TOF: electrospray ionization time-of-flight
MOE: molecular operating environment
*Pf*CLK3: *Plasmodium falciparum* cyclin-dependent like kinase
TCMDC: Tres Cantos antimalarial set
TR-FRET: time-resolved fluorescence energy transfer
