# Supplementary material for: Targeting PfCLK3 with Covalent Inhibitors: A Novel Strategy for Malaria Treatment
Source: J Med Chem. 2024 Oct 23;67(21):18895–910. doi: 10.1021/acs.jmedchem.4c01300 (PMC11571108; doi:10.1021/acs.jmedchem.4c01300)

Supporting Information

Targeting *Pf*CLK3 with Covalent Inhibitors: A Novel Strategy for Malaria Treatment

Skye B. Brettell,[†] Omar Janha,[‡] Abbey Begen,[§] Gillian Cann,[§] Saumya Sharma,[§] Niniola Olaniyan,[‡] Tamas Yelland,[‖] Alison J. Hole,[‖] Benazir Alam,[‖] Emily Mayville,[□] Ross Gillespie,[†] Michael Capper,[†] David A. Fidock,[□] Graeme Milligan,[‡] David J. Clarke,[¥] Andrew B. Tobin,[‡*] and Andrew G. Jamieson[†*]

[†]School of Chemistry, The Advanced Research Centre, University of Glasgow, 11 Chapel Lane, G11 6EW, U.K.
[‡]Centre for Translational Pharmacology, The Advanced Research Centre, University of Glasgow, 11 Chapel Lane, G11 6EW, U.K.
[§]KelticPharma Therapeutics, The Advanced Research Centre, University of Glasgow, 11 Chapel Lane, G11 6EW, U.K.
[‖]Evotec(UK) Ltd, 95 Park Drive, Milton Park, Abingdon, Oxfordshire, OX14 4RY, U.K.
[□]Department of Microbiology& Immunology and Center for Malaria Therapeutics and Antimicrobial Resistance, Division of Infectious Diseases, Department of Medicine, Columbia University Medical Center, New York, NY, 10032, USA
[¥]EaSTCHEM School of Chemistry, University of Edinburgh, Joseph Black Building, David, Brewster Road, Edinburgh, EH9 3FJ, U.K.
*e-mail: andrew.tobin@glasgow.ac.uk; andrew.jamieson.2@glasgow.ac.uk

Contents

General Information S2
Crystallisation and structure determination S2
Chemical Synthesis and Characterisation Data S4
Thermal Shift S10
Parasiticidal data for compound 12 .... S11
Metabolic Stability S11
Selectivity S16
NMR Spectra for Novel Compounds S21

HPLC Traces and Purity for Final Compounds S28

General Information

Chemicals and solvents were purchased from standard suppliers and used without additional purification. All glassware was stored at 120 ˚C and cooled at reduced pressure in a desiccator. Anhydrous solvents (THF, DCM) were obtained by passage through solvent filtration systems (Pure Solv) and solvents were transferred by syringe. All reactions carried out under inert or dry atmosphere were carried out under a blanket of nitrogen. Thin layer chromatography (TLC) was performed using aluminium plates precoated with silica gel (0.25 mm, 60 Å pore-size) impregnated with a fluorescent indicator (254 nm). Visualization on TLC was achieved by the use of UV light (254 nm). Flash column chromatography was performed using a Biotage Isolera One Flash Chromatography instrument. Proton nuclear magnetic resonance spectra (^{1}H NMR) were recorded on AVANCE III 400 Bruker (400 MHz). Proton chemical shifts are expressed in parts per million (ppm, δ scale) and are referenced to residual protium in the NMR solvent ($CDCl_3$, δ 7.26; CD_3OD, δ 3.31 and DMSO-d6, δ 2.50). The following abbreviations were used to describe peak patterns when appropriate: br = broad, s = singlet, d = doublet, t = triplet, q = quartet, sept = septet, m = multiplet. Coupling constants, J, were reported in Hertz unit (Hz). Carbon 13 nuclear magnetic resonance spectroscopy (^{13}C NMR) was recorded on AVANCE III 400 Bruker (101 MHz) and was fully decoupled by broad band decoupling. Chemical shifts were reported in ppm referenced to the centre line of a triplet at 77.16, 49.0, 39.5 ppm of $CDCl_3$, CD_3OD and DMSO-d6. 2-dimensional NMR (COSY, HMBC, HSQC) was used to assign NMR spectra. High-resolution mass spectrometry (HRMS) was performed on a Bruker microTOF-Q II (ESI+). Infra-Red spectroscopy was recorded using a Shimadzu FTIR-8400S.

Crystallisation and structure determination

	*Pf*CLK3: TCMDC-135051*
**Data collection**	
PDB code	8RPC
Space group	C2221
Cell dimensions	
a, *b*, *c* (Å)	69.63, 128.80, 108.22
α, β, γ (°)	90.00, 90.00, 90.00
Resolution (Å)	64.40 – 2.10 (2.26 – 2.10)
R_{merge}[a]	0.15 (0.60)
$I / \alpha I$	6.9 (1.5)
Completeness (%) - ellipsoidal	93 (66)
Redundancy	6.2 (2.9)
**Refinement**	
Resolution (Å)	64.40 – 2.10
No. reflections	136944
R_{work}[b] / R_{free}[c]	0.20/0.26
No. atoms	
Protein	2890
Ligand/ion	80
Water	196
B-factors	
Protein	36.5
Ligand/ion	42.2
Water	42.2
R.m.s. deviations	
Bond lengths (Å)	0.012
Bond angles (°)	1.54
Ramachandran statistics	
Favoured region (%)	96.6
Allowed region (%)	3.1
Outlier region (%)	0.3

***Table S1**: Data collection and refinement statistics*

**Data collected from a single crystal*

$^{a}R_{merge} = \sum|I_{obs}-I_{avg}|/\sum I_{avg}$

$^{b}R_{work} = \sum|F_{obs}-F_{calc}|/\sum F_{obs}$

[c]Five percent of the reflection data was selected at random as a test set, and only this data was used to calculate R_{free}.

Values for the highest resolution bin are in parentheses

Chemical Synthesis and Characterisation Data

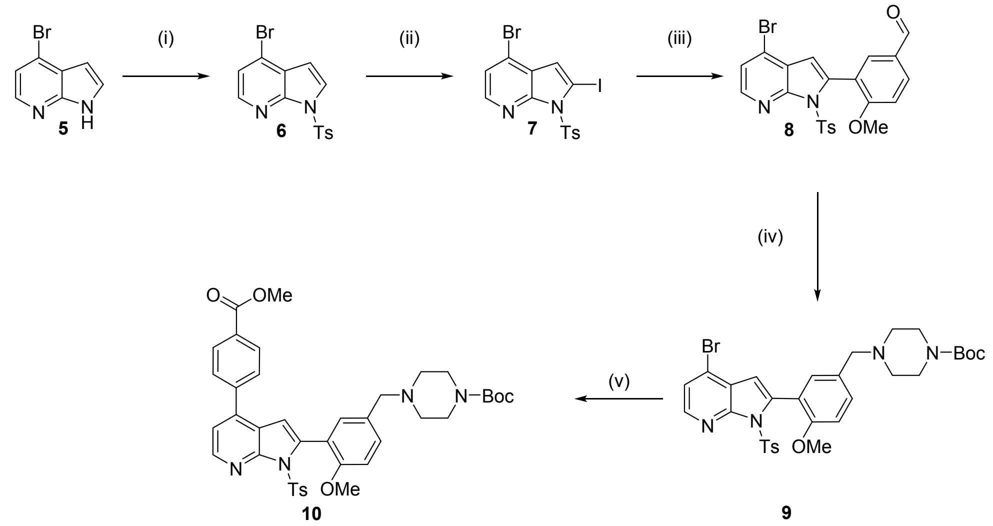

Scheme S1: Synthesis of key intermediate **10** (i) TsCl, NaH, DCM, 0 ℃ - rt, 1h; (ii) LDA, I_2, THF, -78 ℃, 2.5 h; (iii) (5-formyl-2-methoxyphenyl)boronic acid, $Pd(PPh_3)_4$, Na_2CO_3, 1,4-dioxane, 110 °C, 18 h; (iv) N-Boc-piperazine, $NaBH(AcO)_3$, dichloroethane, rt, 18 h; (v) 4-(4,4,5,5-tetramethyl-1,3,2-dioxaborolan-2-yl)benzoic acid, $Pd(dppf)Cl_2 \cdot CH_2Cl_2$, Na_2CO_3, 1,4-dioxane, 110 °C, 0.5 h, MW.

<u>4-Bromo-1-tosyl-1H-pyrrolo[2,3-b]pyridine (6)</u>

To a stirring solution of 4-bromo-1H-pyrrolo[2,3-b]pyridine, 9 (2.5 g, 12.7 mmol, 1.0 eq) in anhydrous dichloromethane (40 mL) cooled in an ice-water bath to 0 °C was added sodium hydride (60% in mineral oil, 1.5 g, 38.1 mmol, 3.0 eq) and the mixture was stirred under nitrogen for 15 min. Toluene sulphonyl chloride (7.3 g, 38.1 mmol, 3.0 eq) was added and the mixture was left to warm to rt whilst stirring under nitrogen for 18 h. The reaction mixture was slowly quenched with water and diluted with 1:1 water:DCM and two layers were separated.

The aqueous layer was extracted with DCM and the combined organic layers were dried over magnesium sulphate and concentrated in vacuo to give a brown solid. This was purified using automated flash column chromatography eluting with 0-60% ethyl acetate:petroleum ether. The desired fractions were combined and concentrated in vacuo to give compound 10 as a white solid (4.4 g, 98%). **R_f**: 0.55 (20% EtOAc in petroleum ether); **^{1}H NMR** (400 MHz, $CDCl_3$) δ 8.22 (d, *J* = 5.2 Hz, 1H), 8.06 (d, *J* = 8.4 Hz, 2H), 7.78 (d, *J* = 4.0 Hz, 1H), 7.35 (d, *J* = 5.3 Hz, 1H), 7.28 (d, *J* = 8.0 Hz, 2H), 6.64 (d, *J* = 4.0 Hz, 1H), 2.37 (s, 3H); **^{13}C NMR** (101 MHz, $CDCl_3$) δ: 146.8, 145.5, 145.0, 135.1, 129.7, 128.2, 127.0, 125.7, 124.4, 122.1, 104.9, 21.7; **HRMS** *m/z* calcd for $C_{14}H_{12}BrN_2O_2S$ $[M+H]^+$ 350.9797 found 350.9796. All other characterisation was in accordance with that of the literature.

4-Bromo-2-iodo-1-tosyl-1H-pyrrolo[2,3-b]pyridine (7)

To a two necked flask containing **6** (2.4 g, 6.8 mmol, 1.0 equiv.) in THF (80 mL) stirring at -78˚C under argon atmosphere was added Lithium diisopropylamide (2 M solution in THF, 4.6 mL, 8.8 mmol, 1.3 equiv.). The resulting solution was then stirred at -78 ºC for 90 mins. Iodine (2.6 g, 9.9 mmol, 1.5 equiv.) was added in one portion, and the reaction mixture was stirred at -78 °C for 60 mins. The reaction was quenched with saturated ammonium chloride solution and the organic layer was washed with aqueous sodium thiosulphate and brine before drying over magnesium sulphate. The residue was then purified by column chromatography (20% ethyl acetate-hexane) to give **7** as a colourless solid (2.26 g, 70%); **R_f**: 0.5 (20% EtOAc in petroleum ether); **^{1}H NMR** (400 MHz, $CDCl_3$) δ 8.11 (d, *J* = 5.2 Hz, 1H), 8.01 (d, *J* = 8.5 Hz, 2H), 7.23 (d, *J* = 5.2 Hz, 1H), 7.22-7.19 (m, 2H), 6.96 (s, 1H), 2.30 (s, 3H); **^{13}C NMR** (101 MHz, $CDCl_3$) δ: 149.1, 145.7, 144.7, 135.4, 129.8, 128.3, 125.3, 123.6, 122.4, 119.4, 21.7; ; **HRMS** *m/z* calcd for $C_{14}H_{11}BrIN_2O_2S$ $[M+H]^+$ 477.8671 found 478.8742. All other characterisation was in accordance with that of the literature.

3-[4-Bromo-1-tosyl-1H-pyrrolo[2,3-b]pyridin-2-yl]-4-methoxy-benzaldehyde (8)

To a solution of **7** (2.2g, 4.7 mmol, 1.0 equiv.) and tetrakis(triphenylphosphine)palladium(0) (0.27 g, 0.23 mmol, 0.05 equiv.) in 1,4-dioxane was added 5-formyl-2-methoxyphenyl boronic acid (0.841 g, 4.7 mmol, 1.0 equiv.) under a nitrogen atmosphere. Aqueous sodium carbonate (2 M, 16.3 mL, 33.9 mmol, 7.0 equiv.) was then added and the reaction mixture left to stir at 110 ºC for 18 hrs. Solvent was removed under vacuum and the crude was dissolved in ethyl acetate, poured into water and extracted with ethyl acetate. The organic layer was washed with brine before drying over magnesium sulphate and purified by flash column chromatography

(30% ethyl acetate-hexane) to afford **8** as a yellow foam (1.37 g, 61%); **R_f**: 0.58 (50% EtOAc in petroleum ether); **^{1}H NMR** (400 MHz, $CDCl_3$) 9.90 (s, 1H), 8.15 (d, *J* = 5.3 Hz, 1H), 7.95 (dd, *J* = 8.5, 2.1 Hz, 1H), 7.84 (d, *J* = 2.1 Hz, 1H), 7.73 (d, *J* = 8.5 Hz, 2H), 7.28 (d, *J* = 5.3 Hz, 1H), 7.13 (d, *J* = 8.5 Hz, 2H), 7.04 (d, *J* = 8.5 Hz, 1H), 6.52 (s, 1H), 3.85 (s, 3H), 2.27 (s, 3H); **^{13}C NMR** (101 MHz, $CDCl_3$) δ: 190.4, 163.5, 148.7, 145.1, 144.8, 137.6, 135.8, 134.4, 131.4, 129.4, 129.4, 128.1, 125.1, 123.3, 123.1, 122.3, 110.6, 107.9, 56.1, 21.6 ; **HRMS** *m/z* calcd for $C_{22}H_{18}BrN_2O_4S$ $[M+H]^+$ 485.0165 found 485.0164. All other characterisation was in accordance with that of the literature.

<u>1-({3-[4-Bromo-1-tosyl-1H-pyrrolo[2,3-b]pyridin-2-yl]-4-methoxyphenyl}methyl)-4-tert-butyl piperazine carboxylate (9)</u>

To a reaction vessel containing **8** (720 mg, 1.4 mmol, 1 equiv.) in 1,2-dichloroethane (20 mL) was added 1-Boc-piperazine (830 mg, 4.2 mmol, 3.0 equiv.) and titanium isopropoxide (0.83 mL, 2.8 mmol, 2 equiv.) and left to stir for 5 minutes. Sodium triacetoxyborohydride (790 mg, 3.7 mmol, 2.5 equiv.) was then added as one portion and the reaction left to stir for 3 hours. Another portion of sodium triacetoxyborohydride (310 mg, 1.5 mmol, 1.0 equiv.) was added, and the reaction was left to stir for 18 h. The reaction was then quenched by the addition of ammonium hydroxide solution and extracted with dichloromethane. The organic layer was washed with water and dried over magnesium sulphate. The crude residue was then purified using column chromatography (50-100% ethyl acetate - hexane) and afforded **9** as a brown oil (830 mg, 85%); **R_f**: 0.24 (50% EtOAc in petroleum ether); **^{1}H NMR** (400 MHz, $CDCl_3$) δ 8.21 (d, *J* = 5.3 Hz, 1H), 7.86 (d, *J* = 8.3 Hz, 2H), 7.40 (dd, *J* = 8.4, 2.2 Hz, 1H), 7.37 – 7.30 (m, 2H), 7.19 (d, *J* = 8.3 Hz, 2H), 6.93 (d, *J* = 8.4 Hz, 1H), 6.53 (s, 1H), 3.79 (s, 3H), 3.53 (d, *J* = 6.1 Hz, 2H), 3.45 (t, *J* = 4.0 Hz, 4H), 2.44 (br s, 4H), 2.35 (s, 3H), 1.46 (s, 9H). **^{13}C NMR** (101 MHz, $CDCl_3$) δ 157.6, 154.9, 148.8, 144.9, 144.5, 139.3, 136.2, 132.0, 131.8, 129.5, 129.3, 128.2, 124.9, 123.6, 122.2, 121.6, 110.3, 107.5, 79.7, 62.4, 55.7, 53.0, 28.6, 21.7; **IR** (cm^{-1}) 2361, 2342, 1686, 1547, 1362 , 1246, 1172, 729; **HRMS** *m/z* calcd for $C_{31}H_{36}BrN_4O_5S$ $[M+H]^+$ 655.1584 found 655.1579. *Piperazine carbon peak missing due to amide rotamers. Please see example high temperature NMR of compound **10** (page S23).

<u>tert-Butyl 4-[(4-methoxy-3-{4-[4-(methoxycarbonyl)phenyl]-1-(4-methylbenzenesulfonyl)-1H-pyrrolo[2,3-b]pyridin-2-yl}phenyl)methyl]piperazine-1-carboxylate (10)</u>

To a 35 mL microwave vial containing **9** (100 mg, 0.15 mmol, 1 equiv.) in 1,4-dioxane was added methyl 4-(4,4,5,5-tetramethyl-1,3,2-dioxaborolan-2-yl)benzoate (44 mg, 0.17 mmol, 1.1 equiv.), Pd(dppf)Cl_2.DCM complex (7 mg, 0.009 mmol, 0.05 equiv.) and sodium carbonate (1M aq., 0.76 mL, 0.76 mmol, 5.0 equiv.) under a nitrogen atmosphere. The solution was purged with nitrogen for 5 mins and then microwaved at 110 ºC for 0.5 h. The reaction was allowed to cool to room temperature and the mixture was filtered through celite eluting with methanol. The filtrate was evaporated and the resulting residue was purified flash chromatography (0-5% methanol in dichloromethane) to afford **10** as a brown oil (81 mg, 81 %); **R_f**: 0.24 (50% EtOAc in petroleum ether); **^{1}H NMR** (400 MHz, $CDCl_3$) δ 8.49 (d, *J* = 5.0 Hz, 1H), 8.13 (d, *J* = 8.0 Hz, 2H), 7.91 (d, *J* = 8.0 Hz, 2H), 7.68 (d, *J* = 8.0 Hz, 2H), 7.39 (dd, J = 8.4, 2.2 Hz, 1H), 7.31 (d, *J* = 2.2 Hz, 1H), 7.26 (d, *J* = 8.0 Hz, 1H), 7.21 (d, *J*= 8.0 Hz, 2H), 6.93 (d, J = 8.4 Hz, 1H), 6.65 (s, 1H), 3.94 (s, 3H), 3.79 (s, 3H), 3.52 (d, J = 8.7 Hz, 2H), 3.44 (t, J = 5.1 Hz, 4H), 2.42 (s, 4H), 2.36 (s, 3H), 1.45 (s, 9H).; **^{13}C NMR** (101 MHz, $CDCl_3$) δ 166.76 (**CO_2CH_3**), 157.6, 155.0, 149.9, 144.7, 142.4, 140.9, 139.3, 136.5, 132.0, 131.6, 130.3, 130.3, 129.4, 129.3, 128.7, 128.3, 122.0, 120.0, 118.2, 110.3, 106.9, 79.7, 62.5, 55.7, 53.0, 52.4, 28.6, 21.8 (Ar-**CH_3**); **IR** (cm^{-1}) 2361, 2338, 1724, 1686, 1361, 1276, 1176, 729; **HRMS** *m/z* calcd for $C_{39}H_{43}N_4O_7S$ $[M+H]^+$ 711.2847 found 711.2856. *Piperazine carbon peak missing due to amide rotamers. Please see example high temperature NMR of compound **10** (page S23).

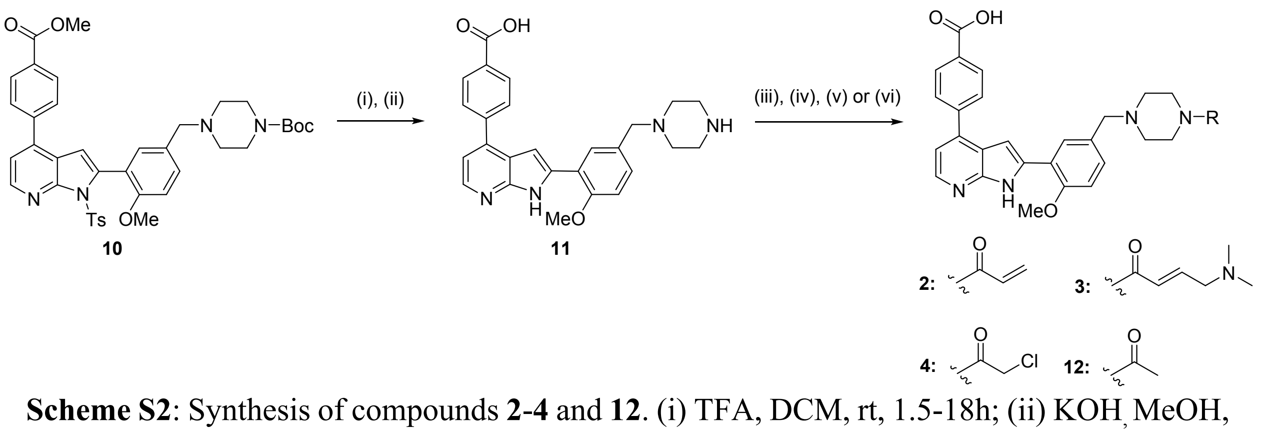

Scheme S2: Synthesis of compounds **2-4** and **12**. (i) TFA, DCM, rt, 1.5-18h; (ii) KOH, MeOH, H_2O reflux; (iii) acryloyl chloride, NEt_3, DMF, rt, 1h); (iv) (E)-4-(dimethylamino)-2-butenoic acid hydrochloride, $SOCl_2$, NMP, rt, 1.3h; (v) chloroacetyl chloride, NEt_3, DMF, rt, 2h; (vi) acetyl chloride, NEt_3, DMF, rt, 2h.

General method for the global deprotection of compound 10

A solution of **10** (200 mg, 0.3 mmol) in 3:1 MeOH and water was treated with KOH (8 mg, 1.4 mmol, 5 equiv.) and heated to reflux for 48 hours. The solvent was evaporated, and the crude mixture was dissolved TFA (1 mL) and stirred at room for 2 hours. The reaction mixture was then concentrated *in vacuo* to give intermediate **11**, which was taken forward without further purification.

4-[2-(2-Methoxy-5-{[4-(prop-2-enoyl)piperazin-1-yl]methyl}phenyl)-1H-pyrrolo[2,3-b]pyridin-4-yl]benzoic acid (2)

11 (0.02 mmol, 1.0 equiv.) was dissolved in anhydrous DMF and treated with acryloyl chloride (2.4 µL, 0.03 mmol, 1.5 equiv.) and triethylamine (16 µL, 0.12 mmol, 6.0 equiv.) and stirred at room temperature for 2h. The reaction was quenched with water and purified by automated flash column chromatography to give **2** as a yellow solid (7.5 mg, 74% yield, 99% purity); **^{1}H NMR** (500 MHz, DMSO-d6) δ 12.03 (d, J = 2.1 Hz, 1H), 8.35 (d, J = 5.0 Hz, 1H), 8.14 (d, 10.0 Hz, 2H), 7.95 – 7.89 (m, 3H), 7.50 (dd, J = 8.5, 2.2 Hz, 1H), 7.31 – 7.25 (m, 2H), 7.08 (d, J = 2.1 Hz, 1H), 6.80 (dd, J = 16.6, 10.5 Hz, 1H), 6.17 (dd, J = 16.7, 2.1 Hz, 1H), 5.76 (dd, J = 10.4, 2.2 Hz, 1H), 4.34 (s, 2H), 3.95 (s, 3H), 3.43 (s, 4H), 3.04 (s, 4H); **^{13}C NMR** (126 MHz, DMSO-d6) δ 167.5, 164.8, 157.7, 150.0, 144.6, 143.7, 143.1, 139.4, 136.3, 133.1, 132.3, 131.0, 130.5, 129.0, 128.9, 127.9, 120.7, 118.5, 115.3, 112.9, 99.8, 59.0, 56.4, 51.3;* **HRMS** *m/z* calcd for $C_{29}H_{29}N_4O_4$ $[M+H]^+$ 497.2183 found 497.2182; **IR** (cm^{-1}) 1669, 1597, 1431, 1260, 1118, 721; **HPLC** T_R (min) 12.34 (5-95% ACN 0.1% TFA in H_2O 0.1% TFA over 20 minutes), 21.32 (5-95% ACN 0.1% TFA in H_2O 0.1% TFA over 50 minutes); **M.p.** (℃) 181-183
*Piperazine carbon peak missing due to amide rotamers. Please see example high temperature NMR of compound **10** (page S23).

4-{2-[5-({4-[(2E)-4-(dimethylamino)but-2-enoyl]piperazin-1-yl}methyl)-2-methoxyphenyl]-1H-pyrrolo[2,3-b]pyridin-4-yl}benzoic acid (3)

To a solution of (*E*)-4-(dimethylamino)-2-butenoic acid hydrochloride (21 mg, 0.13 mmol, 2.0 equiv.) in NMP (0.35 mL, 0.36 M) at 0 ℃ was added thionyl chloride ($SOCl_2$) (9 µL, 0.13 mmol, 2.0 equiv.) and premixed for 20 minutes. A solution of **11** (0.06 mmol, 1.0 equiv.) in NMP (0.35 mL, 0.18 M) was then added to the premixed solution and allowed to stir at room temperature for one hour. The reaction was quenched with water and and purified by automated flash column chromatography (Biotage Isolera one, 25g C18 reverse phase column, 20-40%

ACN +0.1% TFA in H_2O + 0.1% TFA). The product was then further purified by reverse-phase HPLC (20-40% ACN +0.1% TFA in H_2O + 0.1% TFA) to afford compound **3** as a yellow solid (16.6 mg, 46% yield, 98% purity); **^{1}H NMR** (400 MHz, DMSO-d6) δ 12.06 (s, 1H), 10.26 (s, 1H,), 8.36 (d, *J* = 5.0 Hz, 1H), 8.14 (d, *J* = 8.1 Hz, 2H), 7.96 (d, *J* = 2.2 Hz, 1H), 7.93 (d, *J* = 8.0 Hz, 2H), 7.51 (dd, *J* = 8.6, 2.2 Hz, 1H), 7.29 (d, *J* = 5.0 Hz, 1H), 7.28 (d, *J* = 8.6, 1H) 7.10 (d, *J* = 2.0 Hz, 1H), 6.89 (d, *J* = 15.1 Hz, 1H), 6.68- 6.58 (m, 1H), 4.35 (s, 2H), 3.96 (s, 3H), 3.87 (d, *J* = 7.0 Hz, 2H), 3.59 – 3.27 (m, 4H), 3.11 (m, 4H), 2.77 (s, 6H**). ^{13}C NMR (101 MHz, DMSO-d6)** δ 167.0, 163.3, 157.3, 149.2, 142.9, 142.6, 139.2, 135.9, 132.9, 132.7, 131.9, 130.6, 130.0, 128.5, 128.3, 121.4, 120.2, 118.1, 114.9, 112.4, 99.3, 58.5, 57.0, 56.0, 50.6, 50.2 42.0; **HRMS** *m/z* calcd for $C_{32}H_{37}N_5O_4$ $[M+H]^+$ 554.2762 found 554.2758; **IR** (cm^{-1}) 1669, 1611, 1429, 1267, 1180, 1122, 721; **HPLC** T_R (min) 11.69 (5-95% ACN 0.1% TFA in H_2O 0.1% TFA over 20 minutes), 20.46 (5-95% ACN 0.1% TFA in H_2O 0.1% TFA over 50 minutes), 98% purity **M.p. (°C)** 110.

4-[2-(5-{[4-(2-chloroacetyl)piperazin-1-yl]methyl}-2-methoxyphenyl)-1H-pyrrolo[2,3-b] pyridin-4-yl]benzoic acid (4)

11 (0.04 mmol, 1.0 equiv.) was dissolved in anhydrous DMF and treated with chloroacetyl chloride (4 µL, 0.06 mmol, 1.5 equiv.) and triethylamine (30 µL, 0.24 mmol, 6 equiv.) and stirred at room temperature for 2h. The reaction was quenched with water and purified by automated flash column chromatography (Biotage Isolera one, 25g C18 column, 5-95% ACN +0.1% TFA in H_2O + 0.1% TFA) to give 9.3 mg of compound **4** in 45% yield, 99% purity; **^{1}H NMR** (400 MHz, DMSO-d6): δ 12.02 (d, *J* = 2.1 Hz, 1H), 8.35 (d, *J* = 5.0 Hz, 1H), 8.14 (d, *J* = 8.4 Hz, 2H), 7.98 (d, *J* = 2.1 Hz, 1H), 7.93 (d, *J* = 8.4, 2H), 7.52 (dd, *J* = 8.5, 2.1 Hz, 1H), 7.31 – 7.25 (m, 2H), 7.11 (d, *J* = 2.1 Hz, 1H), 4.45 (s, 2H), 4.34 (s, 2H), 3.96 (s, 3H), 3.43 (m, 4H), 3.09 (m, 4H); **^{13}C NMR (101 MHz, DMSO-d6):** δ 167.0, 164.9, 157.2, 149.4, 143.1, 142.6, 139.0, 135.8, 132.7, 131.9, 130.5, 130.0, 128.4, 121.4, 120.2, 118.0, 114.8, 112.6, 99.3, 58.5, 56.0, 50.4, 45.7, 41.8; **HRMS** *m/z* calcd for $C_{28}H_{28}ClN_4O_4$ $[M+H]^+$ 519.1794 found 519.1792; **IR** (cm^{-1}) 1670, 1436, 1263, 1183, 1127, 721; **HPLC** T_R (min) 12.61 (5-95% ACN 0.1% TFA in H_2O 0.1% TFA over 20 minutes), 22.38 (5-95% ACN 0.1% TFA in H_2O 0.1% TFA over 50 minutes); **M.p.** (°C) degraded at 178.

4-(2-{5-[(4-acetylpiperazin-1-yl)methyl]-2-methoxyphenyl}-1H-pyrrolo[2,3-b]pyridin-4-yl)benzoic acid (12)

11 (0.04 mmol, 1.0 equiv) was dissolved in anhydrous DMF and treated with acetyl chloride (4µL, 0.06 mmol, 1.5 equiv.) and triethylamine (30 µL, 0.24 mmol, 6 equiv.) and stirred at room temperature for 2h. The reaction was quenched with water and purified by automated flash column chromatography (Biotage Isolera one, 25g C18 column (5-95% ACN +0.1% TFA in H_2O + 0.1% TFA) to give 9.8 mg of compound **12** as a yellow film in 45% yield, 99% purity. **^{1}H NMR (400 MHz, DMSO-d6)** δ 12.07 (s, 1H), 8.37 (d, *J* = 5.0 Hz, 1H), 8.15 (d, *J* = 8.3 Hz, 2H), 7.97 – 7.90 (m, 3H), 7.51 (dd, *J* = 8.5, 2.2 Hz, 1H), 7.32 – 7.26 (m, 2H), 7.10 (d, *J* = 1.9 Hz, 1H), 4.34 (s, 2H), 3.96 (s, 3H,), 2.04 (s, 3H);* **^{13}C NMR (101 MHz, DMSO-d6)** δ 168.6, 167.1, 157.3, 149.2, 142.9, 142.6, 139.3, 136.0, 132.8, 131.9, 130.6, 130.1, 128.5, 121.4, 120.2, 118.2, 114.9, 112.4, 99.4, 58.5, 56.0, 50.7, 50.3, 21.0; **HRMS** m/z calcd for $C_{28}H_{28}N_4O_4$ $[M+H]^+$ 485.2183 found 485.2186; **IR (cm^{-1})** 2361, 167, 1636, 1428, 1265, 1178, 1118, 720; **HPLC T$_R$ (min)** 12.18 (5-95% ACN 0.1% TFA in H_2O 0.1% TFA over 20 minutes), 21.46 (5-95% ACN 0.1% TFA in H_2O 0.1% TFA over 50 minutes), 99% purity; *Piperazine protons missing due to amide rotamers, HSQC cross peaks at 3.39 ppm/50.66 ppm and 3.04 ppm/50.34 ppm (page 25). Please also see example high temperature NMR of compound **10** (page S23).

Thermal Shift

**Protein**	**Ligand**	**Tm B**
*Pf*CLK3 5µg	DMSO	43.44542
*Pf*CLK3 5µg	DMSO	43.62983
*Pf*CLK3 5µg	DMSO	43.537582
*Pf*CLK3 5µg	TCMDC-135051 (**1**)	66.66167
*Pf*CLK3 5µg	TCMDC-135051 (**1**)	66.66749
*Pf*CLK3 5µg	TCMDC-135051 (**1**)	66.64996
*Pf*CLK3 5µg	Compound **4**	63.6106
*Pf*CLK3 5µg	Compound **4**	63.703854
*Pf*CLK3 5µg	Compound **4**	63.637234

Table S2: Thermal shift data for compounds **1** and **4**

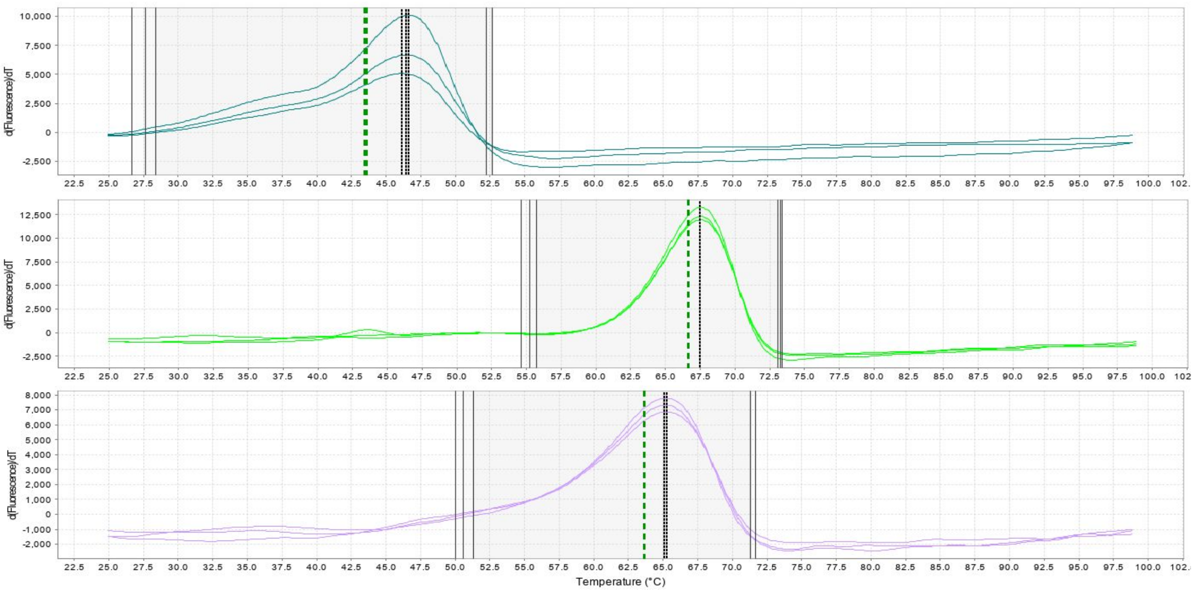

Figure S1: Melting curves for *Pf*CLK3 in presence of DMSO (teal), TCMDC-135051 (green) and compound **4** (purple)

Parasiticidal data for compound 12

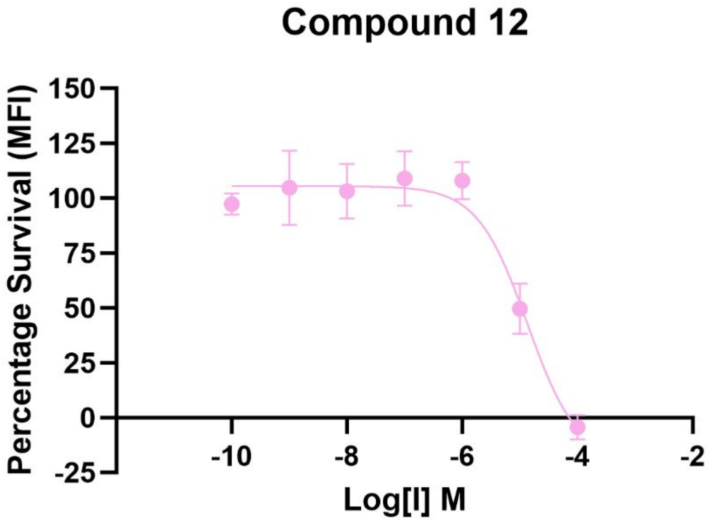

Figure S2: Parasiticidal activity of compound **12** vs 3D7 parasites.

Metabolic Stability

Stability in human serum was evaluated using HPLC. To an Eppendorf containing 15 µL compound (10 mM in DMSO) in a pre-heated block (37 °C) was added 135 µL PBS buffer and 150 µL to initiate the reaction (0.5 mM compound and 5% DMSO end concentration). The reaction was incubated at 37 °C. At each time point (2 minutes – 24 hours), 12.5 µL was removed and 87.5 µL of cold MeOH (-20 °C, 2% TFA) was added to quench the reaction. Samples were centrifuged (1 minute, 4000 g), and 10 µL was then injected onto a Shimadzu

HPLC to run over 15 minutes (5-95% ACN +0.1% TFA in H_2O +0.1% TFA). Consumption of starting material was then monitored by UV (compound **4** was monitored by a wavelength of 214 nm, while compound **12** was monitored by 254 nm due to its retention time being similar to that of the residual serum peak). The natural log of the peak area was plotted against time to yield a straight line (Fig. S5). The gradient of this line was taken as the pseudo-first order rate constant, and $t_{1/2}$ obtained from equation 1, giving a half-life for compounds 4 and 12 of 12.83 and 2.96 hours respectively

Equation 1: $$\frac{-\ln(2)}{K}$$

Time (min)	Area	Ln(Area)
5	676158	13.424182
10	692085	13.447464
20	709503	13.47232
40	766903	13.550116
120	284696	12.559177
240	185778	12.132308
1440	2414	7.7890404

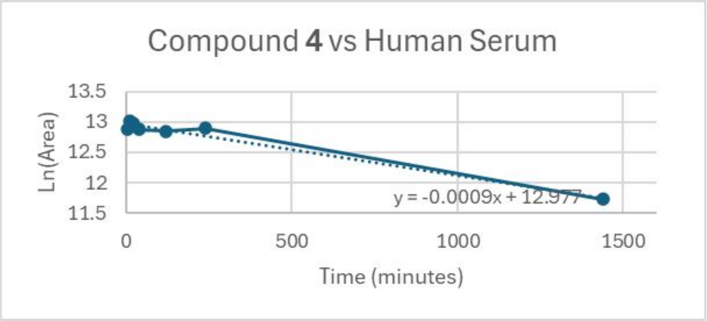

Time (min)	Area	Ln(Area)
5	676158	13.424182
10	692085	13.447464
20	709503	13.47232
40	766903	13.550116
120	284696	12.559177
240	185778	12.132308
1440	2414	7.7890404

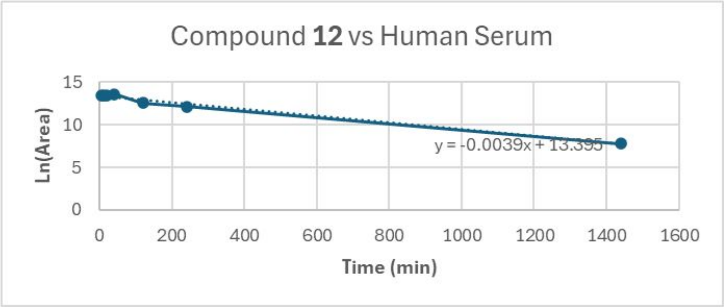

Figure S3: Raw data for human serum stability of compounds **4** and **12.**

Glutathione stability was determined using HPLC. To an Eppendorf containing 125 µL compound (10 mM in DMSO) in a pre-heated block (37 °C) was added 1125 µL GSH (11.1 mM in 5 mM phosphate buffer, pH 7.5) to initiate the reaction. The reaction was incubated at 37 °C, and 100µL aliquots were removed at time points (2 minutes – 72h). 900µL cold MeOH (-20 °C, 0.1% formic acid) was added to each aliquot to quench the reaction. 40 µL was then injected onto a Shimadzu HPLC to run over 15 minutes (5-95% ACN +0.1% TFA in H_2O +0.1% TFA). Consumption of starting material was then monitored by UV (214 nm) and the natural log of the peak area was plotted against time to yield a straight line. The gradient of this line was taken as the pseudo-first order rate constant, and $t_{1/2}$ obtained from equation 1. Data for negative control compound **12** is also plotted in Figure S4.

Time (min)	Area	Ln(Area)
2	842047	13.643591
5	769858	13.553961
10	515365	13.152631
15	369653	12.82032
20	247871	12.420664
25	153753	11.943103
30	111962	11.625915
40	60206	11.005527
50	23814	10.078029
60	7244	8.8879288
70	2055	7.6280311

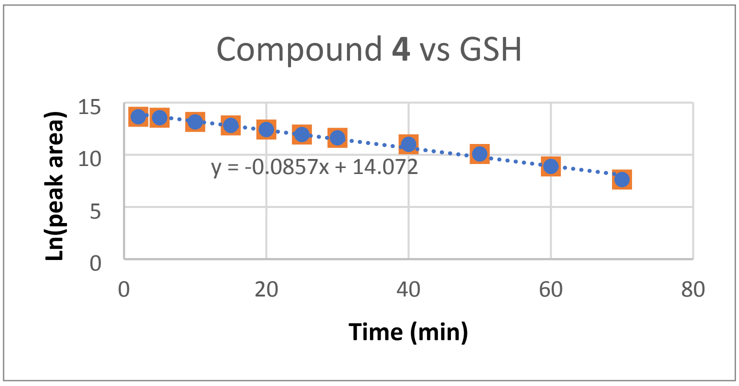

Time (min)	Area	Ln(Area)
10	3913738	15.1800035
15	4267762	15.2666001
20	4365076	15.2891462
30	3751064	15.1375501
40	3566175	15.0870042
60	4239701	15.2600033
120	3908743	15.1787264
240	4014926	15.2055295
360	4061053	15.2169529
1500	4391148	15.2951013
2880	3833500	15.1592888
4440	3534668	15.0781299

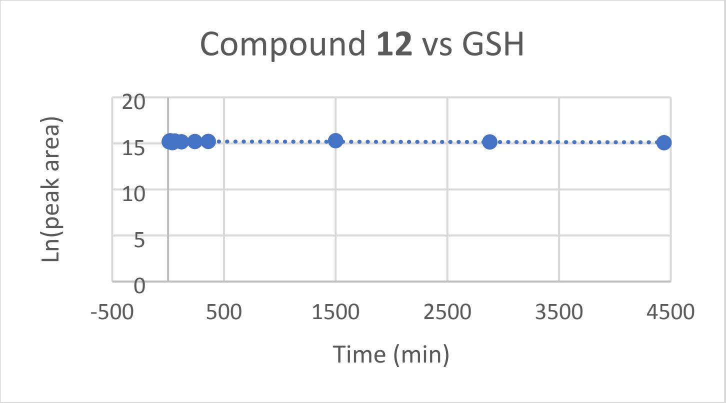

Figure S4: Raw data for GSH stability of compounds **4** and **12.**

Stability in microsomes and hepatocytes were carried out by Pharmaron Ltd. For microsomal stability, two separated experiments were performed as follows. a) With Cofactors (NADPH): 25 µL of 10 mM NADPH was added to the incubations. The final concentrations of microsomes and NADPH were 0.5 mg/mL and 1 mM, respectively. b) Without Cofactors (NADPH): 25 µL of 100 mM Phosphate buffer was added to the incubations. The final concentration of microsomes was 0.5 mg/mL. The mixture was pre-warmed at 37 °C for 10 minutes.

The reaction was started with the addition of 2.5 µL of 100 µM control compound or test compound solutions. Verapamil was used as positive control in this study. The final concentration of test compound or control compound was 1 µM. The incubation solution was incubated in water batch at 37°C.

Aliquots of 30 µL were taken from the reaction solution at 0.5, 5, 15, 30 and 60 minutes. The reaction was stopped by the addition of 5 volumes of cold acetonitrile with IS (100 nM alprazolam, 200 nM caffeine and 100 nM tolbutamide). Samples were centrifuged at 3, 220 g for 40 minutes. Aliquot of 100 µL of the supernatant was mixed with 100 µL of ultra-pure H_2O and then used for LC-MS/MS analysis.

Peak areas were determined from extracted ion chromatograms. The slope value, k, was determined by linear regression of the natural logarithm of the remaining percentage of the parent drug vs. incubation time curve. The half-life was obtained from equation 1 using a mean of duplicate values and in vitro intrinsic clearance (in vitro CLint, in µL/min/mg protein) was obtained from equation 2.

Equation 2: $$CL_{int} = -k \times \frac{volume\ of\ incubation\ (\mu L)}{mass\ of\ proteins\ (mg)}$$

**Compound ID**	**Species**	**Assay Format**	**Remaining Percentage (%)**				
			**0.5 min**	**5 min**	**15 min**	**30 min**	**60 min**
**Verapamil**	**Human**	+Cofactors	100.00	52.41	16.38	7.46	2.01
		-Cofactors	100.00	-	-	-	106.43
	**Mouse**	+Cofactors	100.00	30.49	7.45	1.55	BLOD
		-Cofactors	100.00	-	-	-	100.85
**4**	**Human**	+Cofactors	100.00	92.71	80.89	62.67	35.15
		-Cofactors	100.00	-	-	-	51.04
	**Mouse**	+Cofactors	100.00	85.84	54.81	33.01	12.14

		-Cofactors	100.00	-	-	-	30.47

Table S3: Raw data for microsomal stability of compound **4**.

For hepatocyte stability, 198 µL of hepatocytes and boiled hepatocytes in William's E Medium supplemented with GlutaMAX were added to a 96-well non-coated plate, and incubated at 37°C for 10 minutes. 2 µL of 100 µM test compound or positive control was added start the reaction, followed by further incubation. Well contents were transferred in 25 µL aliquots at time points of 0.5, 15, 30, 60, 90 and 120 minutes. The aliquots were then mixed with 6 volumes (150 µL) of acetonitrile containing with internal standard, IS (100 nM alprazolam, 200 nM caffeine and 100 nM tolbutamide) to terminate the reaction. Samples were vortexed for 5 minutes and centrifuged for 45 minutes at 3,220 g. 100 µL of the supernatant was diluted in 100 µL ultra-pure water, and the mixture was used for LC/MS/MS analysis. All incubations were performed in duplicate.

Peak areas were determined from extracted ion chromatograms. The slope value, k, was determined by linear regression of the natural logarithm of the remaining percentage of the parent drug vs. incubation time curve. The half-life was obtained from equation 1 using a mean of duplicate values and in vitro intrinsic clearance (in vitro CLint, in µL/min/1x10^6 cells) was obtained from equation 3.

Equation 3: $$CL_{int} = -k \times \frac{volume\ of\ incubation\ (\mu L)}{number\ of\ hepatocytes\ per\ well}$$

**Compound ID**	**Species**	**Remaining Percentage (%)**					
		**0.5 min**	**15 min**	**30 min**	**60 min**	**90 min**	**120 min**
**Verapamil**	**Mouse**	100.00	22.09	7.27	2.23	BLOD	BLOD
**4**		100.00	20.48	3.71	BLOD	BLOD	BLOD
**Verapamil**		100.00	11.13	2.89	BLOD	BLOD	BLOD
**12**		100.00	110.00	98.33	86.67	77.83	63.13

Table S4: Raw data for hepatocyte stability of compounds **4** and **13**.

	**GSH $t_{1/2}$**	**MLM $t_{1/2}/CL_{int}$**	**HLM $t_{1/2}/CL_{int}$**	**MLH $t_{1/2}/CL_{int}$**
**4**	8.1 min	20 min/ 71μL /min/mg	40 min/ 35 μL/min/mg	6 min/ 218 μL/min/10^6 cells
**12**	>72h	--	--	165 min/ 8.4 μL/min/10^6 cells
**1**	>72h	--	>184 min/ <7.5 μL/min/mg	113 min/ 12.3 μL/min/10^6 cells

Table S5: Metabolic stability data for compounds 1, 4 and 16. MLM = mouse liver microsomes, HLM = liver microsomes, MLH = mouse liver hepatocytes, CL_{int} = intrinsic clearance.

Selectivity

	Compound **4** (1 μM)
Abl(h)	85
ALK(h)	50
AMPKα1(h)	94
ASK1(h)	81
Aurora-A(h)	98
CaMKI(h)	87
CDK1/cyclinB(h)	101
CDK2/cyclinA(h)	102
CDK6/cyclinD3(h)	99
CDK7/cyclinH/MAT1(h)	104
CDK9/cyclin T1(h)	23
CHK1(h)	93
CK1γ1(h)	106
CK2α2(h)	43
c-RAF(h)	93
DRAK1(h)	36

eEF-2K(h)	86
EGFR(h)	77
EphA5(h)	105
EphB4(h)	96
Fyn(h)	72
GSK3β(h)	92
IGF-1R(h)	31
IKKα(h)	102
IRAK4(h)	74
JAK2(h)	112
KDR(h)	81
LOK(h)	59
Lyn(h)	91
MAPKAP-K2(h)	103
MEK1(h)	95
MLK1(h)	73
Mnk2(h)	40
MSK2(h)	110
MST1(h)	93
mTOR(h)	93
NEK2(h)	101
p70S6K(h)	111
PAK2(h)	105
PDGFRβ(h)	104
Pim-1(h)	47
PKA(h)	98
PKBα(h)	119
PKCα(h)	56
PKCθ(h)	93
PKG1α(h)	78
Plk3(h)	108
PRAK(h)	89
ROCK-I(h)	108
Rse(h)	111
Rsk1(h)	91
SAPK2a(h)	102
SRPK1(h)	88
TAK1(h)	97

PI3 Kinase (p110β/p85α)(h)	95
PI3 Kinase (p120γ)(h)	95
PI3 Kinase (p110δ/p85α)(h)	70
PI3 Kinase (p110α/p85α)(h)	97

Table S6: Normalized selectivity data for compound **4**.

	TCMDC-135051 (1uM)
Abl(h)	68
ALK(h)	30
AMPKα1(h)	46
ASK1(h)	92
Aurora-A(h)	11
CaMKI(h)	100
CDK1/cyclinB(h)	15
CDK2/cyclinA(h)	30
CDK6/cyclinD3(h)	82
CDK7/cyclinH/MAT1(h)	102
CDK9/cyclin T1(h)	6
CHK1(h)	101
CK1γ1(h)	85
CK2α2(h)	39
c-RAF(h)	106
DRAK1(h)	83
eEF-2K(h)	100
EGFR(h)	95
EphA5(h)	97
EphB4(h)	104
Fyn(h)	49
GSK3β(h)	46
IGF-1R(h)	94
IKKα(h)	93
IRAK4(h)	27

JAK2(h)	20
KDR(h)	61
LOK(h)	37
Lyn(h)	28
MAPKAP-K2(h)	94
MEK1(h)	100
MLK1(h)	92
Mnk2(h)	38
MSK2(h)	100
MST1(h)	84
mTOR(h)	100
NEK2(h)	105
p70S6K(h)	100
PAK2(h)	110
PDGFRβ(h)	94
PI3 Kinase (p110a/p85a)(h)	100
PI3 Kinase (p110b/p85a)(h)	99
PI3 Kinase (p110d/p85a)(h)	73
PI3 Kinase (p120g)(h)	96
Pim-1(h)	101
PKA(h)	101
PKBα(h)	106
PKCα(h)	73
PKCθ(h)	108
PKG1α(h)	105
Plk3(h)	100
PRAK(h)	74
ROCK-I(h)	117
Rse(h)	109
Rsk1(h)	105
SAPK2a(h)	105
SRPK1(h)	105
TAK1(h)	45

Table S7: Normalized selectivity data for TCMDC-135051 (**1**).

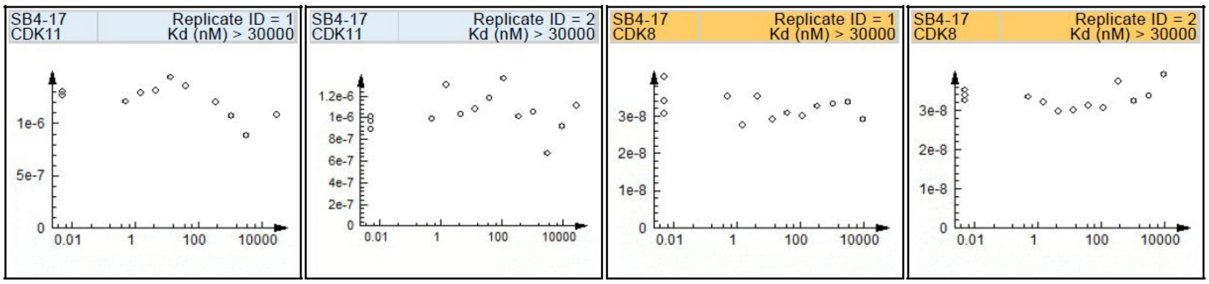

Figure S5: KINOMEscan™ curves of compound **4** with CDK11 and CDK8. The amount of kinase measured by qPCR (Signal; y-axis) is plotted against the corresponding compound concentration in nM in log10 scale (x-axis)

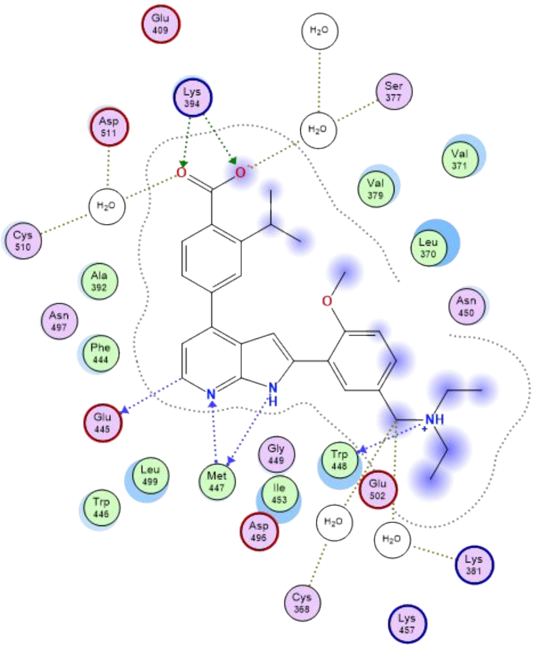

Figure S6: Ligand interaction map of co-crystal structure of TCMDC-135051 and *Pf*CLK3

NMR Spectra for Novel Compounds

^{1}H and ^{13}C Spectra ($CDCl_3$) for compound 9

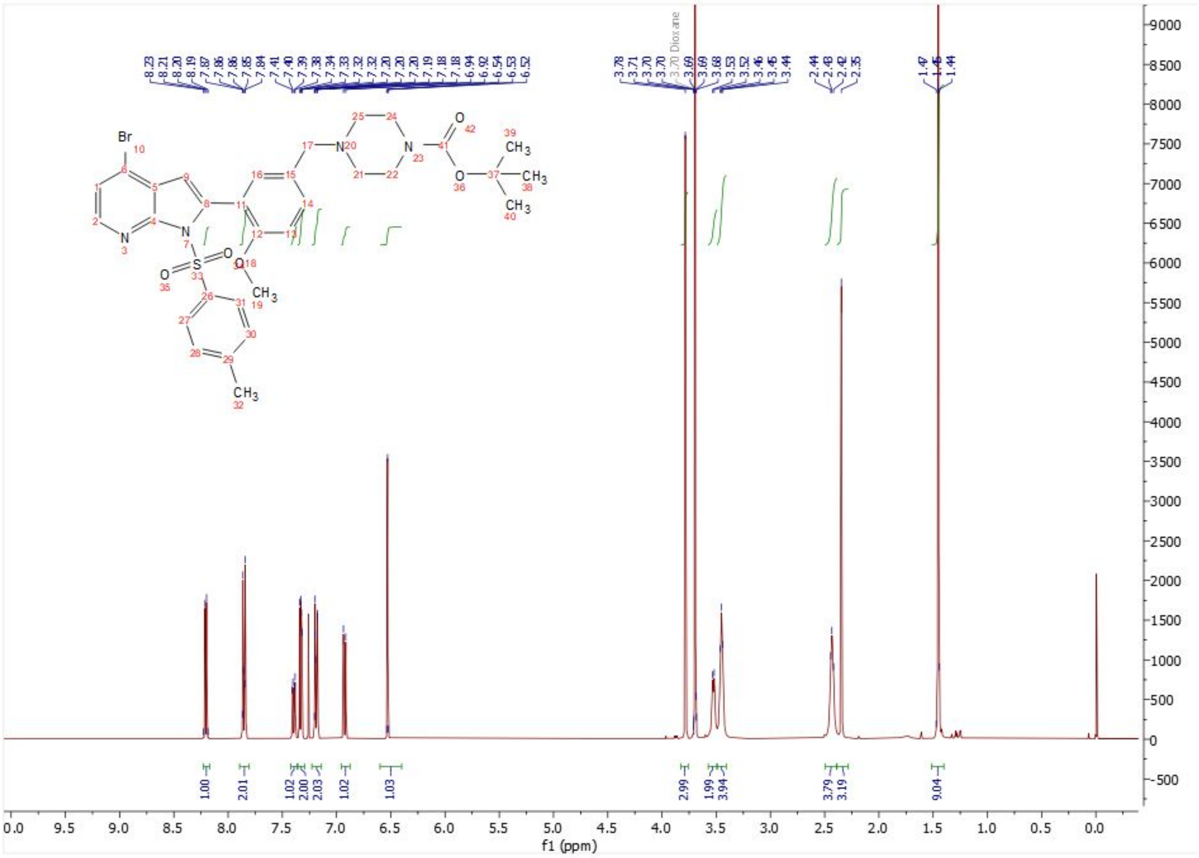

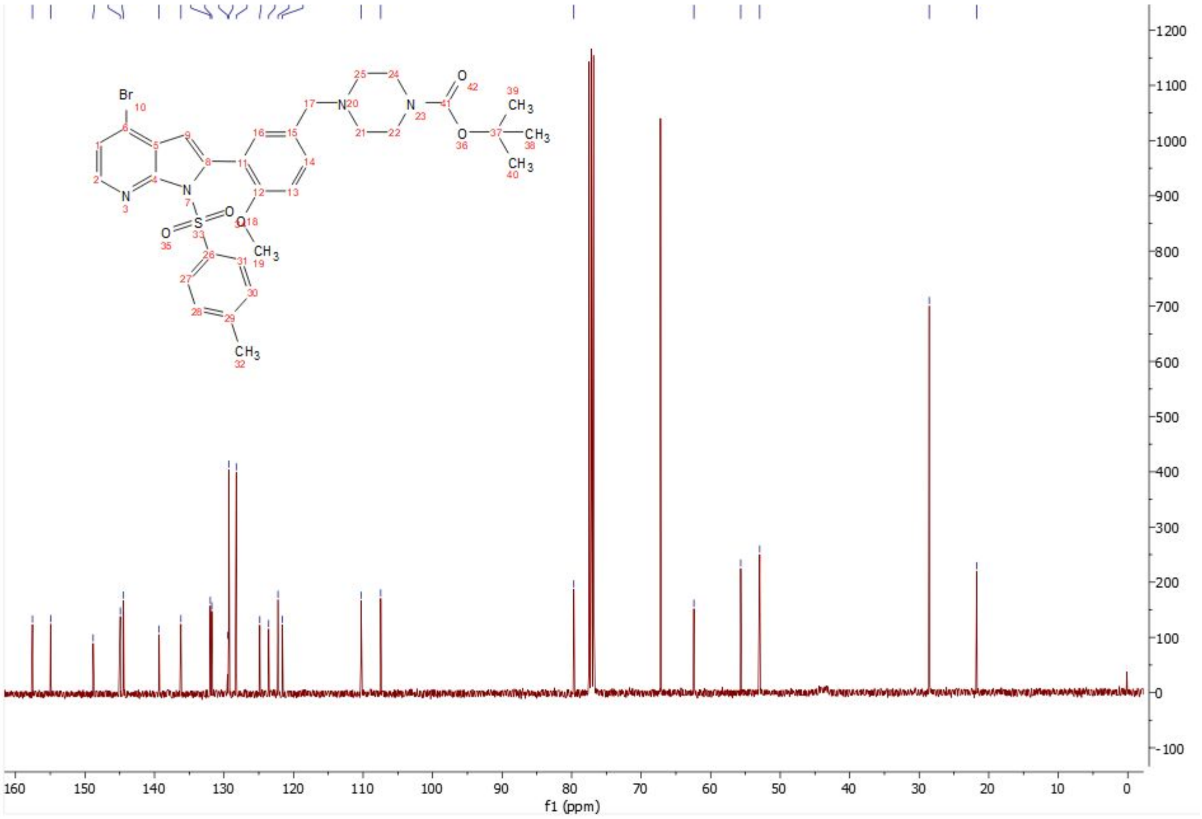

^{1}H and ^{13}C Spectra ($CDCl_3$) for compound 10

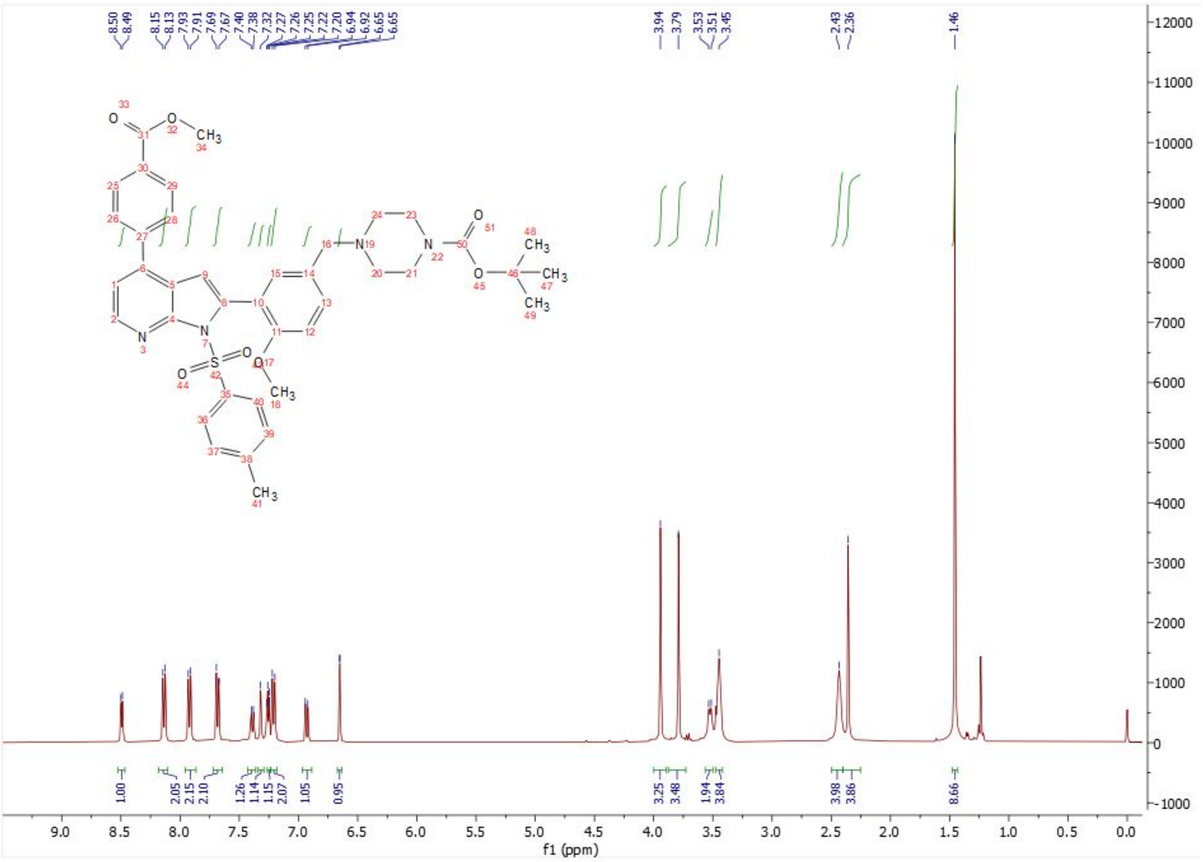

^{13}C Spectra (100 ℃, $CDCl_3$) for compound 10 [piperazine rotomer signals resolved at

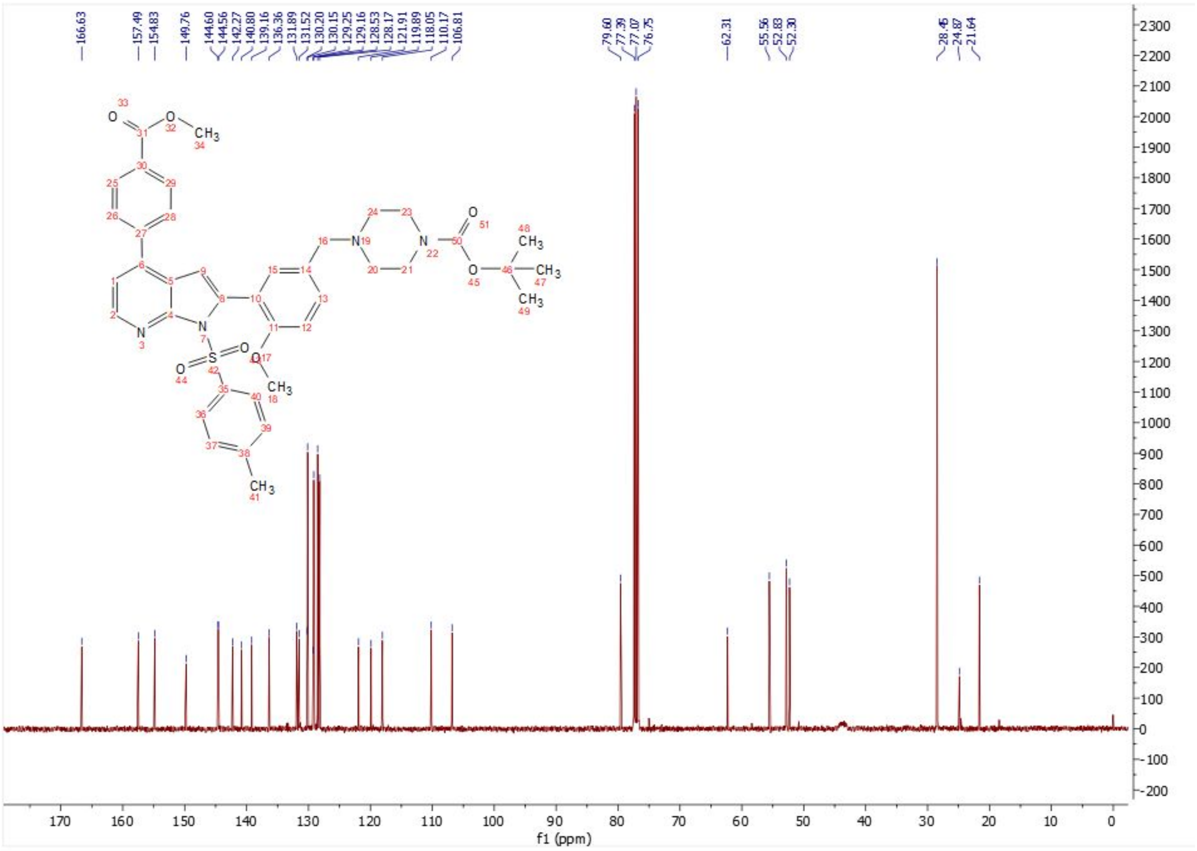

43.8 ppm]

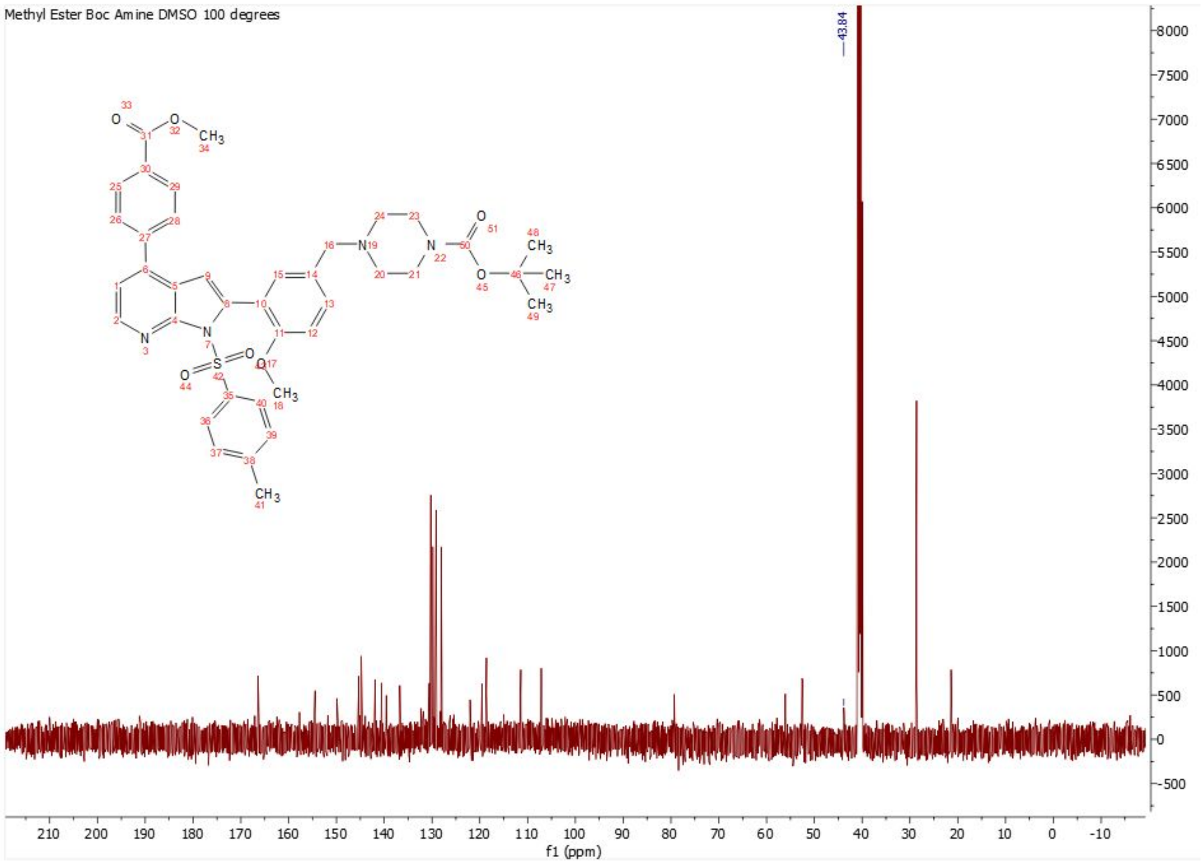

^{1}H and [13]C Spectra (DMSO-d6) for compound 2

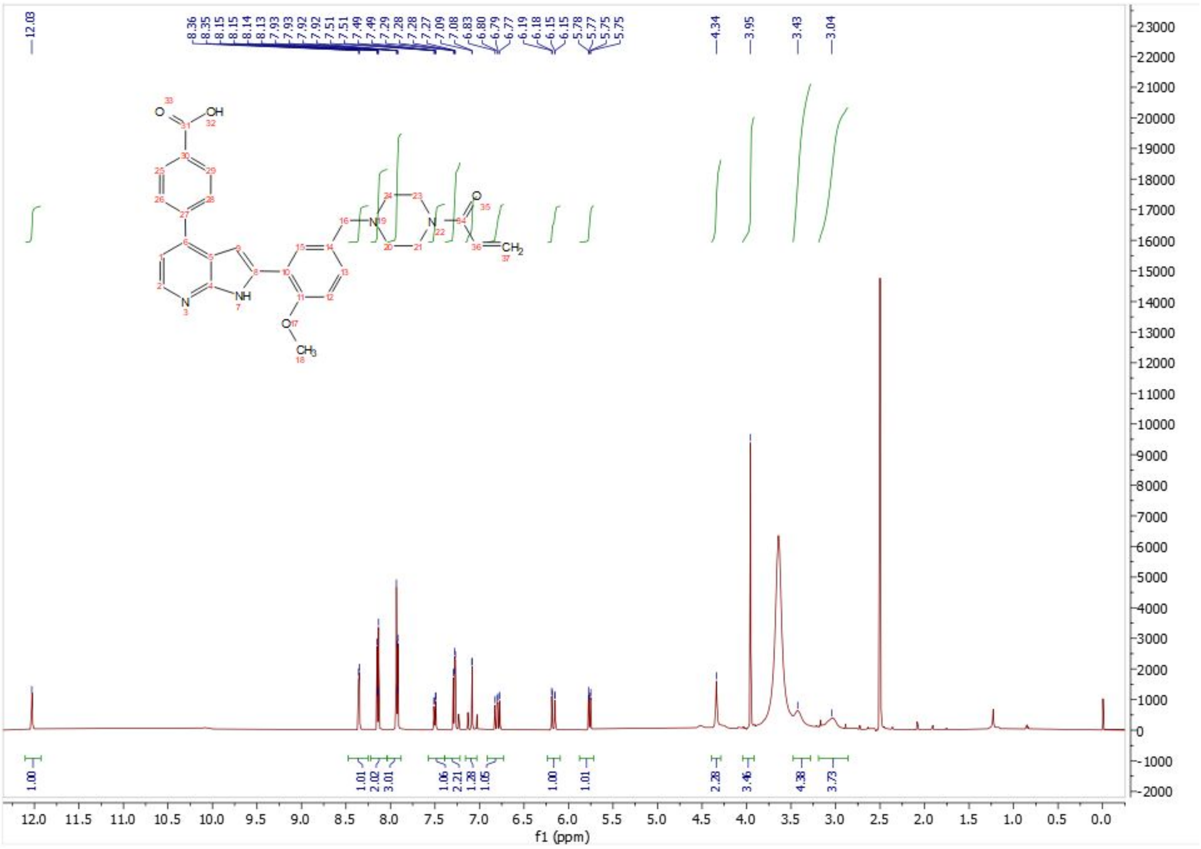

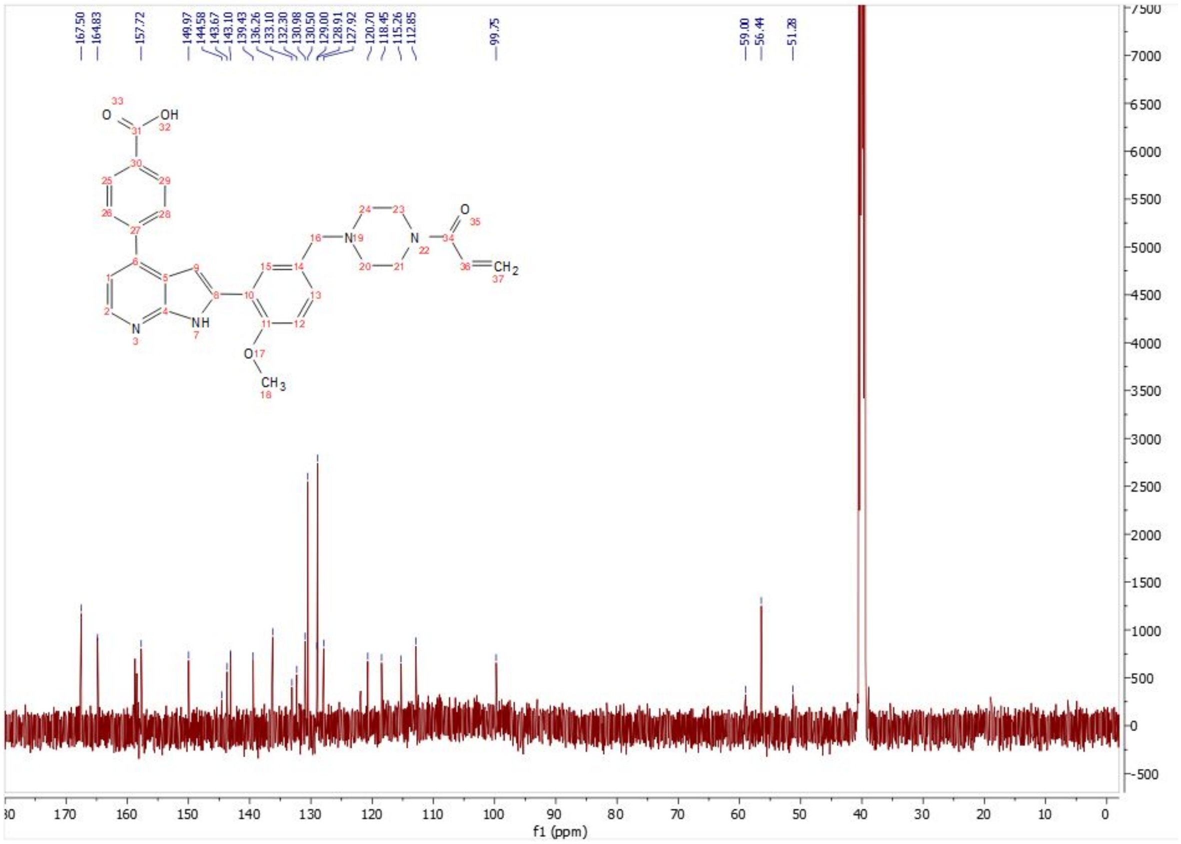

^{1}H and ^{13}C Spectra (DMSO-d6) for compound 3

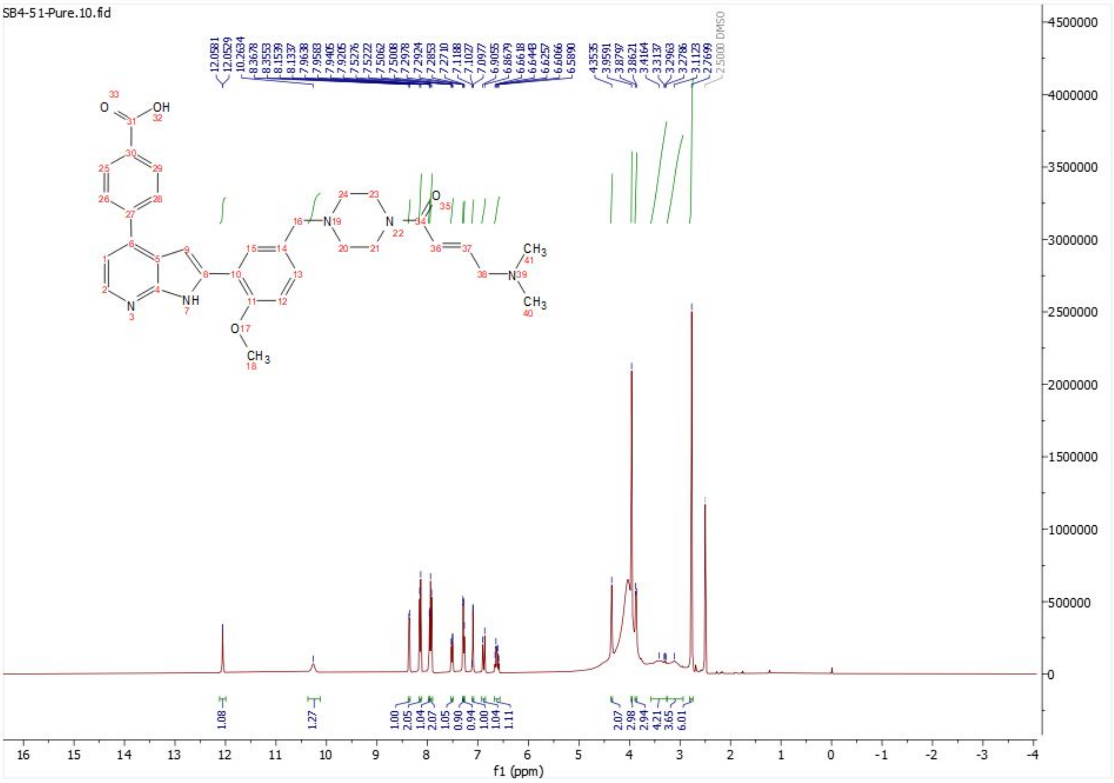

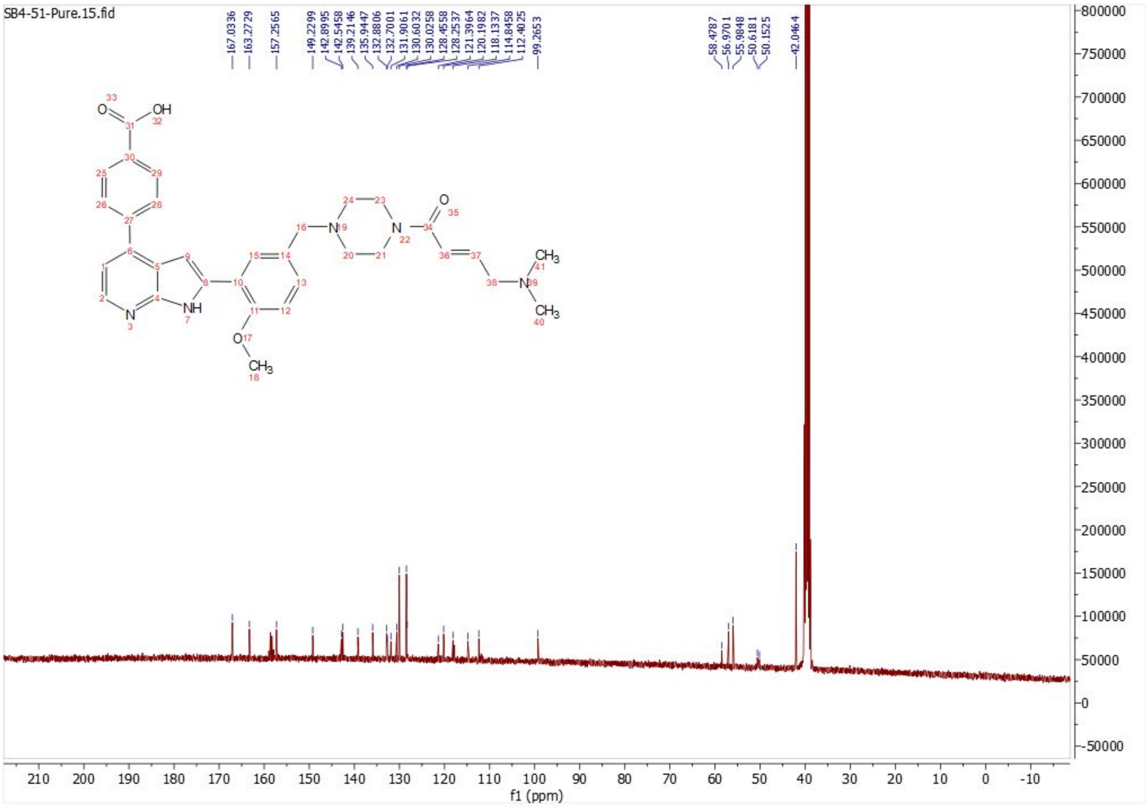

^{1}H and ^{13}C Spectra (DMSO-d6) for compound 4

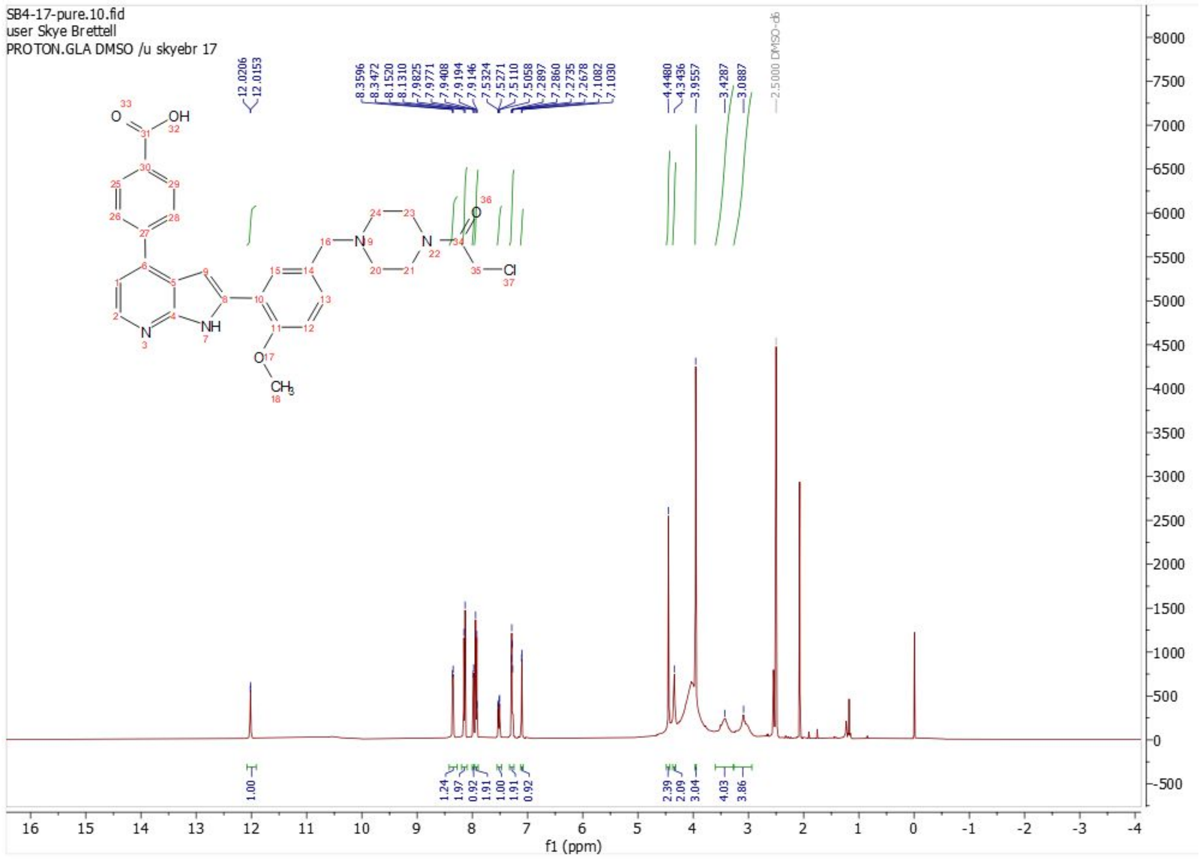

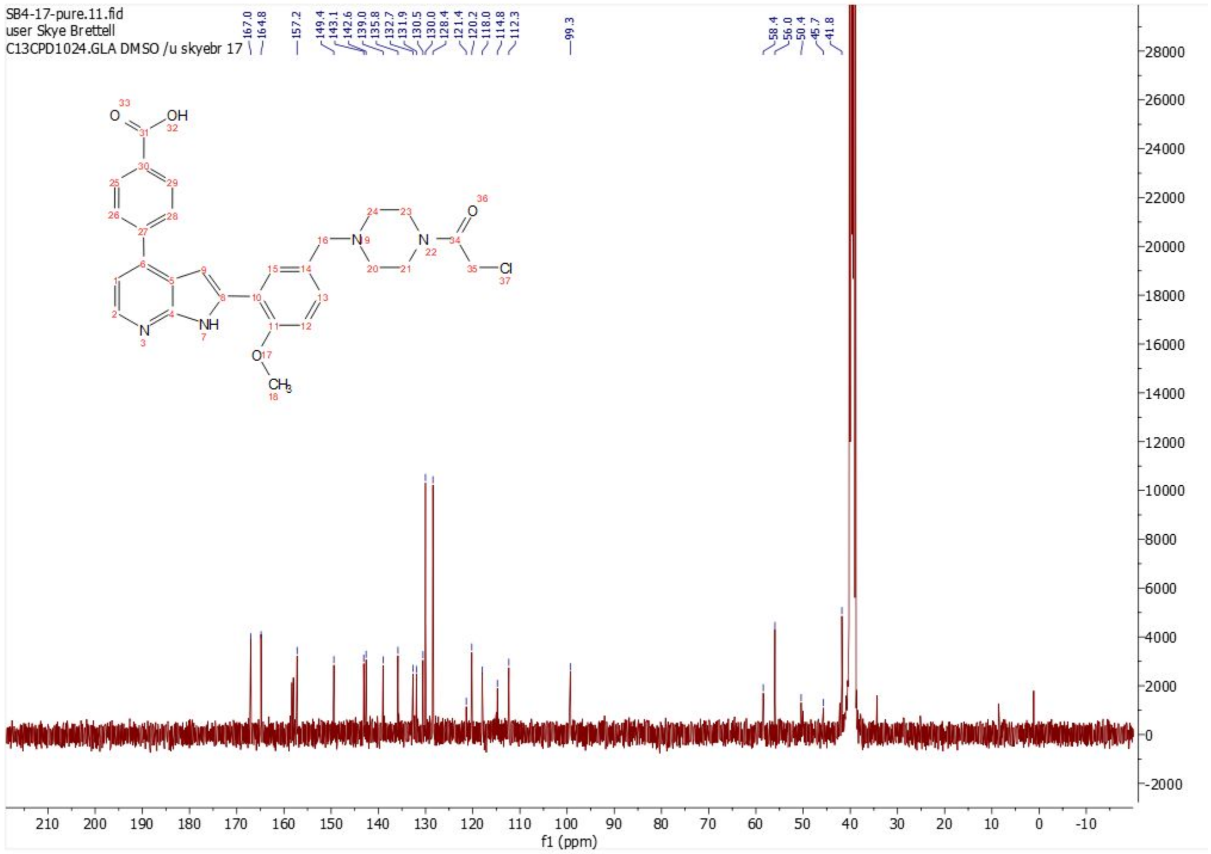

^{1}H, ^{13}C and HSQC Spectra (DMSO-d6) for compound 12

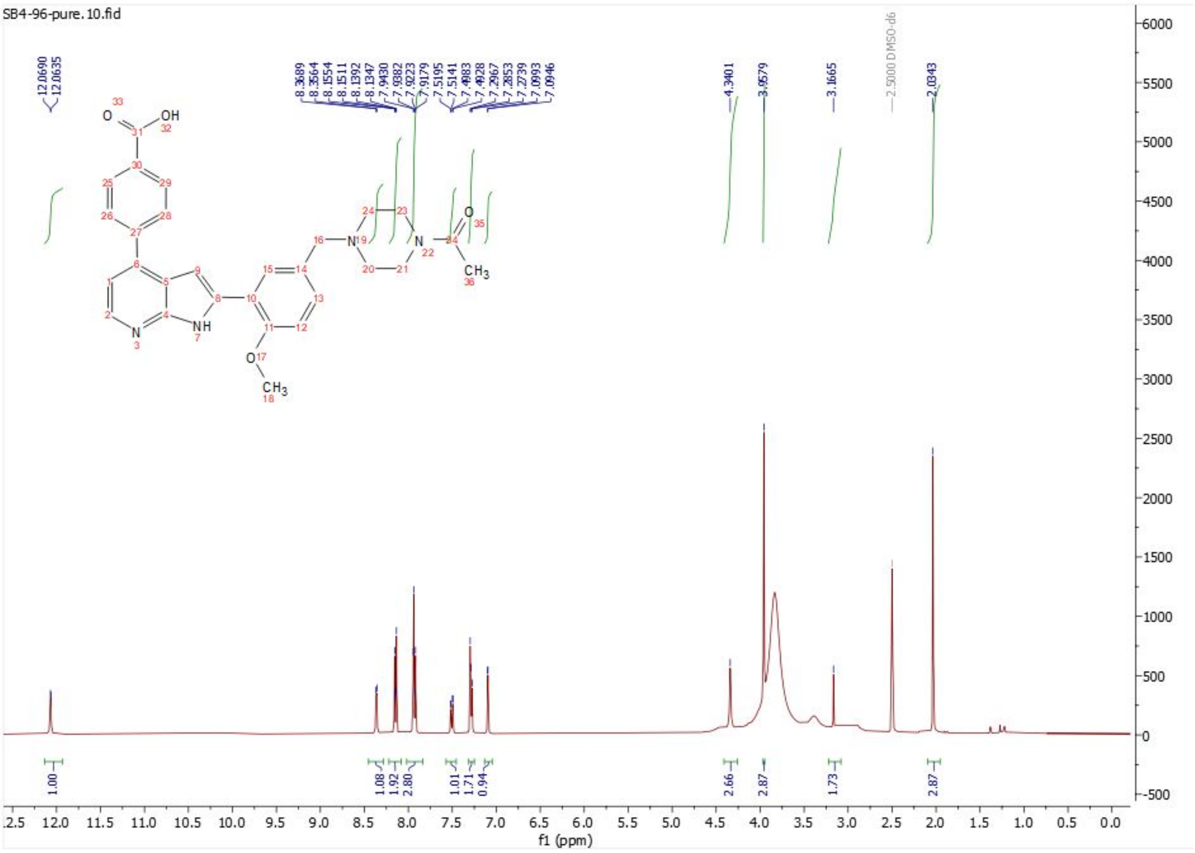
SB4-96-pure.10.fid
12.0690
12.0635
8.3689
8.3564
8.1554
8.1511
8.1392
8.1347
7.9430
7.9382
7.9223
7.9179
7.5195
7.5141
7.4983
7.4928
7.2967
7.2853
7.2739
7.0993
7.0946
4.3401
3.9579
3.1665
2.5000 DMSO-d6
2.0343
1.00
1.08
1.92
2.80
1.01
1.71
0.94
2.66
2.87
1.73
2.87
f1 (ppm)

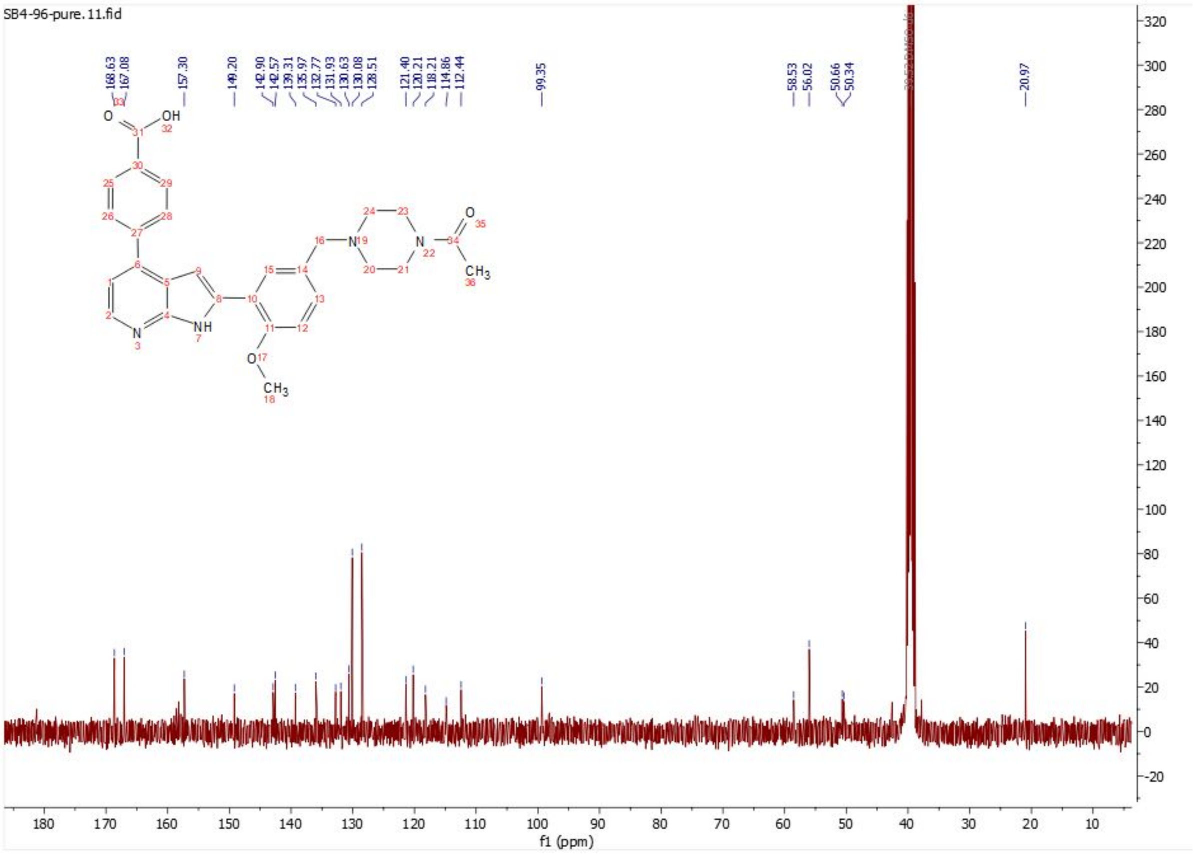
SB4-96-pure.11.fid
168.63
167.08
157.30
149.20
142.90
142.57
139.31
135.97
132.77
131.93
130.63
130.08
128.51
121.40
120.21
118.21
114.86
112.44
99.35
58.53
56.02
50.66
50.34
20.97
f1 (ppm)

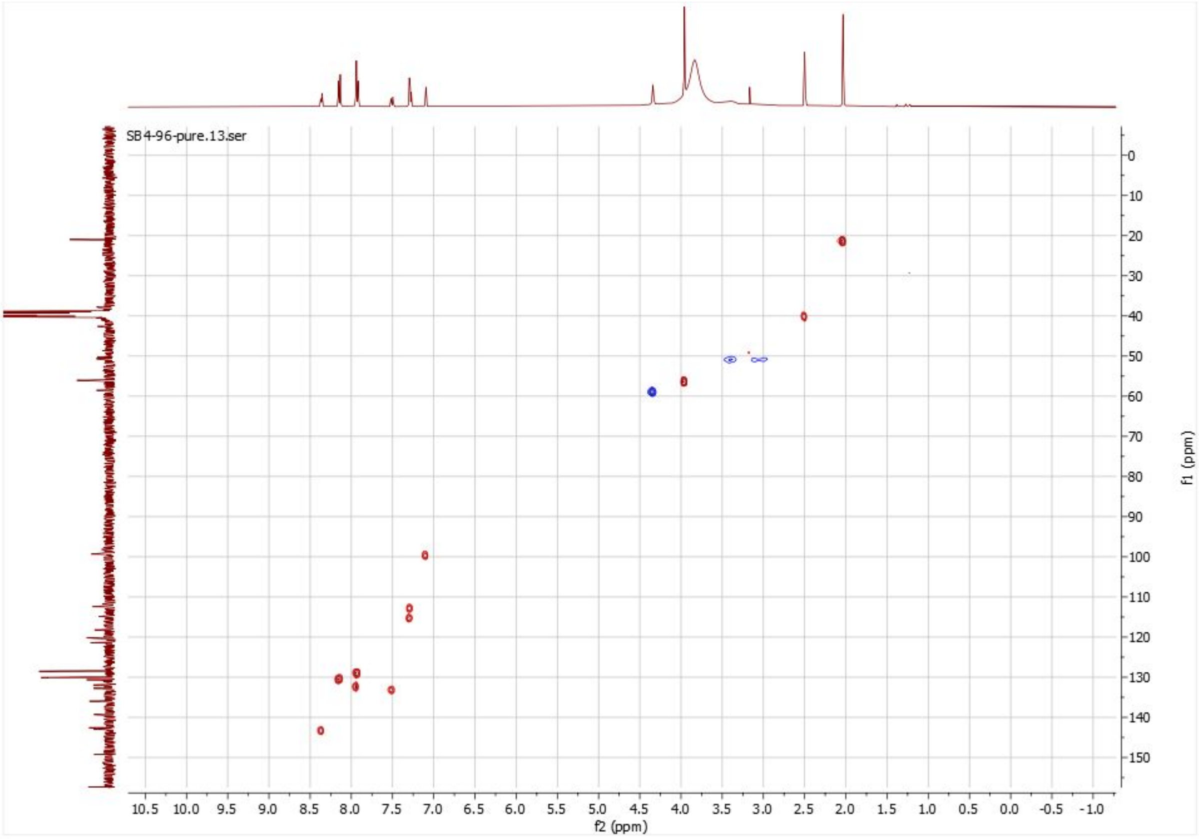

HPLC Traces and Purity for Final Compounds

HPLC Data for Compound 2

(5-95% ACN 0.1% TFA in H_2O 0.1% TFA over 20 minutes)

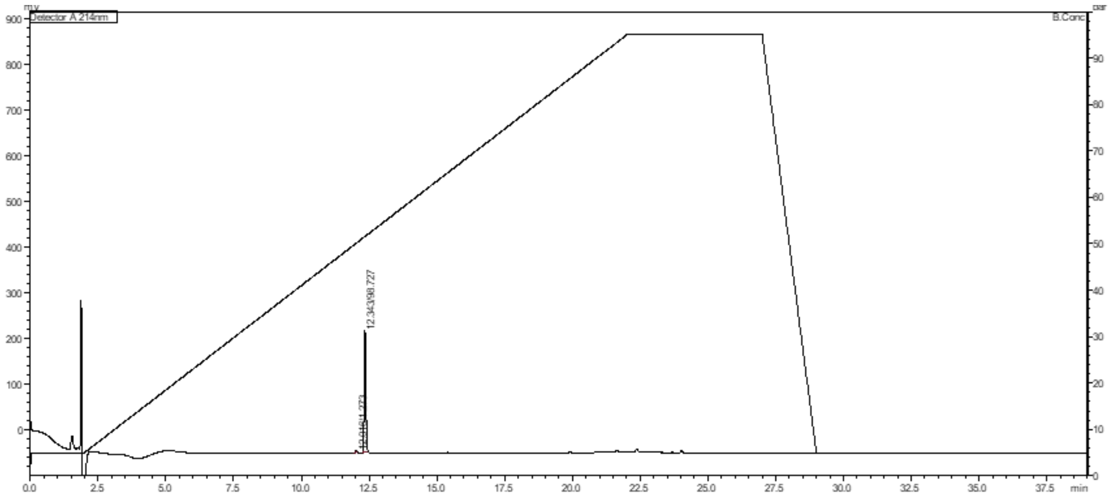

(5-95% ACN 0.1% TFA in H_2O 0.1% TFA over 50 minutes)

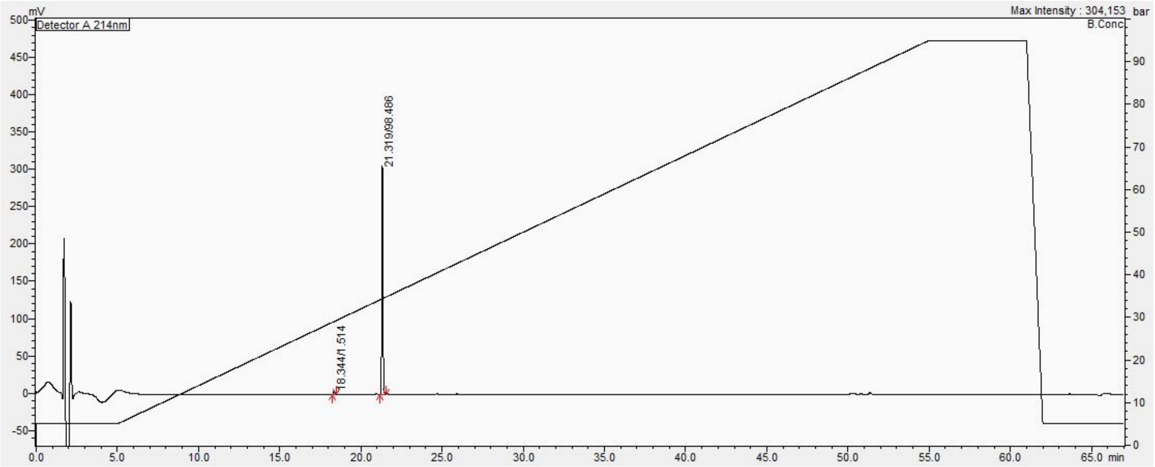

HPLC Data for Compound 3

(5-95% ACN 0.1% TFA in H_2O 0.1% TFA over 20 minutes)

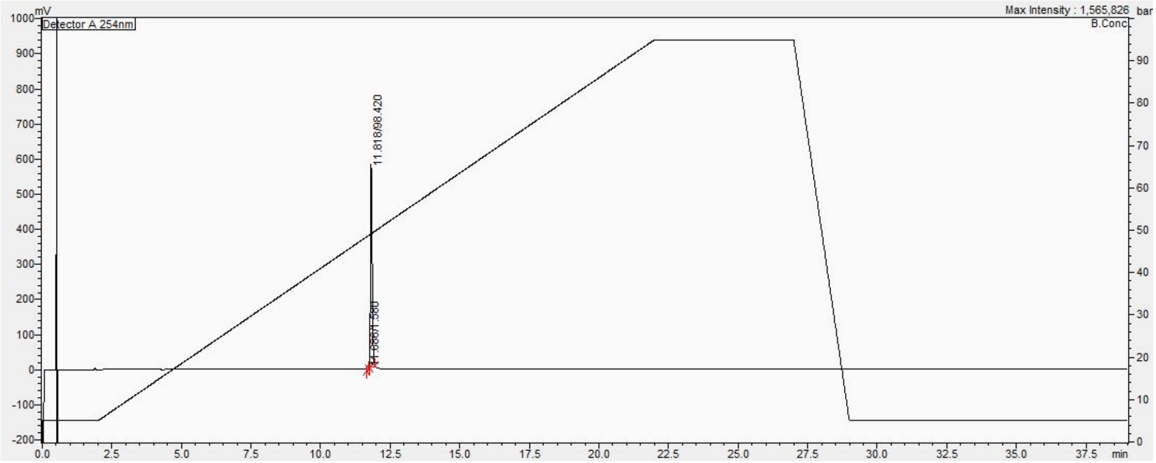

(5-95% ACN 0.1% TFA in H_2O 0.1% TFA over 50 minutes)

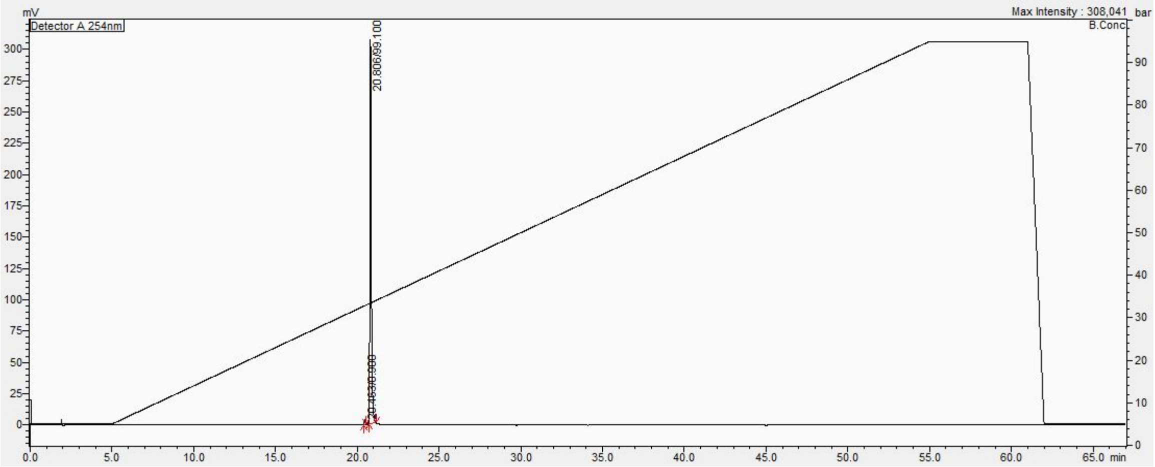

HPLC Data for Compound 4

(5-95% ACN 0.1% TFA in H_2O 0.1% TFA over 20 minutes)

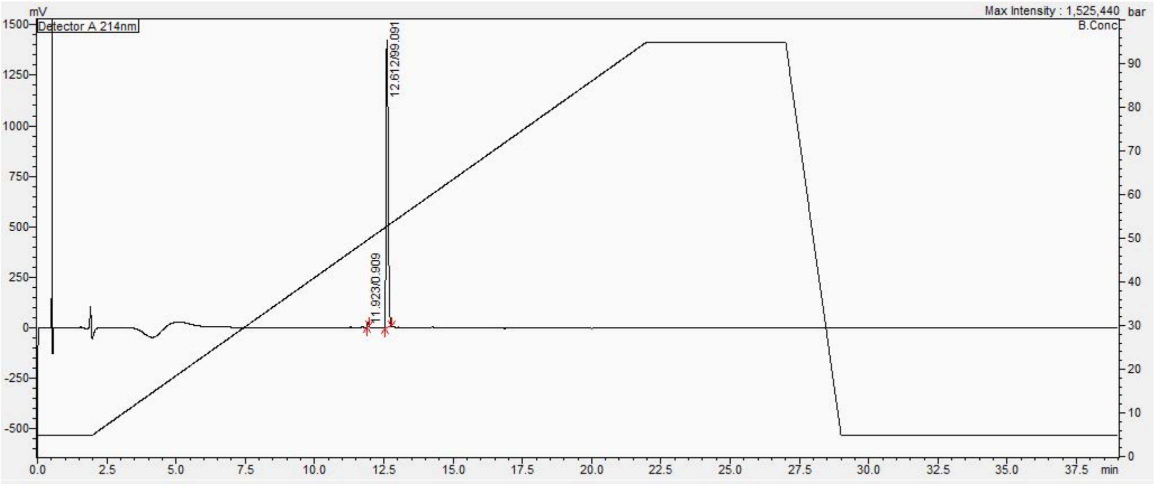

(5-95% ACN 0.1% TFA in H_2O 0.1% TFA over 50 minutes)

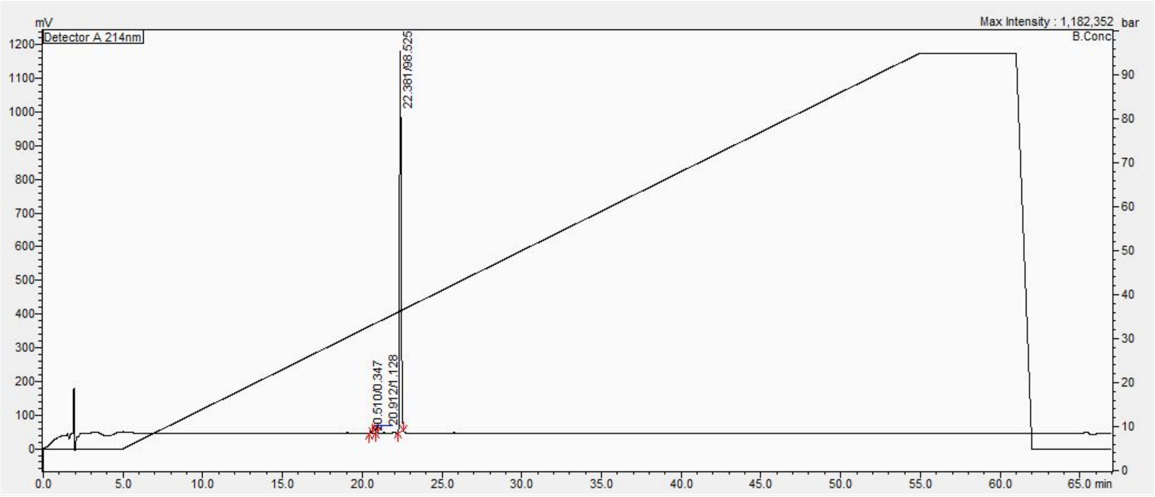

HPLC Data for Compound 12

(5-95% ACN 0.1% TFA in H_2O 0.1% TFA over 20 minutes)

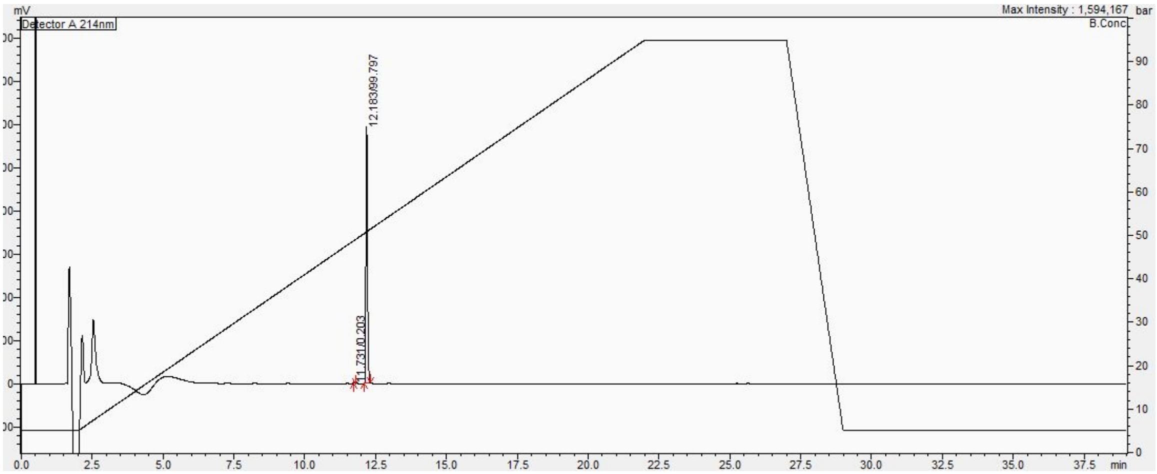

(5-95% ACN 0.1% TFA in H_2O 0.1% TFA over 50 minutes)

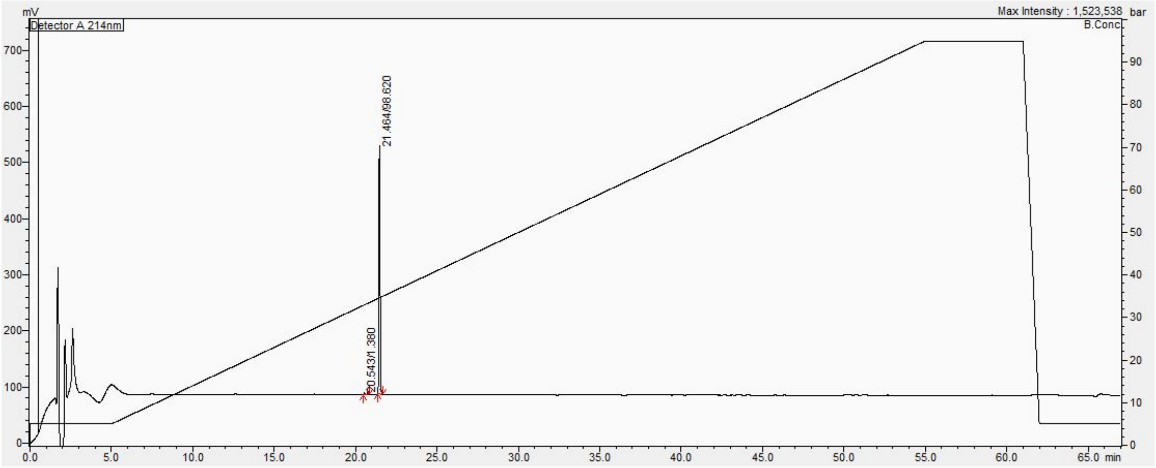

Supplement: Supplementary file 6 — jm4c01300_si_006.pdf [file jm4c01300_si_006.pdf]
